# Burden of Tracheal, Bronchus, and Lung Cancer Attributable to High Fasting Plasma Glucose

**DOI:** 10.34172/aim.33332

**Published:** 2025-05-01

**Authors:** Jingyi Zhou, Chenglong Xi, Zhiyu Luan, Lufeng Mao, Shiliang Ling

**Affiliations:** ^1^Department of Oncology, Ninghai Traditional Chinese Medicine Hospital, Ningbo, China; ^2^Department of Oncology, Ningbo Municipal Hospital of Traditional Chinese Medicine, Affiliated Hospital of Zhejiang Chinese Medical University, Ningbo, China

**Keywords:** Annual estimated percentage change, Global Burden of Disease Study, Disability-adjusted life years, High fasting plasma glucose (HFPG), Tracheal, bronchus, and lung (TBL) cancer

## Abstract

**Background:**

We aimed to evaluate the situation and change trends in the tracheal, bronchus, and lung (TBL) cancer burden attributable to high fasting plasma glucose (HFPG) stratified by gender, age, region, country, and sociodemographic index (SDI).

**Methods:**

We evaluated the age-standardized death rate (ASDR) and disability-adjusted life years (DALYs) rate of TBL cancer attributable to HFPG and their corresponding estimated annual percentage change (EAPC) trends in 204 countries and 21 regions.

**Results:**

Globally from 1990 to 2019, the ASDR (EAPC=0.98; 95% confidence interval [CI]: 0.82–1.15) and age-standardized DALY rate (EAPC=0.68; 95% CI: 0.55–0.82) of TBL cancer attributable to HFPG trended upward. Furthermore, the steepest increment in age-standardized death and DALY rates were noted in low-SDI regions (EAPC=1.32; EAPC=1.35) and the North Africa/Middle East region (EAPC=2.66, ESPC=2.56) among all five SDI regions and 21 global geographic regions, respectively. Among the 204 countries, the highest growth rate in the ASDR was found in Georgia (EAPC=4.33, 95% CI: 3.66–5.00), and the highest growth rate in the age-standardized DALY rate occurred in Egypt (EAPC=4.34, 95% CI: 4.22–4.47). The highest ASDR and age-standardized DALY rate occurred in people over the age of 65 years, and in the 21 geographic regions, SDIs were negatively correlated with ASDRs and EAPCs in DALYs.

**Conclusion:**

The global burden of TBL cancer attributable to HFPG trended upward. The most significant increase in burden was observed in low-SDI regions and countries.

## Introduction

 Tracheal, bronchus, and lung (TBL) cancer ranks as the second-leading malignancy globally and emerged as the primary contributor to cancer mortality in 2020, constituting approximately 2.2 million incident cases and 1.8 million fatalities that year. The Cancer Tomorrow online gadget of the Global Cancer Observatory predicts that by 2040, there will be approximately 64% and 67% increases in the incidence and mortality of TBL cancer, respectively.^1^ TBL cancer therapeutics have witnessed remarkable progress through innovations in multimodal approaches in recent years. However, due to increases in population aging and environmental pollution, and the prevalence of unhealthy food habits, smoking, and other risk factors, the incidence and death rates of TBL cancer are increasing. Thus, the burden of TBL cancer maintains a public health problem of global concern.

 High fasting plasma glucose (HFPG) is a significant predictor, independent of confounders, for multiple non-communicable diseases. The global age-standardized average fasting plasma glucose (FPG) levels have increased by 0.07 mmol/L per decade in males and 0.09 mmol/L per decade in females.^2^ Unhealthy lifestyles, overweight, and obesity increase the incidence rates of HFPG and diabetes.^3,4^ Moreover, HFPG has become the third highest risk factor for death, as it contributes to more than 5.6 million deaths worldwide.^5^ HEPG serves as a prevalent risk factor for both disability and mortality, with additional significant associations observed across multiple cancer types, including TBL cancer, colorectal cancer, and breast cancer.^6,7^ Furthermore, HFPG is one of the most important manifestations of diabetes and thus an important determinant of the risk of diabetes. Some researchers have investigated the effects of HFPG or diabetes on the development, progression, complications, and prognosis of different types of cancer. In particular, it has been found that HFPG may contribute to cell proliferation and cancer progression,^8,9^ thereby posing a serious threat to longevity and quality of life.

 The Global Burden of Disease Study 2019 (GBD 2019) identified that TBL cancer was the most common cancer responsible for disability-adjusted life years (DALYs). Clinical or epidemiological investigations have shown that diabetes increases the death rate associated with lung cancer,^10^ and diabetic lung cancer patients exhibit poorer prognosis compared to those without diabetes.^11^ This is due to the considerable death rate and economic burden of TBL cancer attributable to HFPG.

 Studies have reported the cancer burden attributable to HFPG or diabetes but have not explored this association for specific types of cancer. Moreover, there is little global reporting on the burden of and trends in TBL cancer attributable to HFPG in different regions and countries. Accordingly, this research appraised GBD 2019 data to comprehensively evaluate the burden of and trend changes in TBL cancer attributable to HFPG in 204 countries and regions from 1990 to 2019. The conclusions may guide the development and implementation of public health interventions, and effective allocation of global health resources, in order to reduce the global burden of TBL cancer attributable to HFPG.

## Materials and Methods

###  Data 

 The Global Health Data Exchange Query Tool was utilized to search the GBD 2019 database (https://ghdx.healthdata.org/gbd-2019). Three hundred and sixty-nine diseases and injuries were analyzed and evaluated, together with 87 risk factors. Subsequently, the above-mentioned tool was used to extract data on TBL cancer (10^th^ version of the International Statistical Classification of Diseases and Related Health Problems codes 33–34) deaths, death rates, and DALYs attributable to HFPG from 204 countries and 21 regions from 1990 to 2019. Sociodemographic index (SDI) data were obtained for regions and countries (which are classified as low-, low-middle-, middle-, middle-high-, or high-SDI regions) and used to evaluate the relationship between SDIs and the burden of TBL cancer attributable to HFPG. The method for evaluating cancer burden in the GBD 2019 is described in a previous study.^12^ This research utilized anonymized open-source data, exempting it from institutional review board oversight.

 The FPG is a continuous variable quantified in millimoles per liter (mmol/L) and represents the average value for the target population. An HFPG refers to fasting plasma glucose concentrations above the established low-risk threshold range (4.8–5.4 mmol/L or 86.4–97.2 mg/dL).^2,13^

###  Statistical Analysis

 This study aimed to determine the age-standardized TBL cancer burden attributable to HFPG in the GBD 2019 in different areas and countries, with an 95% uncertainty interval (UI) determined for each parameter. To calculate the 95%UI, the input parameters were presumed to be normally distributed.^14^ Specifically, 95% UI is typically obtained through Monte-Carlo simulation with 10 000 trials, where all inputs change simultaneously.^15^ The age-standardized rate (ASR) per 100 000 population was used as the population size-based rate, and estimated annual percentage changes (EAPCs) were used to describe the change tendencies in ASRs over a given period. EAPC and ASR are both used to evaluate the trend of disease burden changes. When regarding potential differences in age constitution over time among multiple populations, ASR is a typical indicator. ASR is computed according to the following formula:


$$ASR=\frac{\sum_{i=1}^{A}a_{i}w_{i}}{\sum_{i=1}^{A}w_{i}}\times 100,000$$

Where *a*_i_ is the age-specific rate in the *i*^th^ age categorize, *w* is the number of people in the commensurate *i*^th^ age group among the adopted reference criterion population, and *A* is the number of age groups.

 EAPC is a widely accepted metric for describing the magnitude of ASR trends.^16,17^


$$\begin{array}{c} y=\alpha +\beta t+\varepsilon \\ EPAC=\left(\frac{\hat{{\text{ASR}}_{\text{t}+1}-\hat{{\text{ASR}}_{\text{t}}}}}{\hat{{\text{ASR}}_{\text{t}}}}\right)\cdot 100\\ =\left(\frac{{\hat{\text{ASR}}}_{\text{t}+1}}{{\hat{\text{ASR}}}_{\text{t}}}-1\right)\cdot 100=\left(\frac{e^{\hat{y}+\hat{\beta }}\left(t+1\right)}{e^{\hat{y}+\hat{\beta }}\cdot t}-1\right)\cdot 100\\ =\left(exp\left(\beta \right)-1\right)\times 100 \end{array}$$

Where y = ln (ASR) and t = calendar year. Both the EAPC value and its 95% CI > 0 illustrated a rising trend, and both the EAPC value and its 95% CI < 0 illustrated a declining trend. Other values illustrated that the trend was stable with the passage of time. The Joint Region Program 4.7.0.0 software (American Cancer Research Institute) was used to appraise the EAPCs and 95% confidence intervals (CIs) of age-standardized death rates (ASDRs) and age-standardized DALY rates for TBL cancer attributable to HFPG between 1990 and 2019. The software can automatically identify change points based on the actual trend of data changes. This software determines the best model fit by gradually examining different numbers of change points. Secondly, the number of change points is determined through statistical significance testing. For each additional change point, hypothesis testing is performed to examine whether the new change point significantly improves the quality of the model fit. The regression analysis of disease burden was performed using a joint regression model. The response variables of the joint regression model in this study are age standardized mortality rate (ASDR) and age standardized DALY rate, which are utilized to evaluate the burden of TBL cancer caused by HFPG. In the joint regression model, the error term (𝜖_t_) is assumed to be normally distributed, meaning that the error term at each time point is standalone and identically distributed, and complies a normal distribution with a mean of 0 and a variance of σ^2^. This assumption is consistent with the assumptions in standard regression analysis, meaning that the bias of each data point is random and does not exhibit systematic patterns over time or other variables. In this study, to adapt to the characteristics of proportional data such as mortality rate (ASDR) and disability adjusted life year (DALY) rates, a log link function was used. This method solves the problem of possible skewed distribution and heteroscedasticity in the data by performing logarithmic transformation on the response variables in the joint regression model, ensuring that the regression analysis results are more reliable. The progressive increase in the burden was indicated by the lower bounds of the EAPC and its 95% CI being greater than 0, whereas a downward trend in the burden was indicated by the upper bounds of the EAPC and its 95% CI being less than 0. Furthermore, ASDRs and age-standardized DALY rates were utilized to evaluate the distribution of the TBL cancer burden attributable to HFPG in different areas from 1990 to 2019. In addition, EAPCs in ASDRs and DALYs for TBL cancer attributable to HFPG were used to evaluate the change tendencies over the entire period with respect to SDIs in 21 regions and 204 countries.

 To correct for the presence of heteroscedasticity, White heteroscedasticity consistent standard error and covariance were used to ensure the accuracy and stability of the regression results.^18^

 For autocorrelation, considering the time series nature of the data, we appropriately used autoregressive integrated moving average (ARIMA) interrupted time series assessment in statistical analysis to diagnose and test the autocorrelation of the results.^19^

 In summary, we took into account heteroscedasticity and autocorrelation during the analysis process and made diagnoses and adjustments as appropriate.

## Results

###  Global Level

 Globally, in 2019, the numbers of TBL cancer deaths and DALYs attributable to HFPG were 179,048.78 (95% UI: 42 684.98–389 384.23) and 3 640 548.35 (95% UI: 856 167.14–8 012 528.62), respectively. In addition, compared with 1990, the global numbers of TBL cancer deaths and DALYs attributable to HFPG in 2019 were 166.19% and 141.02% greater, respectively, with male deaths and DALYs being 124 102.19 and 2 569 121.65, respectively, and female deaths and DALYs being 54 946.59 and 1 071 426.70, respectively. Similarly, in 2019, the ASDR (2.22, 95% UI: 0.53–4.83) and age-standardized DALY rate (44.06, 95% UI: 10.42–96.82) for TBL cancer attributable to HFPG per 100 000 population were significantly higher than the corresponding values in 1990 (ASDR: 1.78, 95% UI: 0.39–3.99; age-standardized DALY rate: 37.84, 95% UI: 8.10–85.68).

**Figure 1:**
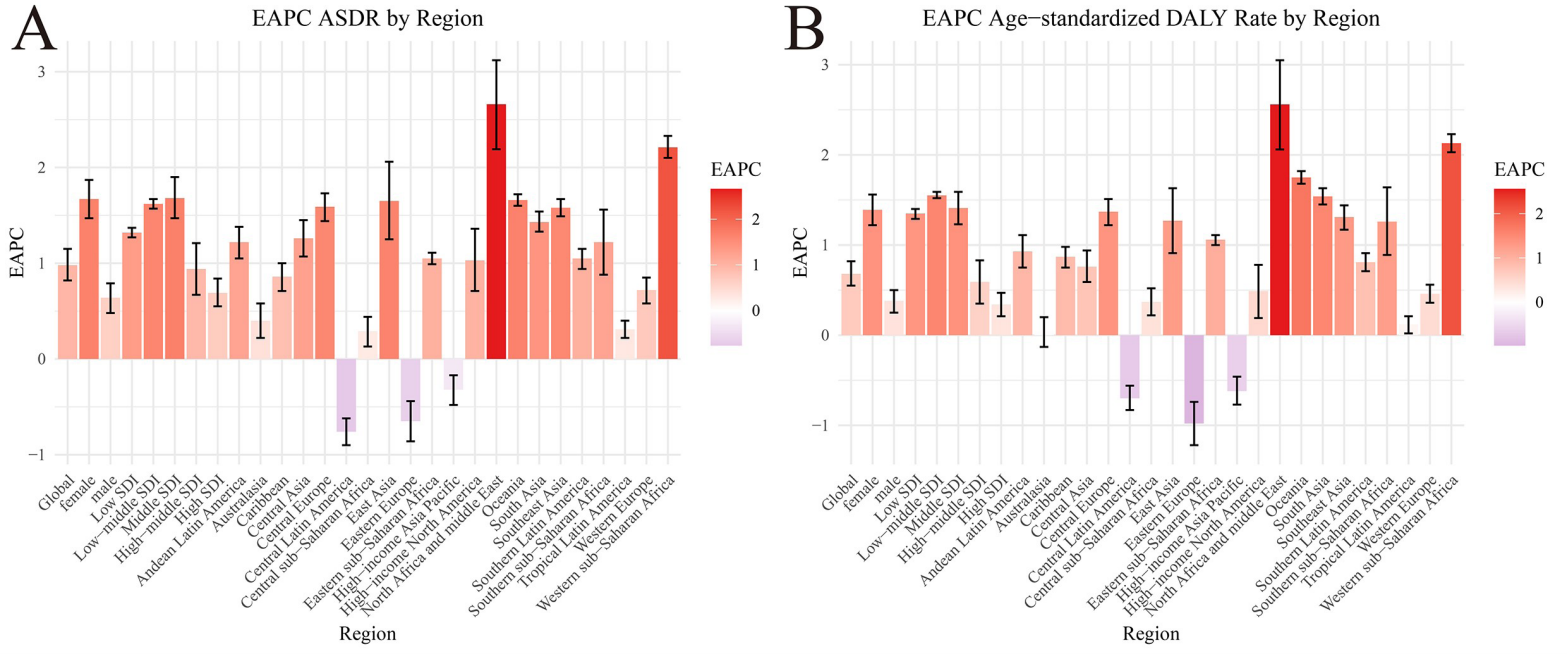
EAPC of ASRs in TBL cancer attributed to HFPG from 1990 to 2019, by region. **(A)** EAPC of ASDR. **(B)** EAPC of age-standardized DALY rate. EAPC = estimated annual percentage change. ASRs = age-standardized rates. ASDR = Age standardized death rate. DALY = Disability adjusted life-year

 The ASDR (EAPC = 0.98; 95% CI: 0.82–1.15; Table S1 and Figure 1A) and age-standardized DALY rate (EAPC = 0.68; 95% CI: 0.55–0.82; Table S1 and Figure 1B) of TBL cancer attributable to HFPG from 1990 to 2019 showed an upward trend. Thus, the global burden of TBL cancer attributable to HFPG increased.

###  Regional Level

**Figure 2:**
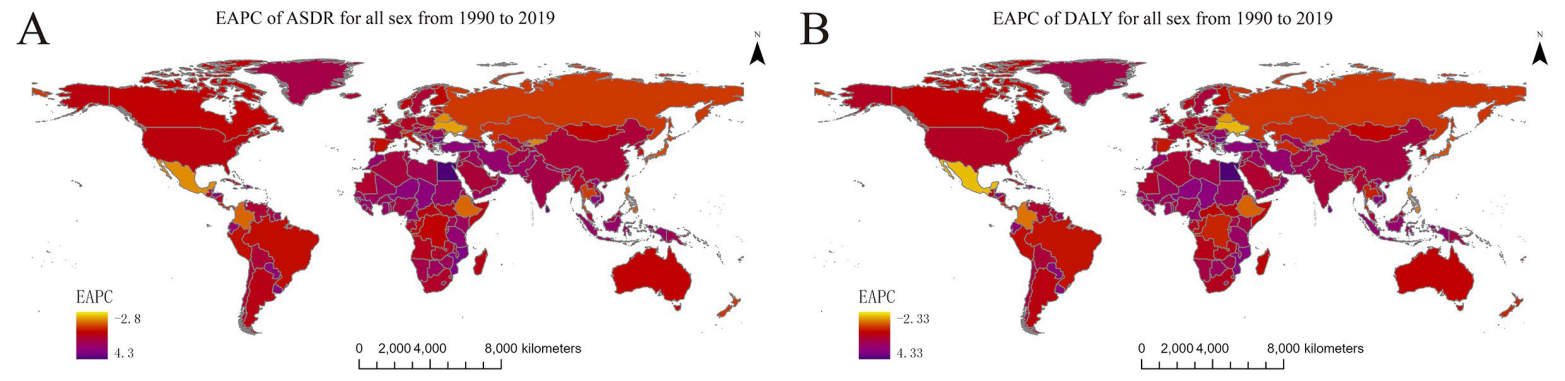
EAPC of Global ASRs of TBL Cancer Attributed to HFPG for Both Genders in 204 Countries from 1990 to 2019. **(A)** EAPC of ASDR. **(B)** EAPC of age-standardized DALY rate. EAPC = estimated annual percentage change. ASRs = Age-standardized rates. ASDR = Age standardized death rate. DALY = Disability adjusted life-year

**Figure 3:**
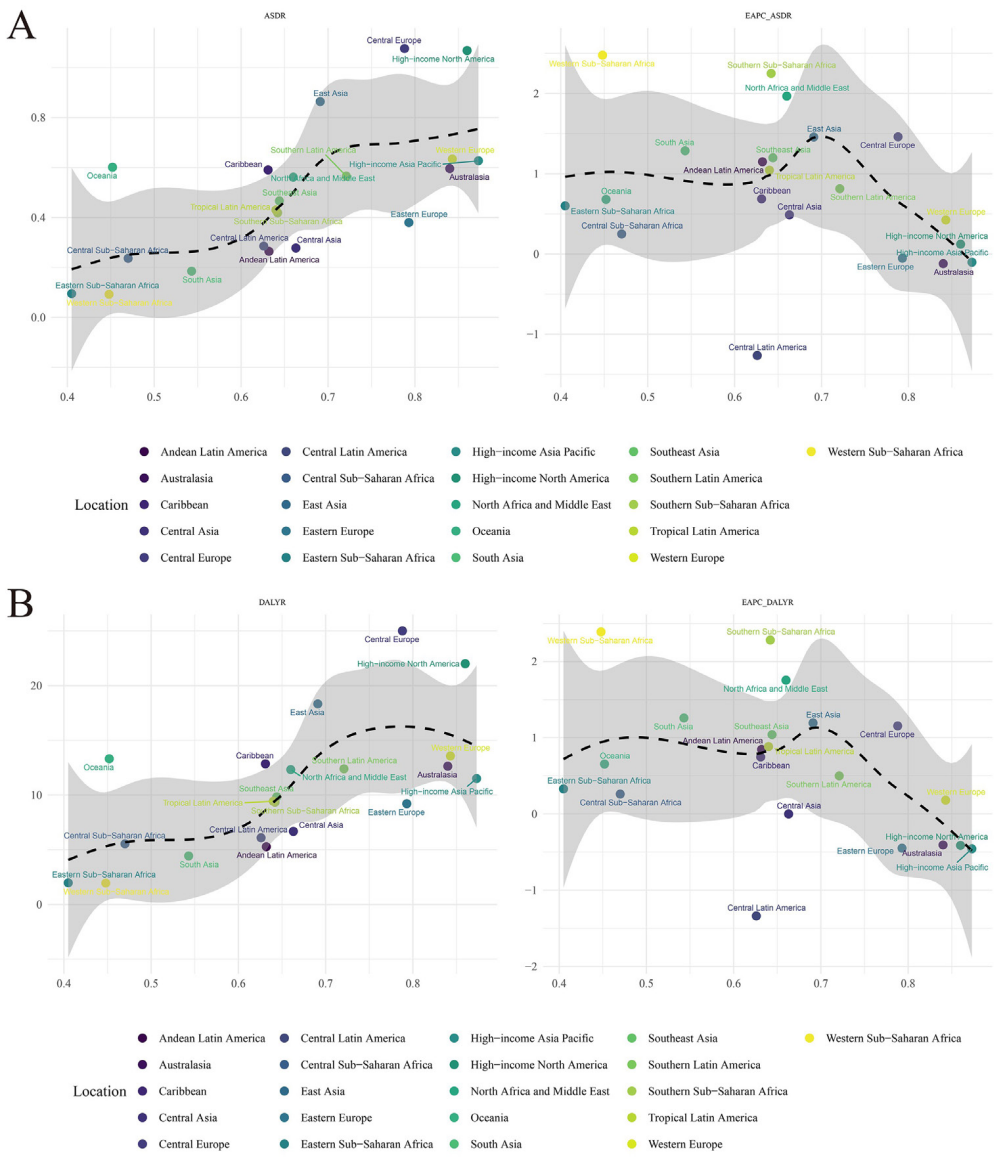
Relationship between Global TBL Cancer Attributed to HFPG in Distinct Regions and SDI from 1990 to 2019 **(A)** ASDR and EAPC to SDI **(B)** age-standardized DALY rate and EAPC to SDI. EAPC = Estimated annual percentage change. ASDR = Age standardized death rate. DALY = Disability adjusted life-year

 In 2019, among 21 geographical regions worldwide, the three areas with the highest ASDRs for TBL cancer attributable to HFPG per 100 000 population were High-income North America (4.54, 95% UI: 1.12–9.46), Central Europe (3.83, 95% UI: 0.83–8.39), and Oceania (3.10, 95% UI: 0.66–7.06), whereas the three areas with the lowest ASDRs were Eastern sub-Saharan Africa (0.49, 95% UI: 0.10–1.12), Western sub-Saharan Africa (0.72, 95% UI: 0.16–1.65), and South Asia (0.86, 95% UI: 0.19,1.92). From 1990 to 2019, the ASDR for TBL cancer attributable to HFPG increased the most in North Africa and Middle East (EAPC = 2.66, 95% CI: 2.19–3.12), followed by Western sub-Saharan Africa (EAPC = 2.21, 95% CI: 2.10–2.33) and Oceania (EAPC = 1.66, 95% CI: 1.60–1.72), whereas it decreased the largest in Central Latin America (EAPC = -0.76, 95% CI: -0.90 to -0.62), followed by Eastern Europe (EAPC = -0.65, 95% CI: -0.86 to -0.44) and High-income Asia Pacific (EAPC = -0.32, 95% CI: -0.48 to -0.17) (Table S1, Figure 2A, Figure 3A).

 In 2019, the region with the highest age-standardized DALY rate for TBL cancer attributable to HFPG per 100 000 population was High-income North America (89.6, 95% UI: 122.07–187.33), followed by Central Europe (86.42, 95% UI: 18.46–190.56) and Oceania (68.29, 95% UI: 14.06–158.89), while the region with the lowest age-standardized DALY rate was Eastern sub-Saharan Africa (9.68, 95% UI: 2.00–22.25), followed by Western sub-Saharan Africa (13.65, 95% UI: 2.87–31.48) and Andean Latin America (17.55, 95% UI: 4.05–40.25). From 1990 to 2019, the age-standardized DALY rate for TBL cancer attributable to HFPG increased the largest in North Africa and Middle East (EAPC = 2.56, 95% CI: 2.06–3.05), followed by Western sub-Saharan Africa (EAPC = 2.13, 95% CI: 2.03–2.23) and Oceania (EAPC = 1.75, 95% CI: 1.68–1.82), whereas it decreased the largest in Eastern Europe (EAPC = -0.98, 95% CI: -1.22 to -0.74), followed by Central Latin America (EAPC = -0.70, 95% CI: -0.83 to -0.56) and High-income Asia Pacific (EAPC = -0.62, 95% CI: -0.77 to -0.46) (Table S1, Figure 2B, Figure 3B).

###  National Level

**Figure 4:**
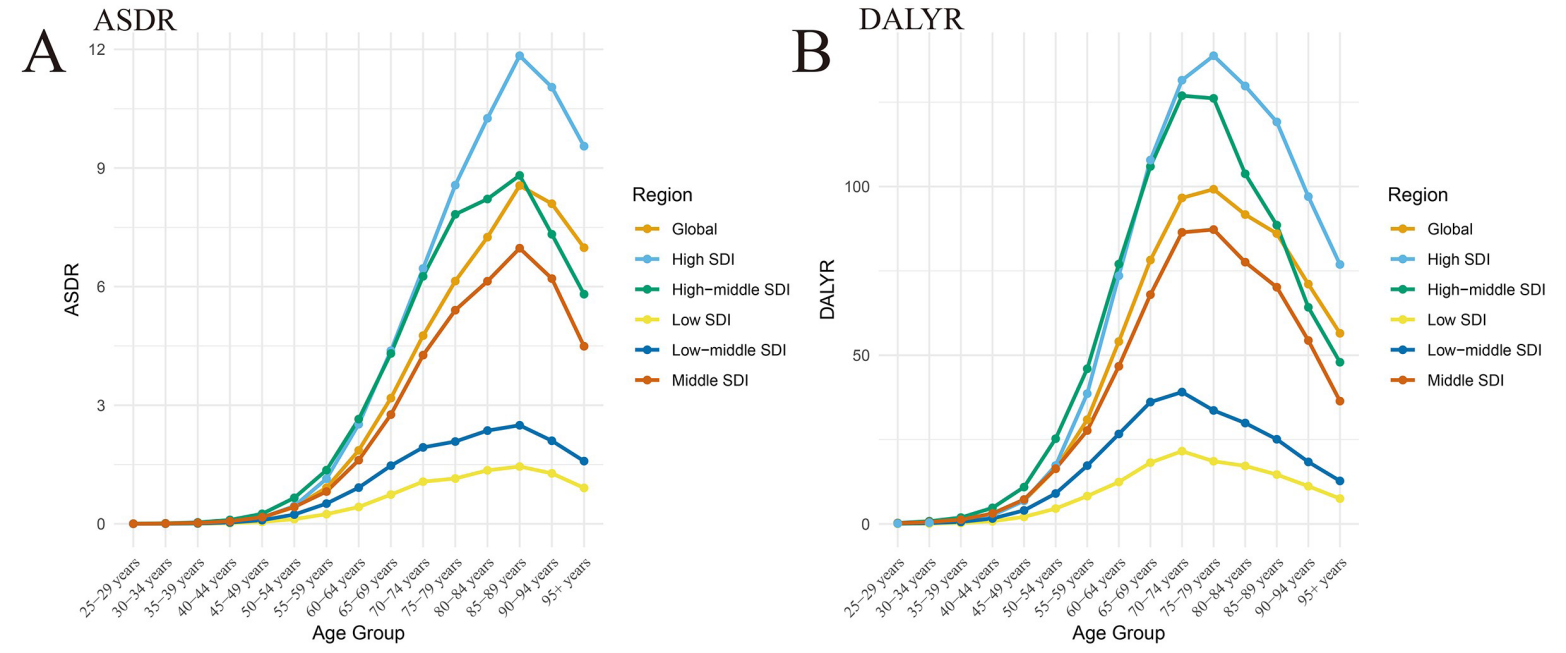
TBL Cancer Burden Attributed to HFPG from 1990 to 2019, by Region and Age. **(A)** The Death rate. **(B)** The DALY rates. DALY = disability adjusted life-year

 In 2019, among the 204 countries, the ASDRs for TBL cancer attributable to HFPG per 100 000 population were the highest in Brunei Darussalam (8.77, 95% UI: 2.39–17.42), followed by Palau (7.30, 95% UI: 1.82–15.48) and the Marshall Islands (6.56, 95% UI: 1.48–15.94), whereas they were the lowest in Ethiopia (0.34, 95% UI: 0.06–0.85), followed by Somalia (0.37, 95% UI: 0.07–1.06) and Kenya (0.40, 95% UI: 0.07–1.06). From 1990 to 2019, the ASDRs per 100 000 population for TBL cancers attributable to HFPG increased the most in Georgia (EAPC = 4.33, 95% CI: 3.66–5.00), followed by Monaco (EAPC = 3.83, 95% CI: 3.34–4.34) and Egypt (EAPC = 4.27, 95% CI: 4.14–4.40), whereas they decreased the most in Singapore (EAPC = -2.33, 95% CI: -2.53 to -2.12), followed by Mexico (EAPC = -1.79, 95% CI: -2.09 to -1.49) and Ukraine (EAPC = -1.73, 95% CI: -2.07 to -1.38) (Table S2, Figure 2A, Figure 4).

 In 2019, the countries with the highest age-standardized DALY rates for TBL cancer attributable to HFPG per 100 000 population were Brunei Darussalam (159.28, 95% UI: 43.13–319.75), Palau (159.16, 95% UI: 39.94–343.92), and Marshall Islands (144.79, 95% UI: 31.98–36.43), and those with the lowest age-standardized DALY rates were Ethiopia (6.39, 95% UI: 1.21–15.98), Kenya (7.99, 95% UI: 1.58–18.80), and Madagascar (8.16, 95% UI: 1.60–20.07). From 1990 to 2019, the age-standardized DALY rate increased the most in Egypt (EAPC = 4.34, 95% CI: 4.22–4.47), followed by Georgia (EAPC = 4.16, 95% CI: 3.49–4.84) and Sri Lanka (EAPC = 3.69, 95% CI: 3.36–4.02), while it decreased the most in Singapore (EAPC = -2.78, 95% CI: -2.97 to -2.59), followed by Bahrain (EAPC = -2.05, 95% CI: -2.51 to -1.58) and Ukraine (EAPC = -1.88, 95% CI: -2.27 to -1.49) (Table S3, Figure 2B, Figure 4).

###  Gender and Age Distribution Trends

 The occurrence of TBL cancer attributable to HFPG varied between across and age groups. In 2019, the male ASDR (3.42, 95% UI: 0.58–7.88) and age-standardized DALY rate (66.58, 95% UI: 11.24–153.36) were higher than the female ASDR (1.25, 95% UI: 0.25–2.93) and age-standardized DALY rate (24.42, 95% UI: 4.82–58.24) for TBL cancer attributable to HFPG per 100 000 population. From 1990 to 2019, the ASDRs and age-standardized DALY rates for TBL cancer attributable to HFPG increased in both males and females (ASDR: male EAPC = -3.81, female EAPC = -3.52; age-standardized DALY rate: male EAPC = -3.68, female EAPC = -3.53), with the rates being lower in males than females (Table S1).

**Figure 5:**
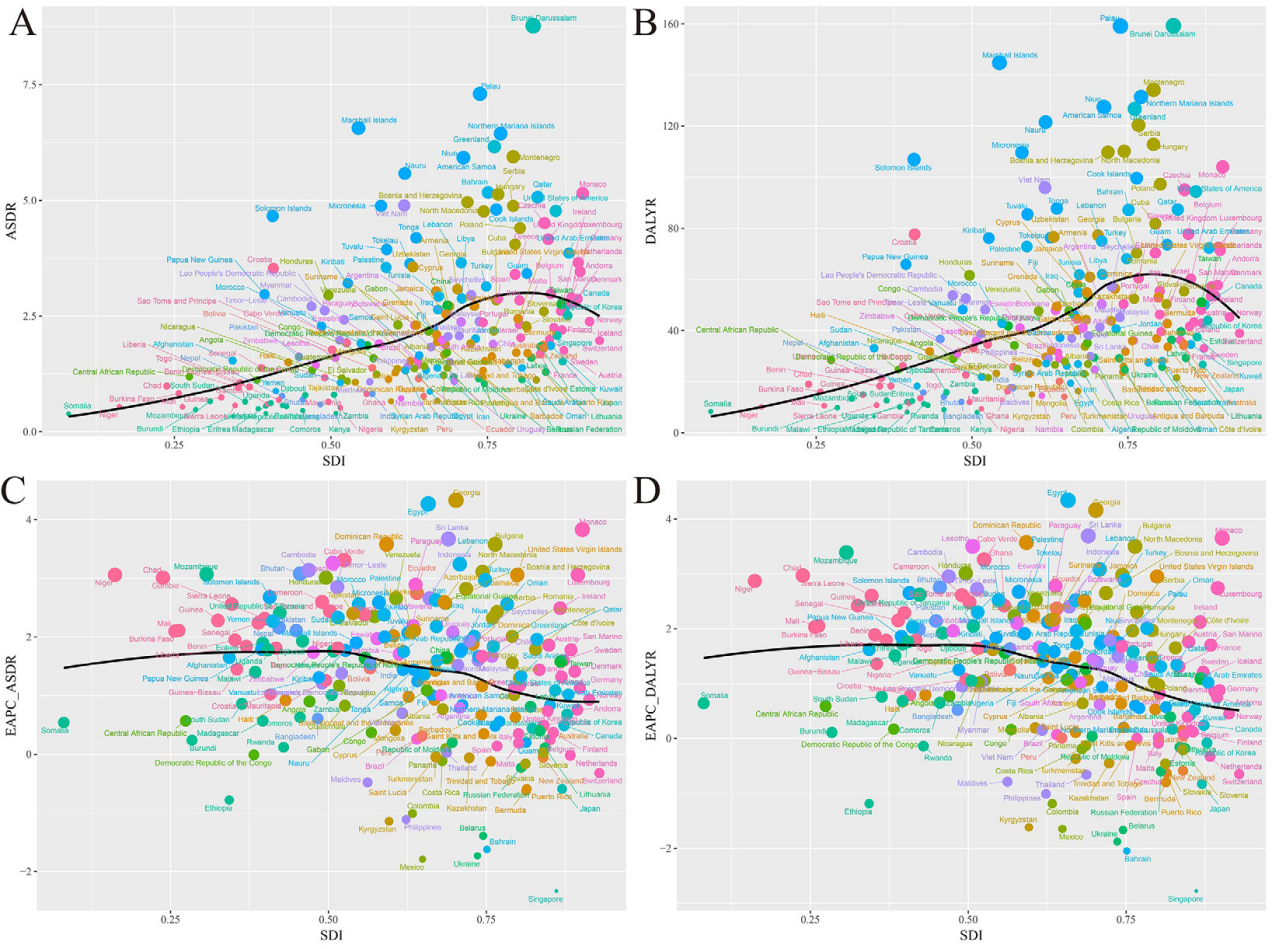
Global TBL Cancer Attributed to HFPG in Different Regions by SDI **(A)** ASDR in 2019 **(B)** Age-Standardized DALY rate in 2019 **(C)** EAPC of ASDR from 1990 to 2019 **(D)** EAPC of Age-Standardized DALY Rate from 1990 to 2019. ASDR = Age standardized death rate. EAPC = Estimated annual percentage change. DALY = Disability adjusted life-year

 In 2019, the ASDRs and standardized DALY rates for TBL cancer attributable to HFPG in all SDI regions first increased and then declined with age. The highest ASDRs in all, middle-, middle-high, and high-SDI districts occurred in those aged 85–89, whereas the highest ASDRs in low- and middle-SDI regions occurred in those aged 80–84. Similarly, the largest age-standardized DALY rates globally and in various SDI areas occurred in those aged 70–74 (Figure 5). Among the 21 geographical regions, the incidence rate of TBL cancer attributable to HFPG was significantly higher in high-SDI countries and districts, comprising Australia and High-income North America. In addition, the incidence rate was highest in those aged 60–84.

###  SDI-Region Distribution of Burden and Change Trends

 In 2019, the highest ASDR and age-standardized DALY rates for TBL cancer attributable to HFPG per 100 000 population occurred in high-SDI regions. In addition, the ASDR (3.11, 95% UI: 0.76–6.60) and age-standardized DALY rates (61.28, 95% UI: 14.74–131.02) in high-SDI regions, and the ASDR (2.41, 95% UI: 0.55–5.32) and age-standardized DALY rate (49.47, 95% UI: 10.88–110.11) in middle-high-SDI districts, were significantly greater than those in other SDI regions. Low-SDI regions had the lowest ASDR (0.79, 95% UI: 0.17–1.81) and age-standardized DALY rate (16.64, 95% UI: 3.48–38.41). The ASDRs and age-standardized DALY rates consistently trended upward in the five SDI regions and increased the most in low-SDI regions (EAPC = 1.32; EAPC = 1.35). As the SDI increased, the magnitude of the increase in ASDRs and age-standardized DALY rates gradually decreased, i.e. high-SDI regions < middle-high-SDI regions < low-middle-SDI regions < low-SDI regions (Table S1, Figure 1).

 As shown in Figure 3A, a positive interrelationship emerged linking SDIs with HFPG-related TBL cancer ASDRs across 21 regions. Regions with high SDIs had high ASDRs, such as High-income North America, Central Europe, and East Asia, with these ASDRs being much higher than expected. The EAPCs in the ASDR of TBL cancer attributable to HFPG exhibited a varying but overall downward trend: regions with SDIs of roughly 0.6 usually had high EAPCs, and North Africa, Middle East and Western sub-Saharan Africa had significantly higher growth rates than expected (as shown in Figure 3B).

 Among 204 countries, the relationship between ASDRs of TBL cancer attributable to HFPG and SDIs first increased and then decreased (Figure 4A), with ASDRs being highest in countries with SDIs of approximately 0.75, such as Palau, Northern Mariana Islands, Montenegro, and Greenland. The relationship between ASDRs, EAPCs, and SDI exhibited a similar trend (Figure 4C). However, the relationship between DALYs of TBL cancer attributable to HFPG and SDIs first decreased and then increased (Figure 4B). In countries with SDIs less than 0.5, there tended to be no relationship between SDIs and ASDRs. However, as countries’ SDIs increased beyond 0.5, their ASDRs first almost invariably decreased but then increased as SDIs reached approximately 0.8. In particular, the ASDRs in Egypt, Cabo Verde, Monaco, and other countries were far higher than expected. The interrelation between DALYs, EAPCs, and SDI also exhibited an analogous trend (Figure 5D).

## Discussion

 In this study, a review of GBD 2019 data showed that, from 1990 to 2019, there were temporal and regional differences in the burden of TBL cancer attributable to HFPG. The global burden of TBL cancer attributable to HFPG trended upward overall between 1990 and 2019, especially in men and high-SDI areas. The increases in the mortality rate and total DALYs due to TBL cancer attributable to HFPG may be a result of economic development, population growth, and accelerated aging over the prior decade. However, the increase in ASDRs and age-standardized DALY rates cannot be ascribed entirely to demographic factors; it is also can be attributed to various factors, including elevated body mass index, an unwholesome diet, and inadequate physical activity, all of which increase the risk of TBL cancer attributable to HFPG. Moreover, over the prior three decades, the number of adults who have reached the age of 18 and have diabetes has almost doubled. Thus, hyperglycemia and diabetes are increasingly serious global health problems,^2^ and TBL cancer attributable to HFPG is a large burden on healthcare systems worldwide.

 The key risk factors for TBL cancer include smoking, environmental pollution, HFPG, and an unhealthy diet (i.e. low intake of fruits and vegetables).^14^ HFPG shows a relationship with overweight, an unhealthy diet, low physical activity, air pollution, smoking, and other factors, as has been comprehensively determined in systematic reviews of multiple cohort studies.^15,16^ The most recent epidemiological study performed by the American Diabetes Association and the World Health Organization found that FPG is closely related to daily cigarette consumption and cumulative annual packaging exposure events. Aside from obesity, smokers have a higher relative risk of HFPG and type 2 diabetes.^13^ In addition, exposure to general air pollutants (2.5-µm particulate matter [PM_2.5_], PM_10_, nitrogen dioxide, ozone, and sulfur dioxide) is positively correlated with changes in FPG.^17^ Exposure to such pollutants increases oxidative stress and lung inflammation, which negatively affects the regulation of glucose metabolism by interrupting the insulin signaling pathway, leading to abnormal increases in glucose.^18^ Continuously increasing glucose can cause toxicity and have adverse pathological effects on multiple organs, such as the lungs and heart.^5^ Exposure to air pollution is thus a risk factor for HFPG and type 2 diabetes in pre-diabetic patients. Diet is also a key factor affecting blood glucose, and dietary management can reduce blood glucose. A high-calorie diet can contribute to elevated blood glucose and a higher incidence rate of type 2 diabetes.^19^ Therefore, HFPG directly and indirectly affects the occurrence, development, and prognosis of TBL cancer.

 The socioeconomic status of a nation has a positive relationship with the incidence of TBL cancer.^20^ The current study found that from 1990 to 2019, the burden of TBL cancer attributable to HFPG rose as the SDIs increased, whereas the growth rate related to EAPCs fluctuated. The high burdens of TBL cancer attributable to HFPG in high-SDI countries or regions are due to mechanization of production (as this has resulted in sedentary work gradually replacing physically intensive agricultural work) and increases in intake of high-sugar, red-meat, and other fattening food. In other words, the aforementioned changes have led to increases in FPG and the prevalence of diabetes, thereby increasing the incidence rate of TBL cancer. However, in high-SDI countries or regions, advances in screening technology and medicine mean that TBL cancer patients are increasingly likely to be diagnosed and receive timely care and treatments. Thus, the increase in the incidence rate of TBL cancer in high-SDI countries or regions is not as substantial as that in lower-SDI countries or regions.^21^ For example, the economies of High-income North America and Singapore are highly developed, and their populations are aging while life expectancy is increasing. Moreover, in the United States and Canada, more processed food is sold per capita than in any other country, and the dietary pattern comprising excessive intake of harmful substances and low intakes of grain and fruit is increasing in prevalence, as people are consuming higher proportions of high-sugar drinks, red meat, and processed meat than in previous years. This contributes to rising obesity rates and elevated prevalence of pre-diabetes and diabetes. For example, research by the National University of Singapore shows that, Singaporeans have a high risk of diabetes in one third of their lifetime (based on 30-year cohort data), and their daily intake of sugar is more than twice the amount recommended by the World Health Organization. Consequently, 14% of Singaporeans aged 18–19 were diagnosed as pre-diabetic, that is, having a blood sugar higher than normal,^22^ and it is estimated that in 2050, more than 1 million Singaporeans will suffer from diabetes. However, Singapore “declared war” on diabetes in 2016 and has successively launched a variety of measures, such as the “National Walking Challenge,” the “Healthy Food Material Development Plan,” the “Comprehensive Prohibition of High-sugar Beverage Advertising,” and the “No smoking Policy”.^23^ In addition, the National Health Care Group has established an enterprise-wide diabetes registration system,^24^ which has greatly reduced the disease burden attributable to diabetes while providing quality care.

 The burden of TBL cancer attributable to HFPG in North Africa and Middle East, Georgia, and Egypt is due to high body mass index, lack of exercise, and environmental pollution.^25^ In addition, early nutritional deficiency may lead to insulin resistance and elevate the risk of hyperglycemia and type 2 diabetes in adulthood.^26^ Economic growth is accompanied by environmental pollution, which is also related to an increased risk of TBL cancer.^27^ In Brunei Darussalam, lung cancer is the leading contributor to mortality and constitutes 24.9% of the incidence rate of all cancers.^28^ The predominant proportion of lung cancers are attributable to smoking.^29^ Moreover, the prevalence of obesity in Brunei Darussalam is 28%, and diabetes is the third leading cause of death in the country. A high prevalence of diabetes and obesity means that cancer patients with HFPG lack valid management, lack awareness of blood sugar control, have low self-management compliance, and are prone to developing hyperglycemia, which makes the course of TBL cancer complex and leads to continuous disability and ultimately death. The effective blood-sugar control rate in low- and middle-income countries is only 23.0%, far lower than that in the United States, where it is 52.3%.^30^ Furthermore, in low-SDI regions, such as Latin America, Central Africa, and Eastern Europe, limitations in equipment and cancer-registration systems, coupled with insufficient awareness of HFPG, mean that the actual TBL cancer burden attributable to the HFPG risk factor is likely to be more than the evaluated burden.^31^

 The current study indicated that men were more likely than women to have TBL cancer, which is consistent with previous studies.^32^ This may be due to male sex hormones, which cause men to have more visceral fat than women and thus more insulin resistance than women.^33^ Consequently, males are substantially more likely than females to have diabetes, both in terms of prevalence and growth rate.^34^ In addition, compared with women, men exhibit a higher propensity for smoking and hypertension, dyslipidemia, and other risk factors.^35^ However, from 1990 to 2019, there was a greater increase in the TBL cancer burden in women than in men, which may be due to hormonal factors. For example, it was found that exposure to combined hormone therapy (estrogen-progestogen) may confer a 50% increased risk of lung cancer among women, and perturbations in hormonal homeostasis could similarly predispose to TBL cancer.^36^ It was also found that women who smoke are more likely than men who smoke to have TBL cancer,^37^ and smoking ranks first among all of the causes of TBL cancer in women. Smoking is also becoming increasingly popular, which may be the main cause for the increase in the TBL cancer burden in women. Exposure to secondhand and environmental pollutants, particularly indoor airborne contaminants, contributes significantly to the disease burden of TBL cancer.

 The current study also found that people aged 60–89 had the highest burden of TBL cancer. Physical health declines with age, meaning that compared with younger people, older adults are more likely to suffer from diabetes and have long-lasting hyperglycemia and more complications.^38^ Therefore, primary healthcare is pivotal for suppressing the increasing burden of TBL cancer in men and older adults.^39^ Moreover, there has been a greater increase in TBL cancer attributable to HFPG in women than in men, and this burden cannot be ignored.^40^

 This research examined the newest data from GBD 2019 to determine the burden of TBL cancer attributable to HFPG. It also examined the distribution characteristics and change trends of this burden from 1999 to 2019 at the global, national, and regional levels and in terms of gender, age, and SDIs. However, this study also has several limitations that should be acknowledged. First, it was difficult to distinguish the independent effect of HFPG on the burden of TBL cancer because it is usually a contributing factor and diabetes, chronic kidney disease, and ischemic heart disease are common complications.^41^ Thus, it was sometimes difficult to eliminate the impact of diabetes on the burden of TBL cancer. Second, the data did not distinguish between tracheal cancer, bronchial cancer, and lung cancer.^42^ Third, the generalizability of our findings is restricted to individuals aged 25 years or order, i.e., it does not apply to children and adolescents. Fourth, the results do not include cancer burdens at subnational levels, which must be examined in future research to assist decision-makers.^43^

 From 1990 to 2019, the global burden of TBL cancer attributable to HFPG exhibited an overall ascending trend, particularly in low-SDI regions and in countries with the largest growth rates, for instance North Africa and Middle East, Georgia, and Egypt. Our results indicate that good-quality primary healthcare is especially important for improving the TBL cancer burden in men and older adults. Significant progress in the control of TBL cancer has been made in high-SDI regions and countries, such as High-income North America and Brunei Darussalam. However, proactive targeted behavioral interventions remain a strategic focus for mitigating the worldwide burden of TBL cancer attributable to HFPG.

## Conclusion

 The global burden of TBL cancer attributable to HFPG trended upward from 1990 to 2019. The largest increase in the burden occurred in low-SDI regions and countries, for instance North Africa and Middle East, Georgia, and Egypt. Geographic regions and countries bearing the highest disease burden, for instance High-income North America and Brunei Darussalam, and especially low-SDI regions, should improve primary healthcare to control fasting plasma glucose.

## Supplementary files

Supplementary file 1 contains Tables S1-S3.
