# Supplementary material for: Burden of Tracheal, Bronchus, and Lung Cancer Attributable to High Fasting Plasma Glucose
Source: Arch Iran Med. 2025 May 1;28(5):286–95. doi: 10.34172/aim.33332 (PMC12305407; doi:10.34172/aim.33332)
Supplement: Supplementary file 1 — contains Tables S1-S3. [file aim-28-286-s001.pdf]

Arch Iran Med. May 2025;28(5):286-S1
Doi: 10.34172/aim.33332
http://journalaim.com

Supplementary file 1

Table S1. Age-standardized death rate (ASDR) and Age-standardized DALY of TBL cancer attributed to HFPG in 1990 and 2019 and its temporal trends

Characteristics	1990		2019		EAPC (1990-2019)	
	ASDR per 10000 No.(95%UI)	Age-standardized DALY rate per 10000 No.(95%UI)	ASDR per 10000 No.(95%UI)	Age-standardized DALY rate per 10000 No.(95%UI)	ASDR No.(95%CI)	Age-standardized DALY No.(95%CI)
Global	1.78(0.39,3.99)	37.84(8.10,85.68)	2.22(0.53,4.83)	44.06(10.42,96.82)	0.98(0.82,1.15)	0.68(0.55,0.82)
Sex	—	—	—	—	—	—
female	0.83(0.16,1.98)	17.35(3.21,41.45)	1.25(0.25,2.93)	24.42(4.82,58.24)	1.67(1.47,1.87)	1.39(1.22,1.56)
male	3.02(0.49,7.16)	62.41(10.14,149.22)	3.42(0.58,7.88)	66.58(11.24,153.36)	0.64(0.48,0.79)	0.38(0.25,0.50)
Sociodemographic index	—	—	—	—	—	—
Low SDI	0.54(0.10,1.33)	11.13(2.05,27.76)	0.79(0.17,1.81)	16.64(3.48,38.41)	1.32(1.27,1.37)	1.35(1.29,1.40)
Low-middle SDI	0.71(0.15,1.65)	15.12(3.04,35.22)	1.17(0.27,2.61)	24.57(5.43,55.45)	1.62(1.57,1.67)	1.55(1.52,1.59)
Middle SDI	1.35(0.30,3.08)	28.78(6.26,66.52)	2.10(0.48,4.68)	41.82(9.52,94.98)	1.68(1.47,1.90)	1.41(1.23,1.59)
High-middle SDI	1.92(0.40,4.40)	43.34(8.77,100.46)	2.41(0.55,5.32)	49.47(10.88,110.18)	0.94(0.67,1.21)	0.59(0.35,0.83)
High SDI	2.76(0.62,6.07)	59.41(13.15,131.41)	3.11(0.76,6.60)	61.28(14.74,131.02)	0.69(0.55,0.84)	0.34(0.21,0.47)
Region	—	—	—	—	—	—
Andean Latin America	0.69(0.15,1.59)	13.48(2.82,31.18)	0.96(0.22,2.18)	17.55(4.05,40.25)	1.22(1.05,1.38)	0.93(0.75,1.11)
Australasia	1.59(0.33,3.61)	31.23(6.51,71.62)	1.86(0.43,4.09)	34.23(7.77,76.17)	0.40(0.22,0.58)	0.04(-0.13,0.20)
Caribbean	2.10(0.47,4.60)	41.69(9.00,92.90)	2.55(0.59,5.52)	51.45(11.59,112.76)	0.86(0.71,1.00)	0.87(0.75,0.98)
Central Asia	1.22(0.25,2.81)	31.17(6.16,72.93)	1.61(0.35,3.56)	36.74(7.77,82.13)	1.26(1.07,1.45)	0.76(0.59,0.94)
Central Europe	2.53(0.50,5.72)	60.55(11.57,138.85)	3.83(0.83,8.39)	86.42(18.46,190.56)	1.59(1.44,1.73)	1.37(1.22,1.51)

Central Latin America	1.73(0.43,3.68)	34.12(8.31,73.36)	1.53(0.37,3.29)	30.44(7.36,66.39)	-0.76(-0.90,-0.62)	-0.70(-0.83,-0.56)
Central sub-Saharan Africa	1.09(0.18,3.60)	23.42(3.68,77.67)	1.22(0.23,3.46)	26.76(4.83,77.61)	0.29(0.13,0.44)	0.37(0.22,0.52)
East Asia	1.83(0.41,4.26)	39.65(8.48,92.83)	2.72(0.61,6.19)	53.54(11.80,122.53)	1.65(1.25,2.06)	1.27(0.91,1.63)
Eastern Europe	1.20(0.23,2.83)	30.06(5.68,71.63)	1.14(0.23,2.68)	26.75(5.32,63.55)	-0.65(-0.86,-0.44)	-0.98(-1.22,-0.74)
Eastern sub-Saharan Africa	0.36(0.07,0.87)	7.16(1.36,17.65)	0.49(0.10,1.12)	9.68(2.00,22.25)	1.05(0.99,1.11)	1.06(1.00,1.11)
High-income Asia Pacific	1.68(0.35,3.79)	32.46(6.67,73.64)	1.72(0.36,3.87)	30.61(6.20,69.45)	-0.32(-0.48,-0.17)	-0.62(-0.77,-0.46)
High-income North America	4.12(0.94,8.97)	92.78(21.06,203.57)	4.54(1.12,9.46)	89.61(22.07,187.33)	1.03(0.71,1.36)	0.49(0.19,0.78)
North Africa and middle East	1.15(0.22,2.74)	25.00(4.64,60.82)	1.91(0.41,4.17)	39.91(8.58,88.56)	2.66(2.19,3.12)	2.56(2.06,3.05)
Oceania	1.94(0.40,4.69)	41.67(8.48,101.08)	3.10(0.66,7.06)	68.29(14.06,158.89)	1.66(1.60,1.72)	1.75(1.68,1.82)
South Asia	0.52(0.10,1.25)	11.12(2.10,26.57)	0.86(0.19,1.92)	18.57(3.98,42.05)	1.43(1.33,1.54)	1.54(1.45,1.63)
Southeast Asia	1.19(0.25,2.72)	24.62(5.15,57.43)	1.99(0.45,4.50)	39.33(8.68,89.76)	1.58(1.49,1.67)	1.31(1.17,1.44)
Southern Latin America	1.57(0.31,3.61)	34.42(6.73,80.21)	2.23(0.52,4.80)	46.17(10.54,100.53)	1.05(0.94,1.15)	0.81(0.71,0.91)
Southern sub-Saharan Africa	1.32(0.28,3.10)	27.24(5.77,65.55)	1.89(0.45,4.03)	39.36(9.31,85.72)	1.22(0.88,1.56)	1.26(0.89,1.64)
Tropical Latin America	1.41(0.32,3.10)	29.89(6.75,66.35)	1.45(0.34,3.12)	29.12(6.87,63.48)	0.31(0.22,0.40)	0.12(0.02,0.21)
Western Europe	2.37(0.49,5.33)	50.46(10.19,115.29)	2.90(0.69,6.21)	58.14(13.66,126.12)	0.72(0.58,0.85)	0.46(0.36,0.56)
Western sub-Saharan Africa	0.41(0.08,0.98)	7.80(1.53,19.08)	0.72(0.16,1.65)	13.65(2.87,31.48)	2.21(2.10,2.33)	2.13(2.03,2.23)

Table S2. Death cases and age-standardized death rate of TBL cancer attributed to HFPG in 1990 and 2019, and its temporal trends from 1990 to 2019.

Nation	Sex	Death Cases No. (95% UI)		Change in Absolute Number (%)	ASDR per 100,000 No. (95% UI)		1990-2019 EAPC No. (95%CI)
		1990	2019		1990	2019	
Afghanistan	both	67.76(10.63,205.52)	158.01(31.04,419.37)	1.33	1.01(0.16,3.02)	1.54(0.32,3.91)	1.65(1.54,1.76)
Albania	both	23.98(4.46,58.25)	74.44(13.87,186.04)	2.10	1.25(0.24,3.01)	1.67(0.31,4.18)	0.77(0.54,1.00)
Algeria	both	99.24(19.25,239.97)	398.02(89.09,889.71)	3.01	0.95(0.18,2.26)	1.33(0.30,2.95)	1.36(1.04,1.68)
American Samoa	both	0.92(0.21,1.94)	2.65(0.72,5.24)	1.89	4.73(1.07,10.06)	5.92(1.59,11.65)	0.84(0.75,0.92)
Andorra	both	1.23(0.21,3.10)	4.03(0.80,9.22)	2.28	2.27(0.41,5.74)	2.87(0.57,6.58)	0.76(0.71,0.80)
Angola	both	36.02(6.02,93.67)	127.68(25.54,298.45)	2.55	1.06(0.18,2.71)	1.33(0.28,3.05)	0.90(0.75,1.06)
Antigua and Barbuda	both	0.53(0.12,1.16)	1.26(0.30,2.72)	1.37	0.96(0.21,2.12)	1.32(0.32,2.83)	1.16(0.90,1.43)
Argentina	both	562.68(110.34,1302.50)	1300.10(298.01,2819.93)	1.31	1.72(0.34,3.99)	2.37(0.54,5.15)	0.79(0.63,0.94)
Armenia	both	47.03(9.14,110.31)	115.78(23.18,270.15)	1.46	1.68(0.34,3.90)	2.71(0.55,6.29)	1.64(1.31,1.97)
Australia	both	318.89(64.67,729.20)	831.78(191.00,1831.30)	1.61	1.59(0.33,3.63)	1.87(0.43,4.15)	0.47(0.24,0.70)
Austria	both	153.77(32.82,354.72)	360.65(82.23,796.11)	1.35	1.25(0.26,2.88)	2.00(0.45,4.42)	1.86(1.67,2.06)
Azerbaijan	both	53.12(10.29,128.04)	173.32(35.64,403.17)	2.26	1.04(0.20,2.50)	1.88(0.40,4.33)	2.60(2.21,2.99)
Bahrain	both	8.86(1.88,19.00)	29.81(7.20,62.22)	2.37	7.19(1.61,15.09)	5.17(1.31,10.52)	-1.62(-2.08,-1.16)
Bangladesh	both	218.22(36.90,548.46)	730.54(139.30,1981.55)	2.35	0.51(0.09,1.30)	0.59(0.11,1.58)	0.61(0.35,0.86)
Barbados	both	2.80(0.66,6.14)	6.03(1.46,13.16)	1.16	0.90(0.21,1.98)	1.19(0.29,2.60)	0.67(0.49,0.85)
Belarus	both	159.46(29.61,382.20)	169.35(31.69,415.33)	0.06	1.20(0.22,2.87)	1.04(0.20,2.55)	-1.39(-1.76,-1.01)
Belgium	both	442.15(82.96,1040.20)	700.21(158.93,1538.33)	0.58	2.77(0.52,6.53)	2.98(0.67,6.58)	0.25(0.02,0.48)
Belize	both	0.58(0.13,1.32)	3.26(0.73,7.29)	4.60	0.65(0.14,1.48)	1.28(0.29,2.85)	2.02(1.47,2.57)

Benin	both	10.54(2.04,25.28)	39.01(8.15,88.77)	2.70	0.58(0.11,1.39)	0.96(0.20,2.16)	1.95(1.87,2.04)
Bermuda	both	1.65(0.35,3.78)	3.10(0.69,7.09)	0.88	2.73(0.58,6.26)	2.26(0.50,5.17)	-0.62(-0.75,-0.49)
Bhutan	both	0.66(0.13,1.72)	3.66(0.78,8.82)	4.52	0.31(0.06,0.78)	0.70(0.15,1.69)	3.08(3.00,3.15)
Bolivia (Plurinational State of)	both	24.81(4.91,61.86)	111.01(23.41,281.51)	3.48	0.87(0.18,2.17)	1.38(0.29,3.45)	1.47(1.41,1.53)
Bosnia and Herzegovina	both	95.89(19.28,221.16)	308.76(64.15,710.37)	2.22	2.41(0.50,5.55)	4.96(1.03,11.40)	2.82(2.51,3.12)
Botswana	both	6.21(1.30,14.97)	27.07(5.78,62.94)	3.36	1.21(0.26,2.86)	2.30(0.52,5.29)	2.19(1.89,2.49)
Brazil	both	1170.79(266.53,2584.62)	3327.01(784.11,7212.88)	1.84	1.43(0.33,3.14)	1.44(0.34,3.13)	0.26(0.17,0.35)
Brunei Darussalam	both	6.64(1.70,14.08)	18.99(5.12,38.20)	1.86	8.83(2.39,18.53)	8.77(2.39,17.42)	0.44(0.20,0.68)
Bulgaria	both	202.19(40.49,465.33)	410.45(78.61,980.59)	1.03	1.55(0.31,3.56)	2.84(0.54,6.83)	3.58(2.98,4.18)
Burkina Faso	both	18.21(3.33,44.14)	62.83(12.92,152.30)	2.45	0.49(0.09,1.17)	0.83(0.17,2.00)	2.10(1.96,2.25)
Burundi	both	9.32(1.73,22.66)	18.05(3.38,45.06)	0.94	0.43(0.08,1.05)	0.48(0.09,1.20)	0.24(0.15,0.32)
Cambodia	both	43.02(8.19,107.00)	284.05(60.51,647.11)	5.60	1.11(0.22,2.78)	2.71(0.59,6.14)	3.14(2.97,3.32)
Cameroon	both	21.42(4.11,52.00)	113.83(23.45,276.57)	4.31	0.58(0.11,1.41)	1.17(0.24,2.84)	2.60(2.13,3.06)
Canada	both	694.73(150.19,1578.94)	1898.16(435.61,4207.17)	1.73	2.07(0.45,4.70)	2.63(0.60,5.84)	0.49(0.36,0.63)
Cabo Verde	both	1.52(0.34,3.51)	7.43(1.78,17.24)	3.89	0.62(0.14,1.43)	1.92(0.46,4.43)	3.31(2.98,3.65)
Central African Republic	both	11.25(1.66,35.51)	23.68(3.83,73.27)	1.11	1.03(0.17,3.10)	1.19(0.21,3.55)	0.57(0.44,0.69)
Chad	both	11.86(2.24,29.44)	46.16(8.65,114.36)	2.89	0.45(0.09,1.11)	0.99(0.19,2.45)	3.01(2.76,3.27)
Chile	both	122.35(26.19,278.07)	458.55(111.09,971.00)	2.75	1.27(0.27,2.90)	1.89(0.46,4.01)	1.67(1.51,1.84)
China	both	14426.59(3112.69,33824.09)	53004.92(11784.92,121264.97)	2.67	1.85(0.41,4.28)	2.73(0.61,6.24)	1.65(1.24,2.06)

Colombia	both	257.61(59.83,561.41)	725.28(171.58,1650.28)	1.82	1.64(0.39,3.56)	1.37(0.33,3.12)	-1.01(-1.17,-0.86)
Comoros	both	0.76(0.14,1.89)	2.05(0.42,4.88)	1.70	0.37(0.07,0.93)	0.46(0.09,1.09)	0.63(0.54,0.72)
Congo	both	13.61(2.25,36.51)	35.68(7.50,83.59)	1.62	1.38(0.24,3.64)	1.59(0.35,3.62)	0.37(0.19,0.56)
Costa Rica	both	18.82(4.20,41.00)	61.20(13.46,139.40)	2.25	1.15(0.26,2.49)	1.22(0.27,2.77)	-0.11(-0.30,0.08)
Côte d'Ivoire	both	20.25(3.70,51.15)	92.14(19.33,216.48)	3.55	0.67(0.12,1.67)	1.12(0.23,2.62)	1.79(1.64,1.93)
Croatia	both	188.38(37.89,427.81)	318.75(63.92,732.90)	0.69	2.89(0.58,6.56)	3.53(0.70,8.17)	1.02(0.74,1.30)
Cuba	both	335.07(72.69,737.05)	787.63(182.12,1707.61)	1.35	3.25(0.71,7.16)	4.05(0.94,8.81)	1.01(0.79,1.24)
Cyprus	both	19.50(4.06,42.59)	61.36(13.63,130.36)	2.15	2.36(0.49,5.18)	3.03(0.67,6.46)	0.93(0.70,1.16)
Czechia	both	575.04(113.35,1286.56)	998.67(227.06,2191.23)	0.74	4.10(0.81,9.17)	4.51(1.02,9.91)	0.49(0.24,0.74)
Democratic Republic of the Congo	both	144.46(21.30,549.28)	350.53(56.37,1150.17)	1.43	1.07(0.17,4.08)	1.12(0.18,3.66)	-0.01(-0.21,0.18)
Denmark	both	157.88(33.48,363.47)	342.82(80.39,753.87)	1.17	1.90(0.40,4.39)	2.79(0.65,6.14)	1.41(1.18,1.64)
Djibouti	both	0.48(0.08,1.25)	3.73(0.68,10.35)	6.85	0.46(0.08,1.19)	0.79(0.14,2.13)	1.93(1.85,2.00)
Dominica	both	1.25(0.28,2.75)	2.49(0.57,5.40)	0.99	1.66(0.37,3.64)	2.72(0.62,5.87)	1.88(1.79,1.98)
Dominican Republic	both	16.70(3.46,38.76)	100.35(21.52,242.30)	5.01	0.50(0.10,1.16)	1.13(0.24,2.71)	3.58(3.33,3.84)
Ecuador	both	27.11(6.04,61.36)	162.31(38.24,362.71)	4.99	0.57(0.13,1.28)	1.15(0.27,2.57)	2.75(2.38,3.11)
Egypt	both	72.73(16.00,169.90)	514.97(111.02,1224.97)	6.08	0.27(0.06,0.64)	0.87(0.19,2.06)	4.27(4.14,4.40)
El Salvador	both	17.39(4.03,38.94)	73.92(17.89,167.72)	3.25	0.62(0.14,1.38)	1.24(0.30,2.82)	2.35(2.12,2.59)
Equatorial Guinea	both	1.76(0.27,5.17)	6.94(1.45,17.73)	2.95	0.95(0.15,2.75)	1.71(0.36,4.26)	2.58(2.40,2.76)
Eritrea	both	2.54(0.48,6.58)	12.43(2.56,29.31)	3.89	0.31(0.06,0.78)	0.56(0.12,1.29)	1.89(1.64,2.14)
Estonia	both	35.01(6.84,82.32)	50.05(10.65,115.60)	0.43	1.68(0.33,3.96)	1.84(0.39,4.30)	0.40(0.10,0.70)
Ethiopia	both	64.54(10.13,192.13)	119.24(22.72,295.97)	0.85	0.38(0.06,1.11)	0.34(0.06,0.85)	-0.78(-0.90,-0.65)
Micronesia (Federated States of)	both	0.99(0.20,2.43)	3.07(0.66,7.56)	2.10	2.33(0.46,5.68)	4.88(1.11,11.64)	2.59(2.27,2.90)
Fiji	both	4.49(0.99,9.78)	14.65(3.91,30.69)	2.27	1.53(0.34,3.31)	2.20(0.59,4.52)	1.01(0.89,1.13)

Finland	both	160.34(34.89,355.97)	297.08(72.50,638.35)	0.85	2.17(0.47,4.84)	2.25(0.54,4.87)	0.37(0.22,0.51)
France	both	962.83(174.29,2322.97)	2302.17(482.46,5334.96)	1.39	1.15(0.21,2.79)	1.68(0.35,3.91)	1.44(1.27,1.61)
Gabon	both	8.47(1.43,23.93)	20.76(4.28,52.82)	1.45	1.56(0.27,4.39)	2.18(0.46,5.36)	1.09(0.98,1.20)
Georgia	both	84.34(16.37,196.54)	183.76(37.85,425.37)	1.18	1.31(0.26,3.06)	3.08(0.63,7.14)	4.33(3.66,5.00)
Germany	both	3108.12(640.99,6963.36)	6794.17(1637.04,14427.71)	1.19	2.40(0.49,5.40)	3.46(0.82,7.41)	1.13(0.99,1.26)
Ghana	both	23.19(4.90,54.06)	111.87(25.19,258.18)	3.82	0.45(0.10,1.05)	0.82(0.19,1.86)	2.38(2.07,2.69)
Greece	both	371.92(73.22,873.01)	822.92(168.62,1849.19)	1.21	2.36(0.46,5.55)	3.40(0.71,7.68)	1.12(0.99,1.26)
Greenland	both	1.00(0.20,2.34)	4.02(0.85,9.20)	3.03	3.29(0.67,7.74)	6.16(1.31,14.01)	1.84(1.62,2.06)
Grenada	both	1.11(0.25,2.47)	2.28(0.55,4.84)	1.06	1.47(0.33,3.32)	2.10(0.51,4.40)	1.43(1.14,1.73)
Guam	both	1.97(0.42,4.62)	6.37(1.47,13.91)	2.23	3.21(0.67,7.48)	3.42(0.79,7.47)	0.20(-0.11,0.51)
Guatemala	both	22.46(5.13,50.48)	114.28(26.89,253.73)	4.09	0.75(0.17,1.68)	1.10(0.26,2.43)	0.83(0.34,1.32)
Guinea	both	11.82(2.16,28.52)	36.25(7.39,86.98)	2.07	0.39(0.07,0.93)	0.74(0.15,1.75)	2.28(2.12,2.43)
Guinea-Bissau	both	2.67(0.46,7.14)	6.24(1.25,15.93)	1.33	0.75(0.13,1.96)	1.06(0.21,2.62)	1.45(1.33,1.57)
Guyana	both	3.18(0.72,6.95)	7.90(1.89,17.67)	1.49	0.91(0.21,1.97)	1.34(0.32,2.95)	1.46(1.34,1.59)
Haiti	both	35.30(6.88,99.16)	92.52(19.72,235.17)	1.62	1.18(0.23,3.24)	1.50(0.32,3.79)	0.92(0.79,1.04)
Honduras	both	24.20(5.76,54.36)	165.69(37.35,407.24)	5.85	1.29(0.31,2.86)	2.95(0.67,7.23)	3.01(2.82,3.21)
Hungary	both	464.73(95.46,1072.08)	953.16(218.31,2138.88)	1.05	3.10(0.64,7.16)	4.88(1.10,11.01)	1.56(1.08,2.03)
Iceland	both	4.33(0.95,9.95)	12.53(3.02,27.79)	1.90	1.48(0.32,3.40)	2.20(0.53,4.89)	1.32(1.09,1.54)
India	both	1807.90(353.20,4285.07)	8861.51(1980.64,20244.60)	3.90	0.47(0.09,1.12)	0.82(0.19,1.86)	1.54(1.41,1.68)
Indonesia	both	721.32(140.74,1695.71)	3439.85(702.35,8312.65)	3.77	0.86(0.17,2.03)	1.83(0.38,4.42)	2.66(2.55,2.77)

Iran (Islamic Republic of)	both	154.91(29.65,381.86)	905.78(211.03,1935.79)	4.85	0.71(0.14,1.71)	1.38(0.32,2.95)	2.63(2.45,2.82)
Iraq	both	107.61(21.41,255.86)	526.44(115.41,1174.85)	3.89	1.49(0.30,3.55)	2.64(0.58,5.80)	2.18(1.86,2.50)
Ireland	both	57.13(12.13,134.48)	202.41(47.51,436.66)	2.54	1.32(0.28,3.12)	2.61(0.61,5.63)	2.49(2.36,2.62)
Israel	both	71.30(15.94,159.34)	241.86(58.02,525.52)	2.39	1.43(0.32,3.21)	2.03(0.48,4.42)	1.17(0.68,1.67)
Italy	both	2481.70(496.54,5655.24)	4304.25(958.73,9244.90)	0.73	2.69(0.54,6.15)	2.84(0.63,6.10)	0.35(-0.20,0.91)
Jamaica	both	21.92(4.91,49.03)	74.54(16.12,169.12)	2.40	1.21(0.27,2.72)	2.54(0.55,5.77)	2.59(1.99,3.20)
Japan	both	2774.06(570.62,6287.01)	6475.59(1360.18,14792.76)	1.33	1.64(0.34,3.73)	1.52(0.31,3.48)	-0.59(-0.71,-0.46)
Jordan	both	14.46(2.88,34.37)	112.37(24.49,247.41)	6.77	1.25(0.26,2.94)	2.01(0.44,4.42)	2.01(1.75,2.28)
Kazakhstan	both	251.49(52.92,585.02)	323.69(71.03,733.38)	0.29	1.97(0.42,4.55)	1.90(0.42,4.27)	-0.06(-0.21,0.09)
Kenya	both	16.69(3.04,41.19)	73.15(14.56,171.02)	3.38	0.24(0.04,0.59)	0.40(0.08,0.93)	1.76(1.58,1.95)
Kiribati	both	0.67(0.13,1.61)	2.05(0.45,4.56)	2.06	1.93(0.38,4.54)	3.21(0.70,6.97)	1.77(1.59,1.96)
Kuwait	both	7.10(1.51,15.91)	35.51(7.91,77.02)	4.00	1.49(0.32,3.32)	1.86(0.42,4.01)	0.84(0.36,1.31)
Kyrgyzstan	both	26.50(5.19,62.64)	28.22(5.66,65.79)	0.06	0.87(0.17,2.05)	0.66(0.13,1.53)	-1.14(-1.56,-0.72)
Lao People's Democratic Republic	both	32.15(6.03,83.70)	97.30(21.41,223.74)	2.03	1.70(0.33,4.40)	2.62(0.57,5.96)	1.21(1.11,1.32)
Latvia	both	48.47(9.67,114.94)	64.41(13.18,147.50)	0.33	1.33(0.27,3.15)	1.60(0.33,3.70)	0.81(0.42,1.20)
Lebanon	both	38.60(7.15,95.31)	190.49(45.35,418.12)	3.93	1.80(0.34,4.41)	3.65(0.87,8.00)	3.24(2.91,3.57)
Lesotho	both	6.74(1.32,17.46)	18.93(3.99,45.72)	1.81	0.73(0.14,1.88)	1.60(0.34,3.83)	3.26(3.06,3.47)
Liberia	both	7.17(1.36,16.98)	15.72(3.10,36.62)	1.19	0.70(0.13,1.64)	0.97(0.20,2.27)	1.84(1.49,2.20)
Libya	both	30.52(5.12,76.65)	130.48(25.74,294.18)	3.27	1.80(0.30,4.56)	2.88(0.58,6.49)	1.75(1.54,1.96)
Lithuania	both	58.49(10.85,138.78)	74.26(14.70,175.93)	0.27	1.28(0.24,3.03)	1.29(0.25,3.07)	0.11(-0.17,0.39)
Luxembourg	both	9.19(1.73,21.59)	36.90(8.62,80.60)	3.02	1.64(0.31,3.86)	3.65(0.85,7.98)	3.06(2.87,3.24)

North Macedonia	both	38.73(7.47,89.20)	159.52(32.63,370.14)	3.12	2.06(0.40,4.74)	4.76(0.98,11.01)	3.11(2.71,3.51)
Madagascar	both	14.43(2.80,34.76)	34.66(6.82,85.68)	1.40	0.33(0.06,0.78)	0.40(0.08,0.98)	0.57(0.48,0.66)
Malawi	both	11.11(2.22,26.20)	31.70(6.58,71.95)	1.85	0.35(0.07,0.82)	0.50(0.10,1.14)	1.40(1.12,1.67)
Malaysia	both	115.39(24.46,262.03)	568.21(124.14,1318.85)	3.92	1.44(0.30,3.26)	2.38(0.53,5.50)	1.60(1.14,2.05)
Maldives	both	0.65(0.12,1.66)	2.57(0.55,5.78)	2.96	1.07(0.19,2.68)	1.08(0.23,2.42)	-0.48(-0.66,-0.30)
Mali	both	10.77(2.18,25.81)	39.54(8.32,93.52)	2.67	0.30(0.06,0.71)	0.54(0.11,1.27)	2.11(2.05,2.16)
Malta	both	9.97(1.90,23.09)	23.01(4.85,50.45)	1.31	2.29(0.44,5.31)	2.32(0.49,5.10)	0.19(0.06,0.32)
Marshall Islands	both	0.61(0.12,1.59)	1.94(0.43,4.80)	2.18	4.07(0.82,10.54)	6.56(1.48,15.94)	1.90(1.76,2.04)
Mauritania	both	3.96(0.82,9.41)	11.89(2.55,27.70)	2.00	0.43(0.09,1.03)	0.65(0.14,1.50)	1.26(1.07,1.45)
Mauritius	both	9.69(2.04,21.42)	35.11(8.49,74.48)	2.62	1.42(0.30,3.11)	2.07(0.51,4.36)	1.52(1.27,1.77)
Mexico	both	776.51(196.29,1623.28)	1582.99(388.67,3380.68)	1.04	2.05(0.52,4.26)	1.42(0.35,3.03)	-1.79(-2.09,-1.49)
Republic of Moldova	both	64.42(13.24,149.65)	70.68(14.38,164.19)	0.10	1.40(0.29,3.24)	1.18(0.24,2.76)	0.20(-0.21,0.62)
Mongolia	both	10.57(2.03,25.83)	25.22(4.85,61.04)	1.39	1.09(0.21,2.63)	1.36(0.27,3.24)	0.41(0.27,0.55)
Montenegro	both	21.14(4.26,47.58)	60.58(12.89,137.60)	1.86	3.43(0.70,7.69)	5.94(1.27,13.48)	2.01(1.81,2.21)
Morocco	both	117.60(20.85,284.12)	575.56(115.57,1373.05)	3.89	0.90(0.16,2.17)	1.91(0.38,4.57)	2.56(2.39,2.73)
Mozambique	both	15.16(2.90,35.77)	60.84(12.03,145.21)	3.01	0.32(0.06,0.73)	0.66(0.13,1.58)	3.07(2.88,3.26)
Myanmar	both	337.18(68.36,890.33)	1000.99(224.63,2369.63)	1.97	1.60(0.33,4.13)	2.42(0.54,5.75)	1.38(1.23,1.53)
Namibia	both	3.12(0.66,7.51)	9.14(1.99,20.44)	1.93	0.46(0.10,1.09)	0.72(0.16,1.60)	1.71(1.59,1.83)
Nepal	both	32.19(5.75,84.49)	158.36(33.32,370.47)	3.92	0.39(0.07,1.05)	0.77(0.17,1.78)	2.16(1.99,2.32)
Netherlands	both	568.69(106.00,1313.09)	999.63(229.36,2214.84)	0.76	2.76(0.51,6.38)	2.78(0.64,6.16)	-0.02(-0.22,0.18)
New Zealand	both	62.63(13.50,145.53)	148.51(35.15,327.34)	1.37	1.56(0.34,3.61)	1.81(0.43,4.01)	-0.05(-0.44,0.33)
Nicaragua	both	10.78(2.38,24.05)	45.37(10.75,98.90)	3.21	0.80(0.18,1.77)	1.16(0.28,2.51)	1.17(1.00,1.34)
Niger	both	5.33(0.90,14.15)	33.19(6.08,86.73)	5.23	0.24(0.04,0.63)	0.54(0.10,1.41)	3.06(2.82,3.29)

Nigeria	both	121.49(22.30,309.99)	354.05(74.41,861.24)	1.91	0.32(0.06,0.81)	0.52(0.11,1.27)	1.81(1.71,1.92)
Democratic People's Republic of Korea	both	176.33(37.15,437.32)	590.72(127.05,1364.95)	2.35	1.19(0.25,2.88)	1.86(0.41,4.32)	1.64(1.58,1.70)
Northern Mariana Islands	both	0.67(0.13,1.55)	2.89(0.66,6.21)	3.29	5.34(1.08,12.12)	6.44(1.47,13.66)	0.56(0.44,0.69)
Norway	both	123.57(27.26,276.64)	238.80(57.10,517.74)	0.93	1.74(0.39,3.90)	2.38(0.57,5.16)	0.93(0.71,1.16)
Oman	both	4.29(0.83,10.66)	17.46(3.88,39.27)	3.07	0.78(0.16,1.92)	1.46(0.33,3.18)	2.56(2.28,2.84)
Pakistan	both	496.18(85.10,1194.41)	1638.53(327.71,4057.13)	2.30	0.92(0.16,2.21)	1.62(0.33,3.96)	2.11(1.80,2.42)
Palestine	both	14.98(2.74,35.72)	72.13(16.08,156.21)	3.81	1.88(0.34,4.44)	3.55(0.81,7.62)	2.38(2.11,2.65)
Panama	both	18.55(3.88,41.42)	56.54(12.70,125.62)	2.05	1.30(0.27,2.90)	1.38(0.31,3.06)	0.10(0.01,0.19)
Papua New Guinea	both	28.97(5.29,74.44)	120.87(23.88,294.30)	3.17	1.81(0.34,4.67)	2.97(0.60,7.04)	1.79(1.72,1.86)
Paraguay	both	12.93(2.78,29.72)	81.39(16.34,189.38)	5.29	0.62(0.13,1.43)	1.55(0.31,3.59)	2.89(2.55,3.22)
Peru	both	76.05(15.80,178.32)	242.95(55.32,570.04)	2.19	0.70(0.15,1.65)	0.77(0.17,1.82)	0.31(-0.03,0.65)
Philippines	both	393.94(86.43,906.43)	984.47(212.74,2258.66)	1.50	1.57(0.35,3.57)	1.42(0.32,3.23)	-1.11(-1.41,-0.81)
Poland	both	1368.00(269.02,3125.01)	3178.94(684.34,6991.05)	1.32	3.07(0.61,7.01)	4.40(0.95,9.69)	1.30(1.18,1.41)
Portugal	both	220.42(44.93,495.46)	572.34(128.39,1232.19)	1.60	1.52(0.31,3.43)	2.36(0.53,5.10)	1.67(1.34,2.00)
Puerto Rico	both	70.06(16.78,150.41)	129.98(31.68,284.47)	0.86	1.91(0.46,4.11)	1.70(0.41,3.77)	-0.59(-0.77,-0.41)
Qatar	both	2.14(0.44,4.96)	24.23(5.65,52.33)	10.31	3.12(0.68,7.00)	5.07(1.23,10.19)	1.96(1.46,2.46)
Romania	both	314.06(59.29,733.59)	804.74(165.60,1866.47)	1.56	1.07(0.20,2.50)	2.18(0.44,5.04)	2.32(2.13,2.51)
Russian Federation	both	2035.53(398.80,4788.81)	2732.89(546.07,6400.86)	0.34	1.09(0.22,2.57)	1.13(0.23,2.64)	-0.23(-0.50,0.03)
Rwanda	both	11.89(2.22,29.81)	27.38(5.41,70.47)	1.30	0.47(0.09,1.15)	0.55(0.11,1.40)	0.12(-0.14,0.38)
Saint Lucia	both	1.55(0.38,3.32)	4.10(1.04,8.71)	1.64	1.82(0.45,3.87)	1.94(0.50,4.12)	-0.04(-0.28,0.20)

Saint Vincent and the Grenadines	both	0.80(0.19,1.69)	2.04(0.51,4.36)	1.56	1.11(0.27,2.35)	1.53(0.38,3.26)	0.98(0.79,1.17)
Samoa	both	0.94(0.20,2.09)	2.27(0.53,5.14)	1.42	1.12(0.25,2.51)	1.65(0.39,3.73)	1.25(1.15,1.35)
Sao Tome and Principe	both	0.53(0.11,1.23)	1.52(0.35,3.46)	1.87	0.91(0.18,2.11)	1.77(0.40,4.02)	2.44(2.34,2.53)
Saudi Arabia	both	43.26(8.64,104.70)	184.05(40.51,403.31)	3.25	0.87(0.18,2.10)	1.38(0.31,2.95)	1.33(1.13,1.53)
Senegal	both	24.56(4.89,58.86)	89.17(19.28,205.15)	2.63	0.87(0.17,2.05)	1.38(0.30,3.15)	2.24(1.90,2.57)
Serbia	both	321.43(63.23,747.73)	852.28(184.48,1945.07)	1.65	2.73(0.54,6.29)	5.13(1.10,11.68)	2.43(2.20,2.67)
Seychelles	both	0.90(0.19,2.06)	2.93(0.71,6.21)	2.26	1.58(0.33,3.63)	2.95(0.73,6.17)	1.93(1.71,2.14)
Sierra Leone	both	5.54(1.03,13.72)	17.39(3.58,42.16)	2.14	0.31(0.06,0.77)	0.57(0.12,1.38)	2.56(2.36,2.76)
Singapore	both	76.26(17.12,168.00)	153.99(36.07,337.22)	1.02	3.82(0.87,8.38)	2.08(0.49,4.56)	-2.33(-2.53,-2.12)
Slovakia	both	143.68(26.76,337.12)	219.29(43.48,519.71)	0.53	2.36(0.44,5.53)	2.29(0.46,5.43)	-0.16(-0.42,0.09)
Slovenia	both	56.22(10.60,133.52)	114.85(24.74,267.13)	1.04	2.29(0.43,5.43)	2.60(0.55,6.06)	0.03(-0.31,0.37)
Solomon Islands	both	2.72(0.42,7.86)	12.50(2.28,33.86)	3.59	2.26(0.37,6.34)	4.66(0.91,12.15)	2.68(2.55,2.82)
Somalia	both	6.79(1.15,18.33)	21.71(3.67,60.77)	2.20	0.34(0.06,0.92)	0.39(0.07,1.06)	0.54(0.48,0.61)
South Africa	both	276.94(57.75,675.86)	824.52(193.59,1795.82)	1.98	1.45(0.31,3.51)	1.98(0.47,4.28)	1.01(0.62,1.40)
Republic of Korea	both	430.86(93.02,984.51)	2230.28(488.20,4887.95)	4.18	1.53(0.34,3.47)	2.51(0.55,5.46)	0.68(0.13,1.24)
South Sudan	both	10.82(1.85,29.07)	20.01(3.77,51.33)	0.85	0.51(0.09,1.38)	0.65(0.12,1.65)	0.86(0.76,0.96)
Spain	both	1442.31(275.98,3275.05)	2774.45(573.17,6109.23)	0.92	2.53(0.48,5.75)	2.88(0.59,6.36)	0.14(-0.07,0.35)
Sri Lanka	both	62.31(13.29,140.85)	374.72(86.62,862.07)	5.01	0.66(0.14,1.49)	1.51(0.35,3.46)	3.67(3.36,3.98)
Sudan	both	46.28(8.24,145.56)	172.09(34.35,446.38)	2.72	0.54(0.10,1.70)	1.06(0.22,2.74)	2.33(2.27,2.39)
Suriname	both	3.26(0.74,7.28)	13.37(3.33,28.46)	3.10	1.33(0.30,2.98)	2.30(0.57,4.84)	2.11(1.85,2.38)
Eswatini	both	2.98(0.58,7.45)	9.90(2.06,24.67)	2.32	1.18(0.24,2.89)	1.90(0.41,4.66)	1.97(1.57,2.36)

Sweden	both	187.67(42.00,424.54)	400.04(96.74,861.58)	1.13	1.19(0.27,2.70)	1.76(0.42,3.81)	1.60(1.35,1.84)
Switzerland	both	252.96(50.64,572.87)	354.05(83.61,777.57)	0.40	2.39(0.47,5.45)	1.97(0.47,4.33)	-0.32(-0.49,-0.16)
Syrian Arab Republic	both	36.08(7.59,84.97)	146.37(32.15,334.16)	3.06	0.77(0.16,1.79)	1.32(0.29,2.98)	1.75(1.54,1.95)
Taiwan (Province of China)	both	243.98(55.64,551.36)	1099.88(252.52,2484.9 9)	3.51	1.62(0.37,3.65)	2.76(0.63,6.24)	1.58(1.20,1.96)
Tajikistan	both	20.16(4.02,47.47)	45.39(10.01,105.72)	1.25	0.72(0.15,1.69)	1.07(0.24,2.48)	2.40(1.77,3.03)
United Republic of Tanzania	both	29.27(5.28,71.76)	120.51(23.75,310.73)	3.12	0.32(0.06,0.77)	0.57(0.11,1.45)	2.41(2.25,2.58)
Thailand	both	529.86(117.39,1200.37)	2043.12(450.08,4827.2 4)	2.86	1.69(0.38,3.82)	2.06(0.45,4.84)	0.03(-0.19,0.24)
Bahamas	both	2.10(0.45,4.78)	6.34(1.41,13.99)	2.02	1.46(0.31,3.30)	1.71(0.39,3.74)	0.63(0.56,0.70)
Gambia	both	0.89(0.17,2.16)	5.06(1.09,11.68)	4.66	0.30(0.06,0.71)	0.60(0.13,1.38)	2.31(2.16,2.46)
Timor-Leste	both	1.78(0.34,4.43)	14.79(3.12,34.02)	7.30	0.86(0.16,2.10)	1.98(0.43,4.56)	2.85(2.63,3.07)
Togo	both	4.27(0.82,10.34)	20.22(4.12,48.01)	3.73	0.42(0.09,1.01)	0.69(0.15,1.63)	1.81(1.70,1.93)
Tonga	both	1.39(0.28,3.24)	3.20(0.70,7.08)	1.31	2.76(0.56,6.45)	4.19(0.91,9.25)	1.44(1.19,1.68)
Trinidad and Tobago	both	12.93(2.89,27.16)	30.53(6.61,69.33)	1.36	1.59(0.36,3.33)	1.63(0.36,3.70)	-0.12(-0.23,0.00)
Tunisia	both	86.29(15.18,208.28)	359.08(67.48,863.88)	3.16	1.83(0.33,4.37)	2.92(0.56,7.02)	1.60(1.36,1.84)
Turkey	both	823.51(143.14,2004.29)	2721.08(571.82,6264.5 5)	2.30	2.41(0.42,5.84)	3.14(0.66,7.20)	2.97(1.79,4.17)
Turkmenistan	both	12.20(2.44,28.80)	25.79(5.13,60.76)	1.11	0.62(0.13,1.47)	0.67(0.14,1.56)	-0.05(-0.57,0.48)
Uganda	both	20.93(4.07,50.62)	73.22(16.06,165.66)	2.50	0.37(0.07,0.88)	0.62(0.14,1.38)	1.78(1.65,1.91)
Ukraine	both	1051.38(204.69,2482.15)	885.15(174.92,2127.29)	-0.16	1.42(0.28,3.36)	1.15(0.23,2.77)	-1.73(-2.07,-1.38)
United Arab Emirates	both	6.52(1.39,15.33)	73.34(16.76,167.05)	10.24	2.85(0.62,6.63)	3.86(0.90,8.86)	1.02(0.39,1.66)

United Kingdom	both	3346.20(773.51,7382.46)	5593.34(1407.51,11681.66)	0.67	3.49(0.80,7.70)	4.16(1.04,8.72)	0.74(0.58,0.90)
United States of America	both	14022.50(3234.79,30355.59)	27719.29(6895.63,57335.30)	0.98	4.33(0.99,9.40)	4.77(1.19,9.89)	1.09(0.74,1.44)
Uruguay	both	41.54(7.47,100.38)	132.89(27.72,300.86)	2.20	1.03(0.19,2.49)	2.44(0.50,5.53)	3.06(2.51,3.61)
Uzbekistan	both	69.23(14.09,162.32)	208.23(43.80,468.45)	2.01	0.61(0.13,1.42)	1.09(0.24,2.43)	2.08(1.96,2.19)
Vanuatu	both	1.10(0.20,2.93)	5.61(1.14,13.30)	4.11	1.96(0.35,5.14)	3.56(0.72,8.34)	2.07(1.97,2.18)
Venezuela (Bolivarian Republic of)	both	148.41(33.82,326.07)	645.85(146.94,1504.82)	3.35	1.67(0.38,3.66)	2.28(0.52,5.30)	1.31(1.04,1.58)
Viet nam	both	416.42(77.47,1021.91)	1951.39(413.66,4423.27)	3.69	1.07(0.20,2.59)	2.31(0.50,5.20)	2.66(2.61,2.70)
Virginia	both	340.92(76.03,741.03)	718.99(177.02,1552.05)	1.11	4.79(1.07,10.42)	4.89(1.20,10.55)	0.70(0.37,1.04)
Yemen	both	22.08(3.79,62.89)	101.63(19.82,258.22)	3.60	0.50(0.09,1.43)	0.88(0.17,2.25)	2.27(2.08,2.46)
Zambia	both	13.61(2.50,33.55)	44.57(9.37,106.93)	2.28	0.58(0.11,1.39)	0.80(0.17,1.91)	1.01(0.86,1.16)
Zimbabwe	both	33.07(7.05,75.21)	89.46(20.57,195.79)	1.71	0.91(0.20,2.05)	1.44(0.34,3.13)	1.74(1.67,1.80)
Monaco	both	1.42(0.29,3.38)	5.15(1.15,11.62)	2.63	1.93(0.38,4.64)	5.15(1.15,11.68)	3.83(3.34,4.34)
San Marino	both	0.65(0.12,1.54)	1.94(0.39,4.93)	1.99	1.87(0.35,4.46)	2.90(0.57,7.52)	1.78(1.67,1.89)
Saint Kitts and Nevis	both	0.54(0.13,1.14)	0.93(0.22,2.00)	0.74	1.36(0.33,2.92)	1.53(0.37,3.24)	0.35(0.16,0.54)
Cook Islands	both	0.45(0.09,1.03)	1.21(0.27,2.60)	1.67	3.93(0.80,8.88)	4.80(1.07,10.36)	0.48(0.29,0.67)
Nauru	both	0.11(0.02,0.27)	0.18(0.04,0.43)	0.65	3.68(0.72,8.98)	5.58(1.25,12.83)	1.44(1.34,1.54)
Niue	both	0.08(0.02,0.17)	0.13(0.03,0.27)	0.63	3.53(0.84,7.66)	5.92(1.51,12.41)	1.79(1.62,1.95)
Palau	both	0.41(0.10,0.92)	1.41(0.35,3.03)	2.45	4.52(1.08,10.09)	7.30(1.82,15.48)	1.67(1.52,1.82)
Tokelau	both	0.03(0.01,0.07)	0.05(0.01,0.10)	0.64	2.06(0.45,4.72)	3.63(0.87,7.96)	2.07(2.01,2.12)
Tuvalu	both	0.14(0.03,0.36)	0.38(0.09,0.87)	1.66	2.22(0.48,5.45)	3.94(0.93,8.77)	2.00(1.88,2.11)

Afghanistan	female	9.48(1.64,24.25)	38.85(8.40,97.30)	3.10	0.30(0.05,0.79)	0.68(0.15,1.62)	3.16(2.89,3.43)
Albania	female	3.66(0.68,9.17)	13.44(2.39,33.84)	2.67	0.36(0.07,0.90)	0.57(0.10,1.44)	1.13(0.75,1.51)
Algeria	female	15.84(2.92,39.26)	85.33(17.47,194.47)	4.39	0.31(0.06,0.76)	0.60(0.12,1.34)	2.32(2.22,2.41)
American Samoa	female	0.19(0.04,0.44)	0.81(0.20,1.78)	3.17	1.97(0.41,4.44)	3.46(0.83,7.62)	2.05(1.95,2.14)
Andorra	female	0.14(0.03,0.38)	0.68(0.12,1.72)	3.82	0.54(0.10,1.44)	0.95(0.17,2.43)	1.94(1.71,2.17)
Angola	female	3.86(0.69,10.14)	22.92(4.35,57.97)	4.94	0.23(0.04,0.62)	0.45(0.08,1.12)	2.33(2.20,2.46)
Antigua and Barbuda	female	0.12(0.02,0.29)	0.45(0.10,1.03)	2.69	0.38(0.08,0.89)	0.88(0.19,1.98)	2.83(2.72,2.95)
Argentina	female	107.41(19.78,256.85)	382.68(72.97,887.37)	2.56	0.59(0.11,1.41)	1.22(0.23,2.83)	2.49(2.42,2.55)
Armenia	female	8.51(1.53,20.26)	22.04(4.49,52.24)	1.59	0.56(0.10,1.35)	0.90(0.18,2.13)	1.72(1.39,2.06)
Australia	female	72.36(12.78,175.02)	288.25(54.17,686.65)	2.98	0.64(0.11,1.55)	1.21(0.23,2.88)	2.05(1.53,2.56)
Austria	female	38.33(6.73,93.18)	122.98(22.55,289.39)	2.21	0.48(0.08,1.18)	1.23(0.22,2.88)	3.66(3.46,3.86)
Azerbaijan	female	9.92(1.82,23.99)	35.49(6.89,86.23)	2.58	0.35(0.06,0.84)	0.77(0.15,1.89)	3.53(3.14,3.91)
Bahrain	female	1.50(0.31,3.38)	7.09(1.67,14.83)	3.73	2.62(0.56,5.85)	2.92(0.67,5.95)	0.21(-0.35,0.78)
Bangladesh	female	23.87(3.83,64.87)	156.55(26.76,415.38)	5.56	0.13(0.02,0.36)	0.27(0.05,0.72)	2.43(2.22,2.63)
Barbados	female	0.85(0.17,1.98)	2.22(0.47,5.10)	1.59	0.47(0.09,1.07)	0.79(0.17,1.82)	2.23(1.98,2.47)
Belarus	female	26.63(5.03,64.86)	24.50(4.29,61.24)	-0.08	0.31(0.06,0.76)	0.23(0.04,0.59)	-1.72(-1.99,-1.44)
Belgium	female	60.91(11.02,143.14)	183.82(35.76,428.36)	2.02	0.64(0.12,1.51)	1.44(0.28,3.35)	3.26(2.97,3.54)
Belize	female	0.17(0.03,0.40)	0.99(0.20,2.35)	4.77	0.37(0.07,0.88)	0.78(0.16,1.86)	2.84(2.43,3.24)
Benin	female	2.08(0.37,5.28)	10.35(2.00,25.07)	3.98	0.22(0.04,0.56)	0.46(0.09,1.11)	2.63(2.47,2.80)
Bermuda	female	0.40(0.07,0.98)	0.87(0.17,2.10)	1.18	1.12(0.20,2.78)	1.08(0.21,2.62)	-0.55(-0.70,-0.40)
Bhutan	female	0.15(0.02,0.43)	0.93(0.16,2.39)	5.41	0.13(0.02,0.40)	0.37(0.07,0.94)	3.59(3.54,3.64)
Bolivia (Plurinational State of)	female	7.53(1.37,18.92)	40.08(7.61,105.98)	4.32	0.48(0.09,1.20)	0.93(0.18,2.43)	2.11(2.01,2.20)
Bosnia and Herzegovina	female	16.90(3.07,40.29)	66.10(13.85,157.48)	2.91	0.77(0.14,1.83)	1.90(0.40,4.56)	3.64(3.38,3.90)

Botswana	female	1.46(0.29,3.67)	8.85(1.61,21.73)	5.04	0.53(0.10,1.31)	1.32(0.24,3.17)	3.57(3.37,3.76)
Brazil	female	309.78(57.56,725.59)	1295.03(246.69,3003.03)	3.18	0.71(0.13,1.66)	1.00(0.19,2.32)	1.45(1.33,1.58)
Brunei Darussalam	female	1.87(0.40,4.16)	7.27(1.62,15.43)	2.88	4.81(1.03,10.63)	6.04(1.33,12.71)	0.76(0.52,0.99)
Bulgaria	female	31.95(6.24,78.60)	78.44(15.46,187.98)	1.45	0.46(0.09,1.12)	0.93(0.18,2.27)	3.72(3.22,4.23)
Burkina Faso	female	4.10(0.71,10.53)	14.75(2.64,37.13)	2.59	0.21(0.04,0.54)	0.35(0.06,0.89)	1.94(1.72,2.16)
Burundi	female	1.59(0.28,4.13)	3.18(0.49,8.43)	1.00	0.13(0.02,0.35)	0.17(0.03,0.45)	0.66(0.58,0.74)
Cambodia	female	8.01(1.39,20.49)	71.51(14.44,169.45)	7.93	0.36(0.06,0.92)	1.14(0.23,2.64)	4.06(3.78,4.33)
Cameroon	female	5.21(0.99,13.88)	30.90(5.86,80.87)	4.93	0.28(0.05,0.73)	0.61(0.11,1.58)	2.74(2.46,3.01)
Canada	female	198.16(34.18,488.55)	764.70(145.31,1834.31)	2.86	1.05(0.18,2.60)	1.96(0.37,4.71)	1.78(1.45,2.11)
Cabo Verde	female	0.61(0.11,1.48)	2.95(0.59,6.90)	3.82	0.44(0.08,1.06)	1.24(0.25,2.94)	3.90(3.72,4.09)
Central African Republic	female	1.23(0.24,3.16)	3.19(0.57,8.01)	1.59	0.23(0.04,0.58)	0.31(0.06,0.80)	1.31(1.23,1.39)
Chad	female	2.28(0.38,6.03)	8.27(1.51,21.56)	2.63	0.17(0.03,0.46)	0.39(0.07,1.00)	3.10(3.01,3.19)
Chile	female	35.79(7.03,85.52)	190.08(38.25,425.26)	4.31	0.68(0.13,1.62)	1.39(0.28,3.11)	2.92(2.64,3.19)
China	female	4614.47(885.82,11474.13)	15785.20(2935.11,38934.40)	2.42	1.14(0.22,2.79)	1.54(0.29,3.79)	1.21(0.79,1.64)
Colombia	female	88.24(17.17,204.92)	304.07(61.52,722.07)	2.45	1.09(0.21,2.51)	1.04(0.21,2.46)	-0.61(-0.77,-0.46)
Comoros	female	0.15(0.03,0.38)	0.58(0.10,1.47)	2.85	0.14(0.03,0.37)	0.23(0.04,0.59)	1.57(1.46,1.69)
Congo	female	2.21(0.42,5.87)	9.18(1.67,23.80)	3.15	0.41(0.08,1.07)	0.78(0.14,1.96)	2.32(2.20,2.44)
Costa Rica	female	4.65(0.91,10.91)	18.48(3.75,45.23)	2.98	0.55(0.11,1.28)	0.67(0.14,1.64)	0.21(0.00,0.42)
Côte d'Ivoire	female	3.59(0.66,9.00)	21.74(4.12,53.04)	5.06	0.26(0.05,0.65)	0.54(0.10,1.32)	2.69(2.62,2.77)
Croatia	female	30.37(5.82,72.39)	70.14(12.99,169.79)	1.31	0.78(0.15,1.87)	1.34(0.25,3.27)	2.57(2.27,2.88)
Cuba	female	80.02(15.57,186.18)	238.25(46.24,564.14)	1.98	1.53(0.30,3.56)	2.29(0.44,5.42)	1.89(1.62,2.16)
Cyprus	female	3.29(0.71,7.55)	12.30(2.47,27.49)	2.74	0.76(0.17,1.77)	1.16(0.23,2.62)	1.54(1.26,1.83)

Czechia	female	79.18(14.97,183.57)	274.99(60.39,637.39)	2.47	0.93(0.18,2.17)	2.18(0.47,5.09)	3.15(2.92,3.37)
Democratic Republic of the Congo	female	17.19(2.95,46.19)	63.51(11.27,164.48)	2.70	0.24(0.04,0.69)	0.37(0.07,0.98)	1.26(0.88,1.64)
Denmark	female	49.43(9.05,120.75)	155.47(30.10,368.87)	2.14	1.08(0.20,2.63)	2.36(0.45,5.65)	2.73(2.42,3.05)
Djibouti	female	0.09(0.02,0.23)	0.69(0.12,1.78)	6.73	0.17(0.03,0.43)	0.31(0.06,0.79)	2.21(2.12,2.29)
Dominica	female	0.34(0.07,0.80)	0.66(0.14,1.56)	0.94	0.75(0.15,1.75)	1.37(0.29,3.24)	2.49(2.28,2.70)
Dominican Republic	female	5.44(0.92,13.25)	35.15(6.64,91.22)	5.47	0.32(0.05,0.79)	0.75(0.14,1.96)	3.78(3.38,4.19)
Ecuador	female	9.68(1.84,23.12)	71.50(14.39,162.17)	6.39	0.40(0.08,0.96)	0.95(0.19,2.17)	3.34(2.95,3.73)
Egypt	female	25.63(4.80,62.67)	167.46(32.59,445.09)	5.53	0.20(0.04,0.49)	0.68(0.13,1.77)	4.32(4.22,4.43)
El Salvador	female	6.85(1.26,16.52)	33.05(7.02,82.24)	3.83	0.45(0.08,1.09)	0.95(0.20,2.38)	2.42(2.26,2.58)
Equatorial Guinea	female	0.18(0.03,0.51)	2.14(0.39,5.73)	10.75	0.18(0.03,0.51)	0.90(0.17,2.36)	6.59(6.17,7.01)
Eritrea	female	0.56(0.09,1.61)	3.72(0.68,9.32)	5.66	0.12(0.02,0.34)	0.28(0.05,0.70)	2.96(2.68,3.23)
Estonia	female	6.45(1.17,15.82)	13.54(2.52,33.69)	1.10	0.47(0.09,1.15)	0.76(0.14,1.90)	2.07(1.83,2.31)
Ethiopia	female	6.09(0.97,16.94)	20.48(3.04,59.03)	2.36	0.07(0.01,0.21)	0.12(0.02,0.34)	1.36(1.22,1.51)
Micronesia (Federated States of)	female	0.22(0.05,0.56)	0.89(0.18,2.22)	2.97	1.01(0.21,2.51)	2.69(0.56,6.69)	3.47(3.16,3.78)
Fiji	female	1.21(0.24,2.88)	5.07(1.14,11.49)	3.18	0.76(0.15,1.79)	1.37(0.31,3.07)	2.09(1.97,2.22)
Finland	female	35.32(6.56,83.72)	100.94(20.54,232.32)	1.86	0.75(0.14,1.79)	1.36(0.28,3.17)	2.66(2.45,2.87)
France	female	117.79(20.67,295.61)	573.67(105.78,1417.83)	3.87	0.23(0.04,0.57)	0.73(0.13,1.80)	4.95(4.56,5.34)
Gabon	female	1.28(0.24,3.24)	4.32(0.79,11.09)	2.37	0.44(0.08,1.11)	0.86(0.16,2.21)	2.29(2.01,2.57)
Georgia	female	14.96(2.79,36.88)	25.84(5.16,60.58)	0.73	0.39(0.07,0.97)	0.71(0.14,1.67)	3.11(2.58,3.64)
Germany	female	670.62(129.81,1553.72)	2227.50(455.84,5013.97)	2.32	0.80(0.15,1.87)	2.07(0.42,4.70)	3.27(3.05,3.50)
Ghana	female	8.15(1.49,20.44)	31.23(6.12,77.29)	2.83	0.29(0.06,0.73)	0.40(0.08,1.01)	0.70(0.43,0.97)
Greece	female	57.45(10.12,140.16)	148.59(27.53,346.75)	1.59	0.66(0.12,1.62)	1.08(0.20,2.54)	1.55(1.40,1.71)

Greenland	female	0.23(0.04,0.60)	1.13(0.22,2.92)	3.80	1.50(0.25,3.83)	3.71(0.73,9.56)	2.12(1.49,2.75)
Grenada	female	0.29(0.06,0.68)	0.69(0.14,1.54)	1.35	0.66(0.13,1.55)	1.20(0.25,2.68)	2.40(2.13,2.67)
Guam	female	0.53(0.11,1.28)	2.10(0.43,5.08)	2.95	1.69(0.36,4.10)	2.14(0.44,5.20)	0.72(0.36,1.08)
Guatemala	female	9.39(1.77,22.50)	43.73(9.27,100.45)	3.66	0.64(0.12,1.51)	0.76(0.16,1.75)	-0.45(-0.91,0.01)
Guinea	female	2.29(0.41,5.80)	7.90(1.38,19.60)	2.45	0.15(0.03,0.38)	0.33(0.06,0.81)	2.72(2.64,2.79)
Guinea-Bissau	female	0.42(0.08,1.07)	1.59(0.29,3.99)	2.81	0.24(0.04,0.60)	0.48(0.09,1.20)	2.71(2.62,2.79)
Guyana	female	0.97(0.19,2.24)	2.92(0.63,6.81)	2.01	0.53(0.11,1.22)	0.91(0.20,2.11)	1.89(1.73,2.04)
Haiti	female	7.73(1.52,18.67)	29.53(5.69,72.12)	2.82	0.49(0.10,1.18)	0.87(0.17,2.14)	2.13(2.05,2.21)
Honduras	female	10.04(1.94,24.61)	89.53(16.68,241.68)	7.92	1.05(0.20,2.54)	3.03(0.57,8.08)	3.66(3.32,4.01)
Hungary	female	92.84(17.65,224.36)	312.69(60.09,756.25)	2.37	1.04(0.20,2.52)	2.69(0.51,6.52)	3.57(3.07,4.07)
Iceland	female	1.75(0.32,4.24)	5.30(1.00,12.61)	2.02	1.09(0.20,2.63)	1.74(0.33,4.13)	1.36(1.07,1.65)
India	female	313.90(58.30,778.01)	2394.61(479.96,5813.08)	6.63	0.17(0.03,0.43)	0.43(0.09,1.05)	2.87(2.67,3.07)
Indonesia	female	180.35(32.37,489.54)	1009.83(166.15,2977.17)	4.60	0.40(0.07,1.06)	1.01(0.17,2.97)	3.17(3.02,3.33)
Iran (Islamic Republic of)	female	28.66(5.32,68.48)	295.51(62.98,679.40)	9.31	0.29(0.05,0.69)	0.91(0.19,2.07)	4.17(4.01,4.33)
Iraq	female	16.93(2.99,43.26)	138.07(30.07,328.65)	7.16	0.45(0.08,1.16)	1.32(0.29,3.10)	4.12(3.84,4.40)
Ireland	female	16.78(3.00,42.28)	78.51(14.74,184.50)	3.68	0.69(0.12,1.74)	1.89(0.36,4.47)	3.65(3.52,3.78)
Israel	female	23.20(4.30,54.62)	84.78(16.34,198.92)	2.65	0.85(0.16,2.00)	1.26(0.24,2.95)	1.39(0.93,1.84)
Italy	female	399.76(76.81,944.19)	1050.60(205.25,2400.36)	1.63	0.73(0.14,1.73)	1.22(0.24,2.80)	2.15(1.68,2.63)
Jamaica	female	5.41(1.04,12.44)	17.05(3.58,40.54)	2.15	0.55(0.11,1.26)	1.10(0.23,2.60)	2.33(2.04,2.63)
Japan	female	584.82(106.73,1416.96)	1590.16(285.61,3908.02)	1.72	0.59(0.11,1.44)	0.59(0.11,1.45)	-0.64(-0.88,-0.41)

Jordan	female	2.38(0.44,5.71)	21.56(4.57,49.87)	8.04	0.44(0.08,1.04)	0.84(0.17,1.93)	2.27(1.94,2.60)
Kazakhstan	female	62.34(11.53,145.32)	79.51(15.71,180.70)	0.28	0.80(0.15,1.87)	0.80(0.16,1.81)	-0.07(-0.29,0.14)
Kenya	female	2.19(0.32,6.48)	12.50(2.05,33.62)	4.72	0.06(0.01,0.18)	0.12(0.02,0.32)	2.85(2.59,3.11)
Kiribati	female	0.11(0.02,0.27)	0.33(0.07,0.78)	1.99	0.57(0.11,1.38)	0.94(0.19,2.21)	1.31(1.12,1.50)
Kuwait	female	1.38(0.27,3.41)	5.97(1.24,13.45)	3.32	0.74(0.14,1.80)	0.75(0.16,1.68)	0.13(-0.22,0.49)
Kyrgyzstan	female	5.62(1.03,13.88)	7.15(1.27,17.54)	0.27	0.31(0.06,0.77)	0.30(0.05,0.72)	-0.37(-0.70,-0.05)
Lao People's Democratic Republic	female	6.09(1.11,15.42)	25.72(5.35,63.45)	3.22	0.61(0.11,1.57)	1.31(0.27,3.19)	2.37(2.25,2.49)
Latvia	female	9.34(1.79,23.57)	15.32(2.94,37.22)	0.64	0.39(0.07,0.98)	0.57(0.11,1.39)	1.89(1.54,2.24)
Lebanon	female	6.84(1.28,16.63)	55.28(10.95,129.85)	7.08	0.64(0.12,1.56)	1.92(0.38,4.54)	4.44(4.16,4.72)
Lesotho	female	1.45(0.26,3.61)	5.39(0.99,13.84)	2.72	0.28(0.05,0.68)	0.78(0.15,2.00)	4.43(4.13,4.73)
Liberia	female	1.38(0.26,3.41)	4.08(0.75,10.00)	1.96	0.30(0.06,0.72)	0.50(0.09,1.22)	2.69(2.22,3.17)
Libya	female	2.03(0.36,5.10)	16.16(3.34,38.13)	6.98	0.25(0.04,0.64)	0.72(0.15,1.70)	3.93(3.64,4.23)
Lithuania	female	8.73(1.56,21.33)	14.36(2.65,35.84)	0.65	0.30(0.05,0.74)	0.37(0.07,0.92)	1.10(0.86,1.34)
Luxembourg	female	1.52(0.28,3.72)	10.17(2.09,23.69)	5.69	0.45(0.08,1.11)	1.83(0.37,4.24)	5.49(5.20,5.79)
North Macedonia	female	6.04(1.17,14.45)	28.04(5.79,65.90)	3.64	0.63(0.12,1.51)	1.63(0.33,3.85)	3.71(3.37,4.05)
Madagascar	female	2.63(0.50,6.80)	9.17(1.71,23.97)	2.49	0.12(0.02,0.30)	0.19(0.04,0.50)	1.56(1.46,1.66)
Malawi	female	2.32(0.43,5.78)	7.32(1.28,18.18)	2.15	0.13(0.02,0.33)	0.20(0.04,0.51)	1.60(1.54,1.66)
Malaysia	female	27.34(5.18,63.74)	169.29(34.48,405.96)	5.19	0.66(0.12,1.54)	1.42(0.29,3.40)	2.89(2.38,3.41)
Maldives	female	0.09(0.02,0.22)	0.55(0.11,1.29)	5.14	0.34(0.07,0.83)	0.48(0.10,1.13)	0.51(0.25,0.76)
Mali	female	3.08(0.57,7.80)	12.59(2.36,30.95)	3.09	0.17(0.03,0.43)	0.35(0.07,0.87)	2.56(2.53,2.60)
Malta	female	1.15(0.22,2.67)	4.44(0.89,10.08)	2.86	0.47(0.09,1.10)	0.84(0.17,1.93)	2.00(1.90,2.10)
Marshall Islands	female	0.12(0.02,0.27)	0.50(0.11,1.16)	3.34	1.49(0.32,3.49)	3.41(0.76,7.80)	2.81(2.67,2.95)
Mauritania	female	1.34(0.23,3.73)	4.59(0.82,11.41)	2.43	0.27(0.05,0.75)	0.51(0.09,1.25)	1.93(1.77,2.10)
Mauritius	female	2.20(0.45,5.02)	9.80(2.12,21.99)	3.46	0.58(0.12,1.33)	1.04(0.23,2.32)	2.12(1.87,2.38)

Mexico	female	253.57(53.53,547.51)	525.19(107.04,1190.02)	1.07	1.30(0.28,2.80)	0.87(0.18,1.98)	-1.84(-2.07,-1.62)
Republic of Moldova	female	12.95(2.44,31.39)	14.97(2.84,35.86)	0.16	0.49(0.09,1.18)	0.42(0.08,1.02)	0.33(-0.04,0.70)
Mongolia	female	2.46(0.45,6.35)	5.33(0.96,13.23)	1.17	0.45(0.08,1.18)	0.53(0.09,1.32)	-0.02(-0.27,0.24)
Montenegro	female	4.30(0.80,10.54)	13.77(2.81,32.88)	2.20	1.24(0.23,3.00)	2.46(0.50,5.87)	2.69(2.48,2.89)
Morocco	female	9.86(1.83,24.68)	70.36(13.94,166.89)	6.14	0.16(0.03,0.40)	0.49(0.10,1.17)	4.15(4.05,4.24)
Mozambique	female	3.13(0.57,7.94)	14.20(2.52,37.96)	3.54	0.12(0.02,0.30)	0.27(0.05,0.72)	3.17(2.96,3.39)
Myanmar	female	85.94(15.20,225.28)	350.83(72.03,840.07)	3.08	0.76(0.13,1.93)	1.47(0.30,3.47)	2.33(2.22,2.44)
Namibia	female	0.79(0.15,1.97)	3.01(0.59,7.36)	2.80	0.21(0.04,0.53)	0.40(0.08,0.97)	2.08(1.89,2.27)
Nepal	female	5.86(0.88,17.83)	43.64(7.61,112.50)	6.45	0.15(0.02,0.47)	0.41(0.07,1.06)	3.42(3.31,3.53)
Netherlands	female	72.66(13.29,175.49)	334.01(63.44,789.53)	3.60	0.62(0.11,1.50)	1.76(0.34,4.17)	3.89(3.58,4.20)
New Zealand	female	20.01(3.78,48.43)	64.55(12.01,154.47)	2.23	0.91(0.18,2.21)	1.48(0.28,3.55)	1.15(0.78,1.52)
Nicaragua	female	2.51(0.43,6.38)	16.39(3.26,37.01)	5.52	0.34(0.06,0.87)	0.75(0.15,1.70)	2.54(2.18,2.91)
Niger	female	0.95(0.15,2.67)	7.11(1.12,19.35)	6.51	0.09(0.01,0.26)	0.22(0.03,0.60)	3.13(2.90,3.35)
Nigeria	female	24.29(4.06,65.88)	96.17(17.31,250.49)	2.96	0.12(0.02,0.34)	0.27(0.05,0.69)	2.54(2.12,2.96)
Democratic People's Republic of Korea	female	59.51(10.85,148.24)	204.80(38.54,511.79)	2.44	0.65(0.13,1.63)	1.06(0.20,2.65)	1.73(1.68,1.79)
Northern Mariana Islands	female	0.12(0.02,0.29)	0.66(0.13,1.51)	4.64	2.15(0.44,5.22)	2.90(0.60,6.60)	0.99(0.93,1.05)
Norway	female	31.89(5.98,75.42)	102.96(20.26,241.95)	2.23	0.79(0.15,1.86)	1.92(0.38,4.52)	2.99(2.77,3.22)
Oman	female	0.75(0.13,2.07)	4.49(0.92,10.42)	4.97	0.29(0.05,0.79)	0.80(0.17,1.85)	3.48(3.13,3.83)
Pakistan	female	40.36(7.11,100.09)	257.78(50.32,636.69)	5.39	0.17(0.03,0.42)	0.54(0.11,1.35)	4.47(4.20,4.74)
Palestine	female	1.83(0.32,4.93)	12.75(2.62,29.88)	5.97	0.42(0.07,1.11)	1.20(0.25,2.77)	3.98(3.75,4.21)
Panama	female	3.82(0.74,8.91)	18.59(3.85,44.51)	3.87	0.54(0.10,1.26)	0.86(0.18,2.06)	1.35(1.17,1.52)
Papua New Guinea	female	3.96(0.56,11.68)	20.11(3.46,55.08)	4.08	0.49(0.07,1.45)	1.04(0.18,2.79)	2.69(2.66,2.72)
Paraguay	female	3.52(0.65,8.55)	18.50(3.65,45.72)	4.26	0.32(0.06,0.77)	0.66(0.13,1.63)	2.53(2.32,2.73)

Peru	female	25.76(4.59,63.99)	111.53(21.08,277.36)	3.33	0.46(0.08,1.14)	0.67(0.13,1.68)	0.95(0.66,1.23)
Philippines	female	110.75(19.98,260.33)	346.92(67.70,845.72)	2.13	0.88(0.16,2.07)	0.92(0.18,2.25)	-0.65(-0.97,-0.34)
Poland	female	241.23(46.22,564.10)	853.86(156.60,2026.12)	2.54	0.91(0.17,2.14)	2.03(0.37,4.82)	3.21(3.02,3.40)
Portugal	female	43.48(8.21,100.51)	135.74(28.42,307.05)	2.12	0.52(0.10,1.21)	0.94(0.20,2.15)	2.23(2.00,2.45)
Puerto Rico	female	21.67(4.54,48.23)	49.45(10.79,113.93)	1.28	1.09(0.23,2.43)	1.13(0.24,2.60)	-0.06(-0.24,0.12)
Qatar	female	0.24(0.04,0.59)	3.42(0.84,7.27)	13.34	0.92(0.17,2.33)	2.84(0.69,5.85)	4.51(3.86,5.17)
Romania	female	51.43(8.83,125.23)	166.16(30.70,408.25)	2.23	0.32(0.06,0.79)	0.76(0.14,1.86)	3.08(2.98,3.17)
Russian Federation	female	381.08(70.52,930.80)	586.71(105.30,1429.17)	0.54	0.31(0.06,0.76)	0.38(0.07,0.93)	0.71(0.47,0.94)
Rwanda	female	2.42(0.42,6.78)	7.68(1.40,19.17)	2.17	0.17(0.03,0.46)	0.25(0.05,0.64)	1.39(1.24,1.54)
Saint Lucia	female	0.55(0.11,1.21)	1.44(0.32,3.21)	1.62	1.12(0.23,2.47)	1.26(0.28,2.82)	-0.15(-0.51,0.21)
Saint Vincent and the Grenadines	female	0.30(0.06,0.66)	0.74(0.16,1.66)	1.50	0.72(0.15,1.63)	1.13(0.24,2.53)	1.70(1.45,1.95)
Samoa	female	0.26(0.05,0.62)	0.78(0.17,1.83)	2.03	0.60(0.12,1.45)	1.10(0.24,2.57)	1.98(1.90,2.07)
Sao Tome and Principe	female	0.12(0.02,0.30)	0.43(0.07,1.23)	2.62	0.38(0.07,0.93)	0.93(0.15,2.66)	3.25(3.17,3.34)
Saudi Arabia	female	6.39(1.16,17.04)	40.50(7.90,96.05)	5.34	0.30(0.05,0.80)	0.73(0.15,1.71)	2.36(1.88,2.85)
Senegal	female	4.41(0.80,10.85)	21.63(4.29,51.87)	3.91	0.32(0.06,0.81)	0.64(0.13,1.52)	2.94(2.66,3.23)
Serbia	female	56.17(10.43,133.80)	210.18(41.45,502.02)	2.74	0.93(0.18,2.19)	2.32(0.47,5.61)	3.76(3.52,4.00)
Seychelles	female	0.17(0.03,0.41)	0.77(0.17,1.72)	3.52	0.50(0.10,1.20)	1.40(0.31,3.12)	3.48(3.14,3.83)
Sierra Leone	female	0.95(0.16,2.37)	4.82(0.83,12.15)	4.07	0.11(0.02,0.28)	0.31(0.05,0.77)	4.10(3.86,4.34)
Singapore	female	21.33(3.98,49.33)	45.43(8.52,106.52)	1.13	1.96(0.37,4.54)	1.14(0.21,2.66)	-1.95(-2.22,-1.69)
Slovakia	female	17.29(3.19,41.95)	47.15(9.06,118.13)	1.73	0.48(0.09,1.18)	0.85(0.16,2.13)	2.35(2.08,2.62)
Slovenia	female	11.28(2.14,28.05)	29.69(5.74,72.18)	1.63	0.74(0.14,1.85)	1.18(0.22,2.90)	1.81(1.52,2.10)
Solomon Islands	female	0.29(0.06,0.77)	1.88(0.36,4.74)	5.42	0.51(0.10,1.32)	1.36(0.27,3.29)	3.37(3.17,3.57)
Somalia	female	0.94(0.15,2.52)	3.74(0.56,11.18)	2.97	0.09(0.01,0.23)	0.11(0.02,0.34)	1.28(1.17,1.39)

South Africa	female	87.70(16.58,211.29)	302.19(59.74,692.88)	2.45	0.79(0.15,1.89)	1.23(0.24,2.83)	1.58(1.23,1.94)
Republic of Korea	female	98.99(18.99,238.11)	554.01(105.45,1315.13)	4.60	0.63(0.12,1.51)	1.06(0.20,2.52)	0.56(-0.05,1.17)
South Sudan	female	1.61(0.27,4.34)	3.36(0.58,9.07)	1.09	0.17(0.03,0.45)	0.22(0.04,0.61)	1.01(0.91,1.10)
Spain	female	153.27(29.39,353.43)	479.99(90.29,1106.05)	2.13	0.46(0.09,1.05)	0.89(0.17,2.05)	3.15(2.80,3.49)
Sri Lanka	female	15.42(2.99,35.99)	119.62(27.13,268.51)	6.76	0.34(0.07,0.79)	0.86(0.20,1.95)	3.75(3.55,3.95)
Sudan	female	6.65(1.07,17.68)	38.97(7.62,94.04)	4.86	0.17(0.03,0.45)	0.52(0.10,1.26)	4.07(3.98,4.15)
Suriname	female	0.94(0.19,2.27)	4.61(0.99,10.85)	3.88	0.73(0.15,1.73)	1.44(0.31,3.40)	2.77(2.49,3.05)
Eswatini	female	0.80(0.14,2.06)	3.22(0.56,8.20)	3.05	0.56(0.10,1.44)	1.04(0.19,2.65)	2.44(2.04,2.83)
Sweden	female	55.32(9.70,134.19)	184.53(37.89,430.56)	2.34	0.63(0.11,1.53)	1.54(0.32,3.59)	3.50(3.16,3.85)
Switzerland	female	52.49(10.12,123.97)	122.26(23.27,284.63)	1.33	0.83(0.16,1.98)	1.26(0.24,2.95)	2.25(1.83,2.68)
Syrian Arab Republic	female	7.14(1.34,18.09)	38.25(7.69,89.19)	4.36	0.33(0.06,0.84)	0.78(0.16,1.83)	2.70(2.35,3.04)
Taiwan (Province of China)	female	76.77(14.70,179.94)	409.95(80.12,966.81)	4.34	1.10(0.21,2.58)	1.88(0.37,4.44)	1.57(1.25,1.88)
Tajikistan	female	5.12(0.92,12.52)	13.41(2.57,32.46)	1.62	0.34(0.06,0.82)	0.60(0.12,1.43)	3.19(2.55,3.83)
United Republic of Tanzania	female	5.73(0.99,14.27)	28.69(5.10,71.64)	4.01	0.12(0.02,0.29)	0.25(0.04,0.63)	3.13(2.98,3.29)
Thailand	female	182.82(35.27,445.16)	716.38(141.37,1719.23)	2.92	1.07(0.20,2.60)	1.29(0.26,3.09)	-0.26(-0.53,0.01)
Bahamas	female	0.48(0.09,1.13)	1.85(0.36,4.29)	2.81	0.59(0.11,1.37)	0.90(0.18,2.08)	1.85(1.71,1.99)
Gambia	female	0.21(0.04,0.52)	1.73(0.32,4.54)	7.31	0.15(0.03,0.36)	0.39(0.07,1.02)	3.47(3.33,3.62)
Timor-Leste	female	0.38(0.06,1.00)	3.86(0.74,9.45)	9.28	0.35(0.06,0.92)	1.02(0.19,2.50)	3.99(3.69,4.29)
Togo	female	1.03(0.18,2.60)	5.98(1.08,14.68)	4.82	0.20(0.03,0.49)	0.35(0.06,0.85)	1.98(1.84,2.11)
Tonga	female	0.28(0.05,0.66)	0.74(0.16,1.76)	1.63	1.05(0.20,2.45)	1.78(0.39,4.23)	1.79(1.66,1.92)
Trinidad and Tobago	female	2.91(0.59,6.32)	7.41(1.60,17.63)	1.54	0.68(0.14,1.48)	0.76(0.16,1.80)	0.17(-0.01,0.35)
Tunisia	female	3.97(0.75,9.83)	29.97(6.30,73.37)	6.54	0.18(0.03,0.44)	0.48(0.10,1.18)	3.41(3.26,3.56)
Turkey	female	96.76(16.49,240.15)	455.46(88.89,1090.99)	3.71	0.55(0.09,1.35)	0.99(0.19,2.37)	3.61(2.61,4.63)

Turkmenistan	female	2.77(0.50,6.74)	6.48(1.25,15.70)	1.34	0.25(0.05,0.62)	0.31(0.06,0.76)	0.68(0.24,1.11)
Uganda	female	3.74(0.67,9.36)	23.14(4.41,57.01)	5.18	0.13(0.02,0.32)	0.34(0.06,0.83)	3.57(3.37,3.77)
Ukraine	female	211.89(36.50,518.98)	138.80(24.36,348.92)	-0.34	0.44(0.08,1.08)	0.28(0.05,0.73)	-2.27(-2.61,-1.94)
United Arab Emirates	female	1.27(0.20,3.52)	13.20(2.51,31.39)	9.38	1.50(0.24,4.29)	2.54(0.46,6.52)	2.04(0.94,3.14)
United Kingdom	female	987.88(188.79,2333.99)	2396.84(497.45,5444.69)	1.43	1.77(0.34,4.17)	3.24(0.67,7.32)	2.17(2.06,2.28)
United States of America	female	4573.39(868.53,10720.13)	10896.95(2253.71,24720.54)	1.38	2.47(0.47,5.77)	3.40(0.71,7.72)	2.00(1.61,2.40)
Uruguay	female	5.71(1.03,14.06)	28.21(5.30,68.36)	3.94	0.25(0.04,0.61)	0.88(0.16,2.14)	4.81(4.40,5.22)
Uzbekistan	female	16.80(3.04,40.80)	52.57(10.03,125.63)	2.13	0.26(0.05,0.63)	0.53(0.10,1.25)	2.58(2.44,2.72)
Vanuatu	female	0.15(0.03,0.40)	1.07(0.21,2.60)	5.99	0.59(0.10,1.52)	1.42(0.29,3.43)	2.89(2.69,3.09)
Venezuela (Bolivarian Republic of)	female	52.24(9.86,121.62)	232.93(46.32,589.06)	3.46	1.10(0.21,2.56)	1.53(0.30,3.87)	1.43(1.26,1.59)
Vietnam	female	92.33(18.33,233.96)	566.19(106.39,1378.58)	5.13	0.41(0.08,1.02)	1.15(0.22,2.78)	3.79(3.73,3.86)
Virginia	female	108.67(20.84,254.35)	284.34(58.95,670.19)	1.62	2.65(0.50,6.21)	3.50(0.72,8.28)	1.78(1.34,2.22)
Yemen	female	2.94(0.44,9.03)	20.59(3.80,49.97)	6.00	0.13(0.02,0.41)	0.35(0.06,0.84)	4.05(3.75,4.35)
Zambia	female	2.61(0.49,6.94)	10.72(1.97,27.30)	3.11	0.23(0.04,0.60)	0.36(0.07,0.89)	1.49(1.29,1.70)
Zimbabwe	female	10.03(1.86,24.10)	43.56(8.36,105.74)	3.34	0.55(0.10,1.30)	1.19(0.23,2.87)	3.42(3.05,3.79)
Monaco	female	0.34(0.07,0.86)	1.70(0.33,4.12)	4.03	0.76(0.14,1.94)	3.09(0.60,7.53)	5.53(4.76,6.30)
San Marino	female	0.11(0.02,0.26)	0.51(0.09,1.47)	3.72	0.56(0.10,1.37)	1.41(0.25,4.14)	4.13(3.77,4.49)
Saint Kitts and Nevis	female	0.20(0.04,0.47)	0.31(0.07,0.74)	0.50	0.92(0.18,2.11)	0.98(0.21,2.30)	0.84(0.62,1.06)
Cook Islands	female	0.08(0.02,0.19)	0.23(0.05,0.54)	1.86	1.47(0.30,3.44)	1.81(0.42,4.16)	0.58(0.51,0.65)
Nauru	female	0.02(0.00,0.06)	0.05(0.01,0.14)	1.26	1.62(0.30,4.06)	2.94(0.53,7.39)	1.94(1.61,2.27)
Niue	female	0.03(0.01,0.06)	0.04(0.01,0.10)	0.75	1.88(0.39,4.39)	3.63(0.84,8.35)	2.30(2.19,2.41)

Palau	female	0.18(0.04,0.43)	0.67(0.14,1.50)	2.76	3.80(0.76,9.17)	6.82(1.48,15.34)	2.04(1.88,2.19)
Tokelau	female	0.01(0.00,0.02)	0.02(0.00,0.05)	0.77	1.36(0.26,3.29)	2.73(0.58,6.77)	2.42(2.35,2.49)
Tuvalu	female	0.04(0.01,0.10)	0.13(0.03,0.30)	2.09	1.10(0.23,2.67)	2.39(0.50,5.67)	2.64(2.49,2.79)
Afghanistan	male	58.28(7.19,188.10)	119.16(17.96,337.41)	1.04	1.62(0.20,5.17)	2.51(0.40,6.83)	1.72(1.63,1.81)
Albania	male	20.32(3.13,51.18)	61.00(9.76,159.74)	2.00	2.35(0.36,5.86)	2.92(0.46,7.61)	0.48(0.22,0.73)
Algeria	male	83.40(14.11,205.99)	312.68(59.08,734.51)	2.75	1.56(0.27,3.81)	2.01(0.38,4.66)	1.07(0.73,1.40)
American Samoa	male	0.72(0.13,1.58)	1.84(0.37,3.86)	1.55	7.70(1.43,16.51)	8.71(1.79,18.10)	0.46(0.35,0.58)
Andorra	male	1.09(0.17,2.81)	3.35(0.54,7.78)	2.08	4.02(0.63,10.38)	4.85(0.79,11.26)	0.65(0.61,0.69)
Angola	male	32.16(4.85,86.62)	104.76(16.28,252.47)	2.26	1.94(0.30,5.12)	2.50(0.40,5.84)	0.98(0.83,1.13)
Antigua and Barbuda	male	0.41(0.07,0.94)	0.81(0.15,1.81)	0.98	1.78(0.30,4.12)	1.85(0.33,4.17)	0.26(-0.13,0.65)
Argentina	male	455.27(73.60,1083.01)	917.42(155.18,2120.55)	1.02	3.20(0.52,7.59)	3.89(0.66,8.98)	0.28(0.09,0.46)
Armenia	male	38.52(6.16,94.26)	93.75(15.02,224.09)	1.43	3.21(0.51,7.83)	5.21(0.83,12.45)	1.64(1.30,1.98)
Australia	male	246.54(40.03,590.39)	543.53(94.83,1274.70)	1.20	2.96(0.48,6.98)	2.66(0.46,6.24)	-0.47(-0.62,-0.31)
Austria	male	115.43(17.73,278.90)	237.67(39.47,556.27)	1.06	2.54(0.39,6.16)	2.96(0.49,6.92)	0.68(0.48,0.87)
Azerbaijan	male	43.21(6.95,108.85)	137.83(22.99,335.31)	2.19	2.06(0.32,5.16)	3.28(0.54,8.05)	2.15(1.71,2.59)
Bahrain	male	7.36(1.34,16.36)	22.72(4.37,48.72)	2.09	11.83(2.24,25.70)	7.26(1.46,15.25)	-2.18(-2.65,-1.71)
Bangladesh	male	194.35(29.90,511.13)	573.99(89.71,1650.97)	1.95	0.82(0.13,2.15)	0.87(0.14,2.49)	0.41(0.14,0.68)
Barbados	male	1.94(0.32,4.52)	3.82(0.66,8.91)	0.96	1.53(0.25,3.55)	1.70(0.29,3.97)	-0.32(-0.60,-0.03)
Belarus	male	132.84(21.10,331.14)	144.85(23.59,364.41)	0.09	2.75(0.43,6.86)	2.41(0.39,6.02)	-1.33(-1.74,-0.93)
Belgium	male	381.24(62.37,929.87)	516.39(84.07,1202.13)	0.35	5.94(0.98,14.49)	4.91(0.80,11.42)	-0.79(-1.02,-0.56)
Belize	male	0.41(0.07,1.01)	2.28(0.38,5.42)	4.53	0.94(0.15,2.32)	1.76(0.29,4.17)	1.59(0.94,2.25)
Benin	male	8.46(1.26,20.83)	28.66(4.62,69.36)	2.39	0.95(0.14,2.34)	1.59(0.25,3.75)	2.05(1.92,2.18)
Bermuda	male	1.25(0.21,2.97)	2.24(0.38,5.24)	0.78	5.12(0.86,11.98)	3.85(0.65,9.03)	-0.81(-0.97,-0.65)
Bhutan	male	0.52(0.08,1.41)	2.73(0.43,7.17)	4.27	0.52(0.08,1.42)	1.03(0.16,2.69)	2.64(2.51,2.77)

Bolivia (Plurinational State of)	male	17.28(2.51,48.01)	70.93(11.29,184.68)	3.11	1.34(0.20,3.69)	1.89(0.30,4.90)	1.09(1.02,1.16)
Bosnia and Herzegovina	male	78.98(12.66,189.74)	242.67(42.21,574.12)	2.07	4.74(0.76,11.25)	8.88(1.55,20.99)	2.43(2.10,2.75)
Botswana	male	4.75(0.78,12.15)	18.21(3.29,44.05)	2.84	2.17(0.36,5.44)	3.81(0.69,9.22)	1.82(1.41,2.24)
Brazil	male	861.01(137.02,2005.58)	2031.98(335.60,4655.76)	1.36	2.29(0.37,5.30)	2.02(0.34,4.63)	-0.23(-0.34,-0.11)
Brunei Darussalam	male	4.76(0.97,10.70)	11.72(2.36,24.68)	1.46	14.71(2.95,33.33)	13.55(2.79,27.88)	0.50(0.14,0.86)
Bulgaria	male	170.24(27.83,400.96)	332.01(53.66,818.67)	0.95	2.82(0.46,6.65)	5.26(0.85,12.97)	3.71(3.07,4.36)
Burkina Faso	male	14.11(2.13,36.01)	48.08(7.90,120.58)	2.41	0.79(0.12,1.99)	1.44(0.24,3.56)	2.35(2.20,2.49)
Burundi	male	7.73(1.16,19.59)	14.87(2.36,38.19)	0.92	0.80(0.12,2.02)	0.80(0.13,2.01)	-0.20(-0.28,-0.11)
Cambodia	male	35.01(5.20,91.56)	212.54(37.44,511.30)	5.07	2.16(0.32,5.53)	5.20(0.91,12.33)	3.14(3.00,3.28)
Cameroon	male	16.20(2.45,41.16)	82.93(12.87,210.13)	4.12	0.91(0.14,2.24)	1.83(0.29,4.66)	2.66(2.11,3.21)
Canada	male	496.57(79.45,1202.30)	1133.46(189.25,2641.27)	1.28	3.49(0.56,8.45)	3.43(0.57,8.00)	-0.42(-0.52,-0.31)
Cabo Verde	male	0.91(0.15,2.24)	4.48(0.75,11.65)	3.94	0.88(0.14,2.18)	2.95(0.49,7.68)	3.08(2.42,3.74)
Central African Republic	male	10.01(1.22,32.90)	20.49(2.66,66.68)	1.05	2.04(0.27,6.54)	2.41(0.34,7.59)	0.64(0.49,0.79)
Chad	male	9.58(1.38,25.28)	37.89(5.80,101.52)	2.95	0.75(0.11,1.98)	1.49(0.23,4.02)	2.68(2.37,2.99)
Chile	male	86.56(14.17,205.21)	268.47(47.99,607.00)	2.10	2.02(0.33,4.79)	2.52(0.45,5.68)	0.96(0.83,1.08)
China	male	9812.11(1542.63,24231.02)	37219.71(6193.73,91103.80)	2.79	2.76(0.43,6.75)	4.22(0.70,10.21)	1.86(1.45,2.27)
Colombia	male	169.36(29.94,387.62)	421.21(72.34,1008.33)	1.49	2.25(0.40,5.12)	1.79(0.31,4.28)	-1.12(-1.34,-0.90)
Comoros	male	0.61(0.10,1.59)	1.48(0.22,3.81)	1.42	0.63(0.10,1.63)	0.75(0.11,1.91)	0.53(0.42,0.64)
Congo	male	11.40(1.61,31.53)	26.50(4.41,64.94)	1.32	2.69(0.40,7.31)	2.56(0.45,6.13)	-0.41(-0.59,-0.23)

Costa Rica	male	14.17(2.44,32.51)	42.72(7.49,101.82)	2.01	1.80(0.31,4.13)	1.87(0.33,4.47)	-0.12(-0.32,0.09)
Côte d'Ivoire	male	16.67(2.52,44.49)	70.40(11.29,175.96)	3.22	1.04(0.16,2.74)	1.71(0.28,4.25)	1.70(1.50,1.90)
Croatia	male	158.00(25.41,369.23)	248.61(41.05,592.61)	0.57	6.15(1.01,14.32)	6.46(1.06,15.45)	0.42(0.12,0.72)
Cuba	male	255.05(44.02,580.96)	549.39(100.90,1233.71)	1.15	5.06(0.87,11.51)	6.11(1.12,13.74)	0.79(0.58,1.00)
Cyprus	male	16.21(2.76,36.76)	49.05(8.60,107.59)	2.03	4.21(0.72,9.49)	5.14(0.90,11.29)	0.82(0.55,1.10)
Czechia	male	495.86(86.55,1142.78)	723.68(131.85,1615.51)	0.46	8.79(1.53,20.15)	7.53(1.37,16.89)	-0.41(-0.66,-0.17)
Democratic Republic of the Congo	male	127.27(15.41,522.41)	287.03(35.85,1064.10)	1.26	1.97(0.25,8.07)	2.17(0.28,8.04)	0.17(0.04,0.30)
Denmark	male	108.44(17.37,266.04)	187.35(30.20,445.09)	0.73	3.03(0.48,7.43)	3.32(0.54,7.88)	0.39(0.18,0.60)
Djibouti	male	0.39(0.06,1.07)	3.04(0.45,9.05)	6.88	0.79(0.12,2.16)	1.25(0.19,3.66)	1.61(1.50,1.71)
Dominica	male	0.91(0.16,2.13)	1.83(0.33,4.09)	1.01	3.03(0.51,7.10)	4.22(0.76,9.41)	1.14(1.07,1.22)
Dominican Republic	male	11.27(1.73,27.78)	65.20(10.48,165.85)	4.79	0.69(0.11,1.69)	1.56(0.25,3.92)	3.55(3.34,3.76)
Ecuador	male	17.43(2.82,41.52)	90.81(15.69,215.88)	4.21	0.74(0.12,1.76)	1.38(0.24,3.26)	2.42(2.04,2.79)
Egypt	male	47.10(7.30,118.92)	347.50(59.44,889.93)	6.38	0.34(0.05,0.86)	1.04(0.18,2.66)	4.15(4.00,4.30)
El Salvador	male	10.54(1.74,25.21)	40.87(7.05,95.29)	2.88	0.82(0.14,1.95)	1.62(0.28,3.79)	2.45(2.05,2.85)
Equatorial Guinea	male	1.57(0.21,4.81)	4.80(0.78,12.91)	2.05	1.98(0.26,6.03)	2.91(0.48,7.61)	1.62(1.51,1.73)
Eritrea	male	1.99(0.30,5.46)	8.71(1.42,21.81)	3.39	0.65(0.10,1.77)	1.04(0.17,2.57)	1.43(1.15,1.71)
Estonia	male	28.56(4.40,70.11)	36.51(5.80,88.11)	0.28	3.87(0.60,9.50)	3.65(0.58,8.81)	-0.13(-0.51,0.26)
Ethiopia	male	58.45(8.12,180.90)	98.76(15.53,255.31)	0.69	0.67(0.09,2.02)	0.55(0.09,1.42)	-1.06(-1.20,-0.92)
Micronesia (Federated States of)	male	0.77(0.13,1.98)	2.18(0.35,5.85)	1.85	3.83(0.64,9.82)	7.66(1.28,19.71)	2.44(2.10,2.78)
Fiji	male	3.27(0.58,7.52)	9.57(1.94,20.87)	1.93	2.39(0.43,5.39)	3.37(0.71,7.22)	0.81(0.62,1.00)
Finland	male	125.02(20.69,290.82)	196.14(33.89,453.27)	0.57	4.57(0.75,10.64)	3.39(0.59,7.82)	-0.96(-1.08,-0.84)
France	male	845.04(132.52,2087.96)	1728.51(272.52,4156.94)	1.05	2.45(0.38,6.04)	2.86(0.45,6.89)	0.53(0.38,0.67)

Gabon	male	7.19(1.00,21.26)	16.43(2.70,42.27)	1.28	3.14(0.47,8.95)	3.82(0.67,9.74)	0.60(0.48,0.72)
Georgia	male	69.37(10.90,170.28)	157.92(26.73,365.86)	1.28	2.75(0.43,6.71)	6.41(1.08,14.89)	4.41(3.67,5.15)
Germany	male	2437.49(411.30,5714.82)	4566.67(813.26,10366.38)	0.87	5.10(0.86,11.97)	5.17(0.92,11.75)	-0.18(-0.31,-0.06)
Ghana	male	15.04(2.37,37.47)	80.64(13.82,192.58)	4.36	0.64(0.10,1.55)	1.40(0.25,3.33)	3.40(2.95,3.85)
Greece	male	314.47(51.88,772.66)	674.33(116.66,1572.70)	1.14	4.44(0.73,10.92)	6.14(1.05,14.36)	0.98(0.85,1.11)
Greenland	male	0.76(0.12,1.89)	2.89(0.48,6.96)	2.80	5.44(0.90,13.30)	8.46(1.42,20.30)	1.45(1.40,1.51)
Grenada	male	0.81(0.14,1.90)	1.59(0.29,3.53)	0.95	2.63(0.47,6.13)	3.25(0.60,7.14)	1.03(0.34,1.72)
Guam	male	1.44(0.23,3.53)	4.27(0.73,9.87)	1.97	4.97(0.79,12.27)	4.93(0.84,11.42)	0.05(-0.32,0.42)
Guatemala	male	13.07(2.08,31.59)	70.55(12.61,165.10)	4.40	0.86(0.14,2.08)	1.52(0.27,3.55)	1.86(1.28,2.44)
Guinea	male	9.53(1.38,24.28)	28.35(4.71,68.99)	1.98	0.63(0.09,1.60)	1.14(0.19,2.78)	2.13(1.89,2.37)
Guinea-Bissau	male	2.26(0.31,6.36)	4.66(0.73,12.69)	1.06	1.29(0.18,3.56)	1.83(0.29,4.87)	1.48(1.35,1.60)
Guyana	male	2.20(0.37,5.07)	4.98(0.91,11.58)	1.26	1.33(0.22,3.03)	1.86(0.34,4.27)	1.32(1.21,1.44)
Haiti	male	27.57(3.99,84.62)	62.99(9.72,181.85)	1.28	2.01(0.30,6.09)	2.20(0.35,6.33)	0.40(0.22,0.57)
Honduras	male	14.17(2.40,34.96)	76.16(12.52,199.43)	4.38	1.54(0.26,3.81)	2.87(0.47,7.50)	2.43(2.22,2.64)
Hungary	male	371.89(61.14,879.98)	640.47(109.08,1510.06)	0.72	6.11(1.02,14.46)	8.02(1.37,18.95)	0.83(0.30,1.35)
Iceland	male	2.57(0.41,6.17)	7.24(1.20,16.94)	1.81	1.95(0.31,4.68)	2.70(0.45,6.34)	1.20(0.96,1.45)
India	male	1494.00(239.96,3713.03)	6466.91(1163.49,15475.88)	3.33	0.76(0.12,1.86)	1.25(0.22,2.96)	1.36(1.24,1.48)
Indonesia	male	540.98(83.93,1316.16)	2430.02(400.53,6124.01)	3.49	1.40(0.22,3.39)	2.82(0.47,7.06)	2.55(2.43,2.68)
Iran (Islamic Republic of)	male	126.24(20.49,322.24)	610.27(104.90,1395.65)	3.83	1.13(0.18,2.85)	1.85(0.32,4.22)	2.14(1.89,2.39)
Iraq	male	90.68(14.38,223.92)	388.37(68.50,908.23)	3.28	2.62(0.42,6.46)	4.11(0.73,9.47)	1.72(1.40,2.04)
Ireland	male	40.36(6.37,99.93)	123.90(20.32,287.00)	2.07	2.14(0.34,5.29)	3.45(0.57,8.01)	1.78(1.60,1.97)

Israel	male	48.11(7.96,114.99)	157.08(27.47,363.89)	2.27	2.14(0.35,5.09)	2.93(0.51,6.82)	1.06(0.51,1.61)
Italy	male	2081.94(339.03,4873.14)	3253.65(572.87,7263.69)	0.56	5.34(0.87,12.48)	4.89(0.85,10.90)	-0.17(-0.77,0.44)
Jamaica	male	16.50(2.74,38.66)	57.49(10.21,134.93)	2.48	1.99(0.33,4.66)	4.10(0.73,9.60)	2.57(1.87,3.27)
Japan	male	2189.24(356.38,5167.79)	4885.43(808.25,11457.14)	1.23	3.20(0.52,7.51)	2.74(0.45,6.44)	-0.79(-0.93,-0.65)
Jordan	male	12.08(1.99,29.63)	90.81(17.36,208.44)	6.52	2.07(0.34,5.09)	3.09(0.60,7.07)	1.86(1.53,2.20)
Kazakhstan	male	189.15(30.32,462.94)	244.18(39.83,585.79)	0.29	3.93(0.63,9.53)	3.62(0.60,8.65)	-0.11(-0.28,0.05)
Kenya	male	14.51(2.34,35.88)	60.64(9.89,148.88)	3.18	0.43(0.07,1.05)	0.75(0.12,1.83)	1.96(1.64,2.29)
Kiribati	male	0.56(0.09,1.39)	1.72(0.33,3.94)	2.07	3.81(0.62,9.16)	6.61(1.28,14.95)	2.05(1.82,2.27)
Kuwait	male	5.72(1.02,13.54)	29.55(5.45,65.97)	4.17	2.02(0.37,4.71)	2.58(0.49,5.68)	0.94(0.46,1.42)
Kyrgyzstan	male	20.87(3.20,51.24)	21.07(3.12,51.82)	0.01	1.74(0.27,4.28)	1.15(0.17,2.80)	-1.52(-2.01,-1.02)
Lao People's Democratic Republic	male	26.06(4.08,69.32)	71.58(12.11,178.30)	1.75	3.00(0.47,8.04)	4.13(0.71,10.05)	0.84(0.75,0.93)
Latvia	male	39.13(6.07,97.45)	49.09(7.66,120.66)	0.25	3.00(0.47,7.44)	3.34(0.52,8.23)	0.50(0.05,0.96)
Lebanon	male	31.76(4.82,82.72)	135.21(24.82,309.81)	3.26	3.02(0.47,7.72)	5.76(1.05,13.22)	3.09(2.74,3.44)
Lesotho	male	5.29(0.85,14.12)	13.54(2.21,34.65)	1.56	1.44(0.23,3.79)	2.96(0.49,7.54)	2.96(2.73,3.18)
Liberia	male	5.79(0.88,14.26)	11.63(1.91,28.80)	1.01	1.04(0.16,2.51)	1.45(0.24,3.54)	1.83(1.50,2.16)
Libya	male	28.50(4.61,71.17)	114.32(20.23,266.45)	3.01	3.25(0.53,8.06)	4.98(0.88,11.47)	1.61(1.40,1.82)
Lithuania	male	49.76(7.60,121.55)	59.90(9.23,147.85)	0.20	2.87(0.44,6.99)	2.80(0.43,6.93)	-0.03(-0.37,0.31)
Luxembourg	male	7.67(1.22,18.99)	26.73(4.62,60.35)	2.49	3.46(0.55,8.58)	5.82(1.00,13.14)	1.99(1.80,2.19)
North Macedonia	male	32.69(5.41,78.15)	131.48(23.50,314.59)	3.02	3.64(0.60,8.65)	8.25(1.46,19.64)	2.98(2.54,3.42)
Madagascar	male	11.80(1.94,29.77)	25.48(3.80,67.49)	1.16	0.52(0.09,1.30)	0.63(0.10,1.66)	0.61(0.49,0.74)
Malawi	male	8.79(1.43,21.74)	24.38(3.83,58.43)	1.77	0.61(0.10,1.48)	0.94(0.15,2.24)	1.66(1.28,2.05)
Malaysia	male	88.05(13.72,208.43)	398.93(67.86,983.62)	3.53	2.30(0.36,5.42)	3.36(0.58,8.17)	0.98(0.48,1.47)

Maldives	male	0.56(0.09,1.50)	2.03(0.33,4.72)	2.62	1.48(0.25,3.82)	1.61(0.27,3.75)	-0.17(-0.35,0.01)
Mali	male	7.69(1.15,19.50)	26.95(4.47,67.34)	2.50	0.44(0.07,1.11)	0.71(0.12,1.79)	1.79(1.70,1.88)
Malta	male	8.82(1.44,20.80)	18.57(3.13,41.66)	1.11	4.69(0.77,11.09)	4.12(0.70,9.31)	-0.27(-0.46,-0.08)
Marshall Islands	male	0.49(0.08,1.39)	1.43(0.24,3.84)	1.90	7.13(1.15,19.77)	9.45(1.66,24.42)	1.24(1.07,1.41)
Mauritania	male	2.62(0.40,6.68)	7.30(1.13,19.13)	1.78	0.62(0.09,1.58)	0.79(0.12,2.02)	0.66(0.44,0.88)
Mauritius	male	7.49(1.26,17.49)	25.31(4.55,55.35)	2.38	2.57(0.43,5.98)	3.43(0.63,7.47)	1.26(0.99,1.53)
Mexico	male	522.94(94.64,1148.27)	1057.80(193.12,2365.70)	1.02	2.86(0.52,6.28)	2.07(0.38,4.62)	-1.69(-2.03,-1.35)
Republic of Moldova	male	51.46(8.13,124.05)	55.72(8.98,134.22)	0.08	2.73(0.43,6.50)	2.27(0.37,5.43)	0.12(-0.31,0.56)
Mongolia	male	8.11(1.26,21.18)	19.90(3.24,50.80)	1.45	1.97(0.32,5.11)	2.63(0.43,6.67)	0.75(0.62,0.89)
Montenegro	male	16.85(2.79,39.36)	46.80(7.96,109.40)	1.78	6.42(1.07,14.79)	10.32(1.76,24.11)	1.69(1.50,1.88)
Morocco	male	107.74(16.68,264.22)	505.20(88.60,1224.69)	3.69	1.66(0.26,4.06)	3.38(0.60,8.23)	2.35(2.18,2.51)
Mozambique	male	12.03(1.83,29.65)	46.65(7.40,112.61)	2.88	0.56(0.09,1.36)	1.25(0.20,2.97)	3.39(3.12,3.66)
Myanmar	male	251.24(37.73,717.25)	650.16(107.09,1697.54)	1.59	2.65(0.40,7.57)	3.77(0.64,9.74)	1.15(0.96,1.33)
Namibia	male	2.33(0.36,5.75)	6.13(1.00,14.66)	1.63	0.79(0.12,1.90)	1.21(0.20,2.85)	1.74(1.62,1.86)
Nepal	male	26.33(3.94,71.59)	114.73(19.44,277.72)	3.36	0.63(0.09,1.71)	1.15(0.20,2.80)	1.91(1.72,2.11)
Netherlands	male	496.02(79.95,1180.96)	665.62(109.82,1565.89)	0.34	5.90(0.95,14.04)	4.05(0.67,9.54)	-1.52(-1.72,-1.31)
New Zealand	male	42.62(6.81,104.37)	83.96(13.97,198.15)	0.97	2.49(0.40,6.09)	2.22(0.37,5.24)	-1.00(-1.36,-0.64)
Nicaragua	male	8.27(1.41,19.20)	28.98(5.27,66.79)	2.50	1.40(0.24,3.19)	1.69(0.31,3.93)	0.58(0.46,0.70)
Niger	male	4.38(0.62,12.16)	26.09(3.80,72.16)	4.95	0.40(0.06,1.10)	0.89(0.13,2.42)	3.16(2.87,3.46)
Nigeria	male	97.21(13.79,259.29)	257.88(41.29,658.29)	1.65	0.56(0.08,1.47)	0.79(0.13,1.99)	1.30(1.18,1.41)
Democratic People's Republic of Korea	male	116.81(16.86,308.44)	385.92(60.90,972.91)	2.30	2.24(0.34,5.75)	3.20(0.50,7.98)	1.33(1.26,1.40)
Northern Mariana Islands	male	0.56(0.09,1.32)	2.23(0.42,4.96)	3.01	8.06(1.38,18.97)	10.13(1.86,22.05)	0.75(0.64,0.87)

Norway	male	91.68(15.12,215.81)	135.84(22.44,311.70)	0.48	3.00(0.50,7.11)	2.94(0.48,6.74)	-0.26(-0.55,0.03)
Oman	male	3.54(0.56,9.09)	12.97(2.27,30.40)	2.67	1.31(0.21,3.32)	2.09(0.38,4.73)	2.11(1.90,2.33)
Pakistan	male	455.82(71.27,1118.13)	1380.76(233.33,3569.07)	2.03	1.51(0.24,3.71)	2.64(0.45,6.68)	2.06(1.71,2.41)
Palestine	male	13.15(2.11,32.41)	59.37(10.38,130.22)	3.52	3.64(0.58,8.88)	6.40(1.15,14.02)	2.15(1.93,2.37)
Panama	male	14.73(2.50,34.18)	37.94(6.56,89.35)	1.58	2.06(0.35,4.77)	1.94(0.34,4.55)	-0.24(-0.35,-0.13)
Papua New Guinea	male	25.01(4.05,64.68)	100.76(17.35,254.74)	3.03	3.13(0.52,8.17)	4.74(0.81,11.65)	1.50(1.41,1.59)
Paraguay	male	9.42(1.50,22.71)	62.89(10.17,152.36)	5.68	0.98(0.16,2.37)	2.57(0.42,6.19)	3.02(2.62,3.43)
Peru	male	50.29(7.68,123.00)	131.42(20.95,333.26)	1.61	0.97(0.15,2.36)	0.88(0.14,2.23)	-0.13(-0.55,0.29)
Philippines	male	283.20(44.78,690.49)	637.55(98.80,1636.22)	1.25	2.33(0.38,5.61)	2.06(0.32,5.19)	-1.13(-1.43,-0.84)
Poland	male	1126.76(182.00,2693.08)	2325.08(408.17,5377.50)	1.06	6.20(1.00,14.74)	7.74(1.35,17.86)	0.70(0.59,0.82)
Portugal	male	176.94(29.28,409.42)	436.60(75.96,987.26)	1.47	2.90(0.48,6.69)	4.16(0.72,9.43)	1.42(1.06,1.78)
Puerto Rico	male	48.38(8.60,109.19)	80.53(15.14,182.39)	0.66	2.89(0.51,6.53)	2.44(0.46,5.53)	-0.78(-0.99,-0.57)
Qatar	male	1.90(0.33,4.55)	20.81(4.27,46.29)	9.94	4.97(0.93,11.52)	5.83(1.26,12.07)	0.79(0.34,1.25)
Romania	male	262.63(41.94,637.57)	638.57(100.78,1524.22)	1.43	1.98(0.31,4.79)	3.98(0.63,9.55)	2.26(2.03,2.49)
Russian Federation	male	1654.45(252.12,4139.81)	2146.18(348.67,5239.96)	0.30	2.67(0.41,6.68)	2.43(0.39,5.92)	-0.64(-0.93,-0.35)
Rwanda	male	9.47(1.45,24.51)	19.70(3.02,56.11)	1.08	0.86(0.13,2.18)	1.02(0.16,2.82)	0.13(-0.13,0.38)
Saint Lucia	male	1.00(0.18,2.30)	2.66(0.50,5.86)	1.65	2.88(0.54,6.55)	2.76(0.52,6.06)	-0.30(-0.52,-0.08)
Saint Vincent and the Grenadines	male	0.50(0.09,1.13)	1.30(0.24,2.95)	1.60	1.66(0.29,3.73)	1.93(0.35,4.31)	0.22(0.01,0.43)
Samoa	male	0.68(0.12,1.61)	1.49(0.27,3.46)	1.19	1.71(0.30,4.03)	2.25(0.41,5.22)	0.89(0.78,1.01)
Sao Tome and Principe	male	0.41(0.07,0.99)	1.09(0.19,2.59)	1.64	1.60(0.26,3.86)	2.75(0.48,6.54)	2.05(1.96,2.15)

Saudi Arabia	male	36.87(5.87,90.83)	143.55(26.36,326.49)	2.89	1.34(0.22,3.23)	1.80(0.33,4.03)	0.92(0.73,1.10)
Senegal	male	20.15(3.37,51.47)	67.54(11.52,168.38)	2.35	1.40(0.23,3.54)	2.20(0.38,5.45)	2.24(1.87,2.60)
Serbia	male	265.26(42.82,634.78)	642.10(112.97,1533.26)	1.42	4.88(0.79,11.71)	8.46(1.48,20.25)	2.09(1.84,2.34)
Seychelles	male	0.73(0.13,1.75)	2.17(0.38,4.79)	1.97	3.20(0.54,7.64)	4.94(0.89,10.85)	1.22(0.98,1.46)
Sierra Leone	male	4.58(0.66,11.98)	12.56(1.91,31.51)	1.74	0.50(0.07,1.30)	0.85(0.13,2.12)	2.32(2.10,2.54)
Singapore	male	54.94(9.18,125.53)	108.56(18.50,248.07)	0.98	6.26(1.05,14.34)	3.23(0.55,7.37)	-2.52(-2.73,-2.31)
Slovakia	male	126.39(20.31,308.29)	172.14(28.64,434.60)	0.36	4.92(0.79,12.02)	4.33(0.72,10.87)	-0.59(-0.83,-0.36)
Slovenia	male	44.94(7.18,111.60)	85.16(14.74,204.00)	0.90	4.78(0.77,11.75)	4.42(0.76,10.57)	-0.87(-1.27,-0.47)
Solomon Islands	male	2.43(0.33,7.15)	10.62(1.72,30.32)	3.37	3.58(0.55,10.50)	7.81(1.36,21.56)	2.95(2.79,3.12)
Somalia	male	5.85(0.88,16.26)	17.97(2.49,50.80)	2.07	0.66(0.10,1.77)	0.83(0.12,2.34)	0.93(0.85,1.01)
South Africa	male	189.23(31.27,496.52)	522.33(89.09,1205.08)	1.76	2.42(0.40,6.31)	3.12(0.54,7.11)	0.74(0.29,1.19)
Republic of Korea	male	331.87(54.76,793.27)	1676.26(268.05,3864.37)	4.05	2.93(0.48,6.94)	4.55(0.73,10.53)	0.54(-0.06,1.14)
South Sudan	male	9.21(1.38,24.97)	16.65(2.48,42.95)	0.81	0.82(0.12,2.20)	1.01(0.15,2.59)	0.76(0.68,0.84)
Spain	male	1289.03(223.24,2969.57)	2294.46(402.45,5285.68)	0.78	5.39(0.94,12.44)	5.37(0.93,12.40)	-0.42(-0.66,-0.18)
Sri Lanka	male	46.90(7.95,113.43)	255.10(44.32,629.52)	4.44	0.99(0.17,2.40)	2.37(0.42,5.71)	4.03(3.61,4.45)
Sudan	male	39.63(5.07,132.67)	133.13(20.57,368.64)	2.36	0.88(0.11,2.94)	1.50(0.23,4.20)	1.81(1.75,1.87)
Suriname	male	2.32(0.39,5.40)	8.76(1.61,20.01)	2.78	2.04(0.35,4.75)	3.35(0.62,7.68)	1.90(1.61,2.20)
Eswatini	male	2.19(0.32,5.80)	6.68(1.06,17.85)	2.06	2.13(0.33,5.52)	3.41(0.56,8.88)	1.98(1.60,2.36)
Sweden	male	132.35(21.56,313.79)	215.50(36.49,492.26)	0.63	1.93(0.31,4.58)	2.03(0.34,4.64)	0.27(0.04,0.50)
Switzerland	male	200.47(32.65,477.75)	231.79(38.71,544.58)	0.16	4.57(0.74,10.91)	2.84(0.47,6.67)	-1.49(-1.62,-1.35)
Syrian Arab Republic	male	28.94(4.76,69.65)	108.12(17.77,262.05)	2.74	1.16(0.19,2.80)	1.83(0.30,4.42)	1.52(1.32,1.72)
Taiwan (Province of China)	male	167.21(26.93,398.39)	689.93(117.31,1658.54)	3.13	2.10(0.34,4.98)	3.81(0.65,9.15)	1.85(1.47,2.24)

Tajikistan	male	15.04(2.30,36.77)	31.98(5.29,77.72)	1.13	1.22(0.19,2.98)	1.60(0.26,3.92)	1.95(1.27,2.62)
United Republic of Tanzania	male	23.54(3.52,59.92)	91.82(14.16,253.51)	2.90	0.54(0.08,1.40)	0.94(0.15,2.52)	2.30(2.07,2.54)
Thailand	male	347.04(55.32,833.33)	1326.74(219.83,3275.83)	2.82	2.49(0.40,5.97)	3.04(0.51,7.47)	0.19(0.00,0.38)
Bahamas	male	1.62(0.26,3.80)	4.49(0.78,10.46)	1.78	2.69(0.44,6.31)	2.76(0.48,6.35)	-0.01(-0.10,0.09)
Gambia	male	0.69(0.10,1.74)	3.32(0.54,7.93)	3.85	0.47(0.07,1.17)	0.84(0.14,2.02)	1.97(1.78,2.17)
Timor-Leste	male	1.41(0.21,3.59)	10.93(1.86,25.85)	6.78	1.38(0.21,3.46)	2.98(0.52,6.95)	2.61(2.36,2.85)
Togo	male	3.25(0.49,8.34)	14.24(2.21,36.65)	3.38	0.68(0.10,1.75)	1.25(0.20,3.14)	2.38(2.17,2.60)
Tonga	male	1.11(0.19,2.69)	2.46(0.47,5.65)	1.22	4.69(0.79,11.34)	7.20(1.37,16.29)	1.51(1.19,1.84)
Trinidad and Tobago	male	10.02(1.82,22.52)	23.13(4.17,53.89)	1.31	2.65(0.49,5.95)	2.61(0.48,6.03)	-0.24(-0.38,-0.11)
Tunisia	male	82.31(13.35,201.25)	329.12(58.28,802.94)	3.00	3.38(0.55,8.11)	5.56(1.00,13.44)	1.72(1.46,1.98)
Turkey	male	726.75(116.72,1798.81)	2265.62(383.98,5411.79)	2.12	4.50(0.72,11.09)	5.62(0.95,13.28)	2.92(1.73,4.13)
Turkmenistan	male	9.43(1.49,23.08)	19.31(3.22,47.06)	1.05	1.14(0.18,2.79)	1.15(0.19,2.80)	-0.33(-0.89,0.23)
Uganda	male	17.19(2.62,42.42)	50.08(8.12,119.29)	1.91	0.64(0.10,1.57)	1.02(0.17,2.42)	1.58(1.40,1.77)
Ukraine	male	839.49(131.99,2041.63)	746.35(128.08,1854.63)	-0.11	3.16(0.50,7.66)	2.54(0.44,6.25)	-1.73(-2.07,-1.40)
United Arab Emirates	male	5.25(0.89,12.72)	60.15(11.12,139.38)	10.45	3.88(0.67,9.21)	4.53(0.89,10.34)	0.31(-0.21,0.83)
United Kingdom	male	2358.33(386.93,5488.17)	3196.50(569.35,7042.12)	0.36	6.08(1.00,14.17)	5.28(0.94,11.66)	-0.32(-0.51,-0.13)
United States of America	male	9449.11(1572.83,21683.25)	16822.34(3053.46,36256.98)	0.78	6.90(1.15,15.85)	6.46(1.17,13.94)	0.44(0.11,0.77)
Uruguay	male	35.83(5.50,89.05)	104.68(17.38,243.96)	1.92	2.06(0.32,5.14)	4.57(0.76,10.64)	2.77(2.20,3.34)
Uzbekistan	male	52.43(8.29,129.94)	155.66(24.93,361.28)	1.97	1.11(0.18,2.74)	1.85(0.30,4.24)	1.90(1.79,2.01)
Vanuatu	male	0.95(0.14,2.58)	4.54(0.75,11.29)	3.80	3.02(0.46,8.20)	5.50(0.92,13.58)	2.13(2.04,2.21)

Venezuela (Bolivarian Republic of)	male	96.18(15.74,220.33)	412.92(72.13,1000.57)	3.29	2.32(0.38,5.28)	3.17(0.55,7.60)	1.26(0.86,1.66)
Viet nam	male	324.09(50.15,837.81)	1385.20(224.75,3352.54)	3.27	2.07(0.32,5.28)	4.08(0.68,9.83)	2.28(2.22,2.35)
Virginia	male	232.25(37.95,544.24)	434.64(80.55,1027.36)	0.87	7.84(1.29,18.40)	6.62(1.23,15.66)	-0.09(-0.40,0.21)
Yemen	male	19.14(2.74,56.49)	81.04(12.94,219.25)	3.24	0.98(0.15,2.84)	1.44(0.24,3.83)	1.56(1.41,1.70)
Zambia	male	11.00(1.69,27.72)	33.85(5.58,83.96)	2.08	0.87(0.13,2.15)	1.34(0.22,3.28)	1.44(1.24,1.65)
Zimbabwe	male	23.03(3.74,55.17)	45.90(7.70,110.05)	0.99	1.34(0.22,3.21)	1.82(0.31,4.33)	0.96(0.80,1.11)
Monaco	male	1.08(0.16,2.68)	3.45(0.60,8.14)	2.19	3.54(0.52,8.85)	7.56(1.30,17.98)	2.97(2.59,3.36)
San Marino	male	0.54(0.08,1.33)	1.43(0.24,3.75)	1.65	3.56(0.55,8.73)	4.59(0.75,11.95)	0.99(0.94,1.03)
Saint Kitts and Nevis	male	0.33(0.06,0.75)	0.62(0.11,1.40)	0.89	1.98(0.34,4.50)	2.17(0.38,4.80)	-0.06(-0.24,0.13)
Cook Islands	male	0.37(0.06,0.87)	0.97(0.18,2.17)	1.62	6.35(1.06,14.74)	8.01(1.45,17.69)	0.64(0.42,0.85)
Nauru	male	0.08(0.01,0.22)	0.12(0.02,0.32)	0.48	5.65(0.85,14.29)	8.71(1.46,21.30)	1.46(1.38,1.53)
Niue	male	0.05(0.01,0.12)	0.08(0.02,0.19)	0.57	6.01(1.03,13.96)	8.97(1.71,19.79)	1.43(1.28,1.58)
Palau	male	0.23(0.04,0.55)	0.75(0.13,1.66)	2.21	5.34(0.89,12.54)	7.79(1.43,17.20)	1.32(1.19,1.46)
Tokelau	male	0.02(0.00,0.05)	0.03(0.01,0.07)	0.57	2.88(0.48,7.14)	4.56(0.83,10.97)	1.75(1.66,1.85)
Tuvalu	male	0.10(0.02,0.28)	0.26(0.04,0.61)	1.49	3.95(0.65,10.79)	5.83(0.99,13.66)	1.38(1.31,1.45)

**Table S3.** DALYs and age-standardized DALY of TBL cancer attributed to HFPG in 1990 and 2019, and its temporal trends from 1990 to 2019.							
**Nation**	**Sex**	**DALY Cases No. (95% UI)**		**Change in absolute number (%)**	**Age-standardized DALY rate per 100,000 No. (95% UI)**		**1990-2019 EAPC No. (95%CI)**
		**1990**	**2019**		**1990**	**2019**	
Afghanistan	both	1587.08(247.91,4825.30)	3976.20(749.60,10571.56)	1.51	21.65(3.41,65.64)	33.09(6.61,86.48)	1.62(1.53,1.72)
Albania	both	540.87(99.18,1304.66)	1499.84(277.62,3808.83)	1.77	26.27(4.85,63.73)	33.79(6.26,85.50)	0.64(0.40,0.88)
Algeria	both	2216.72(430.71,5415.11)	8653.91(1907.42,19579.64)	2.90	18.52(3.56,45.06)	26.21(5.79,59.29)	1.37(1.06,1.69)
American Samoa	both	21.93(4.82,47.09)	62.25(16.43,124.61)	1.84	98.30(21.87,208.92)	127.46(34.20,253.30)	0.94(0.83,1.06)
Andorra	both	26.06(4.52,66.18)	80.11(15.64,185.12)	2.07	45.52(7.98,115.21)	57.78(11.29,133.91)	0.75(0.71,0.80)
Angola	both	928.38(153.11,2442.94)	3220.36(632.75,7573.97)	2.47	23.75(3.99,61.45)	29.04(5.83,67.60)	0.77(0.59,0.95)
Antigua and Barbuda	both	10.43(2.17,23.24)	26.42(6.35,57.34)	1.53	19.97(4.11,44.67)	25.90(6.20,56.04)	0.95(0.69,1.20)
Argentina	both	12662.29(2444.85,29446.66)	27046.33(6077.54,59085.63)	1.14	38.22(7.40,88.91)	50.36(11.28,110.66)	0.62(0.48,0.76)
Armenia	both	1277.78(243.91,3015.05)	2640.66(518.56,6202.11)	1.07	42.77(8.32,100.52)	60.99(11.99,142.60)	1.07(0.77,1.38)
Australia	both	6188.41(1236.01,14211.94)	14436.47(3307.50,31936.45)	1.33	30.95(6.22,70.97)	34.13(7.81,76.20)	0.14(-0.07,0.34)
Austria	both	3126.61(629.34,7277.68)	7007.55(1594.89,15532.53)	1.24	26.82(5.30,62.31)	41.65(9.45,92.91)	1.76(1.54,1.98)
Azerbaijan	both	1473.32(280.63,3602.04)	4637.31(917.72,10915.46)	2.15	27.08(5.27,65.64)	44.25(9.08,102.30)	2.15(1.76,2.54)
Bahrain	both	190.92(39.89,418.92)	665.15(159.13,1394.31)	2.48	131.69(28.41,280.85)	87.18(21.41,178.88)	-2.05(-2.51,-1.58)
Bangladesh	both	5079.32(869.17,12886.16)	15895.15(2993.82,43608.10)	2.13	11.10(1.87,28.37)	12.15(2.29,33.27)	0.52(0.32,0.72)
Barbados	both	52.81(12.29,117.82)	119.16(28.32,262.68)	1.26	18.25(4.22,40.96)	23.73(5.65,52.38)	0.63(0.48,0.78)
Belarus	both	4067.32(740.82,9866.17)	3967.36(713.58,9717.69)	-0.02	30.22(5.48,73.24)	24.65(4.44,60.57)	-1.67(-2.05,-1.28)
Belgium	both	8930.67(1665.23,21157.58)	13059.64(2921.72,28950.46)	0.46	57.79(10.71,137.08)	60.23(13.36,134.17)	0.15(-0.04,0.33)

Belize	both	12.06(2.62,27.62)	76.24(16.81,173.61)	5.32	13.25(2.88,30.34)	27.57(6.11,62.18)	2.24(1.69,2.79)
Benin	both	212.97(40.45,516.35)	811.17(171.66,1878.90)	2.81	11.15(2.11,26.86)	18.29(3.89,42.08)	1.89(1.80,1.98)
Bermuda	both	33.67(6.75,77.56)	56.08(11.90,127.30)	0.67	53.90(10.81,124.12)	42.72(9.05,97.58)	-0.80(-0.94,-0.65)
Bhutan	both	16.65(3.19,44.30)	78.38(16.66,190.30)	3.71	6.78(1.35,17.54)	14.14(3.00,34.07)	2.75(2.67,2.83)
Bolivia (Plurinational State of)	both	534.27(105.43,1363.21)	2227.29(458.97,5712.22)	3.17	17.30(3.42,43.69)	25.79(5.42,65.35)	1.25(1.18,1.32)
Bosnia and Herzegovina	both	2392.26(468.12,5615.48)	6816.94(1398.65,15891.73)	1.85	55.15(10.97,128.37)	109.84(22.42,255.38)	2.65(2.36,2.93)
Botswana	both	145.64(30.27,356.56)	636.26(131.34,1509.02)	3.37	25.58(5.37,61.95)	47.98(10.18,112.38)	2.06(1.69,2.43)
Brazil	both	26997.30(6083.64,60261.71)	69068.68(16323.19,150751.52)	1.56	30.32(6.85,67.30)	29.07(6.89,63.34)	0.07(-0.02,0.16)
Brunei Darussalam	both	146.15(36.78,309.73)	421.75(111.13,860.50)	1.89	170.60(44.42,359.41)	159.28(43.13,319.75)	0.17(-0.05,0.38)
Bulgaria	both	5073.38(984.44,11802.55)	9519.46(1779.92,22991.34)	0.88	38.67(7.47,89.72)	70.30(12.92,171.30)	3.50(2.93,4.07)
Burkina Faso	both	390.27(72.76,959.46)	1349.08(269.82,3285.10)	2.46	9.32(1.71,22.75)	16.06(3.27,38.83)	2.03(1.88,2.19)
Burundi	both	206.47(38.08,509.61)	418.05(76.73,1043.33)	1.02	9.01(1.67,22.10)	9.85(1.84,24.56)	0.11(0.01,0.21)
Cambodia	both	992.31(190.57,2474.86)	6197.93(1307.22,14125.85)	5.25	23.04(4.42,57.39)	53.77(11.45,122.21)	2.95(2.74,3.15)
Cameroon	both	465.01(87.69,1138.56)	2448.14(502.63,5951.17)	4.26	11.25(2.16,27.40)	22.65(4.66,55.35)	2.61(2.21,3.01)
Canada	both	14002.65(3008.40,31876.57)	35000.83(7982.68,78148.22)	1.50	42.21(9.07,96.08)	50.10(11.42,111.58)	0.24(0.10,0.38)
Cabo Verde	both	27.33(5.92,63.17)	135.94(32.13,312.35)	3.97	11.59(2.50,26.83)	34.61(8.09,79.63)	3.26(2.95,3.56)
Central African Republic	both	300.03(42.20,962.68)	654.62(100.97,2084.46)	1.18	24.38(3.65,76.41)	28.52(4.65,87.35)	0.59(0.46,0.72)
Chad	both	242.80(45.27,612.12)	973.82(185.81,2408.06)	3.01	8.74(1.64,21.94)	19.01(3.60,46.74)	2.97(2.70,3.23)
Chile	both	2670.49(563.80,6065.16)	8792.59(2105.96,18707.88)	2.29	26.72(5.65,60.65)	36.13(8.65,76.90)	1.31(1.16,1.47)
China	both	345568.91(72604.95,813013.91)	1107653.71(242106.53,2548448.85)	2.21	39.99(8.53,94.01)	53.77(11.81,123.10)	1.27(0.90,1.63)
Colombia	both	5546.54(1292.14,12204.75)	13931.16(3255.31,32205.42)	1.51	32.97(7.66,72.14)	26.58(6.22,61.45)	-1.19(-1.36,-1.02)
Comoros	both	15.82(2.75,39.98)	42.95(8.59,104.35)	1.72	7.29(1.28,18.30)	9.09(1.83,21.90)	0.65(0.53,0.77)
Congo	both	339.81(55.30,923.91)	877.10(177.40,2109.89)	1.58	31.01(5.16,83.61)	34.18(7.10,80.13)	0.16(-0.04,0.36)
Costa Rica	both	376.17(82.63,829.77)	1175.89(257.68,2707.29)	2.13	22.34(4.95,49.09)	23.08(5.06,53.03)	-0.19(-0.39,0.02)

Côte d'Ivoire	both	469.14(85.34,1187.67)	2019.10(410.20,4849.26)	3.30	13.13(2.41,33.04)	21.55(4.47,50.83)	1.65(1.49,1.80)
Croatia	both	4486.49(880.55,10252.30)	6631.74(1307.00,15364.92)	0.48	67.00(13.19,152.87)	77.67(15.20,181.74)	0.79(0.52,1.07)
Cuba	both	6620.00(1420.13,14714.84)	15515.30(3535.01,34456.17)	1.34	64.22(13.78,142.91)	81.83(18.59,181.96)	1.03(0.84,1.22)
Cyprus	both	389.76(80.79,866.21)	1170.66(256.01,2518.05)	2.00	45.23(9.29,100.81)	57.88(12.69,124.89)	0.99(0.77,1.20)
Czechia	both	13283.46(2569.95,30037.40)	20164.59(4563.77,44623.90)	0.52	96.33(18.49,218.51)	95.11(21.38,211.82)	0.06(-0.18,0.30)
Democratic Republic of the Congo	both	3510.73(521.72,13231.96)	8732.36(1393.99,29228.79)	1.49	22.41(3.35,85.78)	24.64(3.98,80.82)	0.17(-0.01,0.35)
Denmark	both	3260.21(696.06,7542.30)	6237.80(1442.49,13747.35)	0.91	41.71(8.93,96.44)	53.60(12.47,118.10)	0.85(0.65,1.04)
Djibouti	both	11.42(1.95,30.09)	86.82(15.26,248.14)	6.60	9.13(1.59,23.91)	15.84(2.88,43.60)	1.92(1.84,2.00)
Dominica	both	24.54(5.39,54.64)	51.09(11.30,112.68)	1.08	34.28(7.45,76.46)	56.40(12.49,124.01)	1.86(1.77,1.95)
Dominican Republic	both	360.29(75.55,844.76)	2118.11(446.24,5198.79)	4.88	9.91(2.07,23.12)	23.00(4.86,56.40)	3.57(3.33,3.81)
Ecuador	both	547.25(120.40,1238.88)	2966.65(693.79,6622.29)	4.42	10.74(2.38,24.24)	20.09(4.72,44.81)	2.37(2.05,2.69)
Egypt	both	1794.33(388.67,4213.52)	12939.09(2721.73,31171.62)	6.21	6.03(1.32,14.13)	19.43(4.16,46.31)	4.34(4.22,4.47)
El Salvador	both	372.17(85.55,831.39)	1461.02(349.42,3379.58)	2.93	12.86(2.96,28.77)	25.06(6.00,58.08)	2.23(2.01,2.46)
Equatorial Guinea	both	44.70(6.69,133.53)	159.76(31.73,412.63)	2.57	21.85(3.38,64.48)	35.06(7.23,89.00)	2.10(1.89,2.30)
Eritrea	both	68.03(12.60,180.18)	312.81(63.61,756.14)	3.60	6.92(1.31,17.75)	12.10(2.50,28.53)	1.72(1.48,1.97)
Estonia	both	857.16(164.02,2029.84)	992.77(209.46,2324.49)	0.16	41.19(7.87,97.70)	39.58(8.19,93.64)	-0.16(-0.45,0.13)
Ethiopia	both	1488.19(225.59,4501.92)	2410.86(455.72,6078.01)	0.62	7.80(1.24,23.24)	6.39(1.21,15.98)	-1.19(-1.35,-1.03)
Micronesia (Federated States of)	both	24.23(4.65,60.13)	80.78(16.67,203.58)	2.33	51.25(10.02,128.10)	109.64(23.63,270.83)	2.67(2.36,2.99)
Fiji	both	107.36(23.37,237.07)	349.46(90.76,744.48)	2.26	31.37(6.92,68.36)	46.17(12.13,97.64)	1.16(1.06,1.25)
Finland	both	3283.52(702.54,7436.74)	5320.35(1277.97,11423.12)	0.62	45.69(9.72,103.60)	43.31(10.31,93.78)	0.12(-0.06,0.30)
France	both	20206.61(3583.01,48876.35)	44801.61(9228.33,104694.08)	1.22	25.45(4.48,61.70)	36.26(7.48,85.05)	1.31(1.16,1.47)
Gabon	both	200.58(32.81,576.92)	499.90(98.48,1292.46)	1.49	34.72(5.75,98.30)	47.64(9.74,121.97)	1.07(0.96,1.18)
Georgia	both	2238.63(427.26,5305.13)	4473.39(906.63,10444.40)	1.00	33.99(6.51,79.62)	77.25(15.64,180.96)	4.16(3.49,4.84)

Germany	both	65081.20(13174.63,148272.64)	127543.22(29939.18,273507.02)	0.96	52.38(10.54,119.68)	71.45(16.52,154.52)	0.88(0.78,0.99)
Ghana	both	518.89(111.02,1236.26)	2451.28(531.64,5651.63)	3.72	8.83(1.88,20.67)	16.22(3.63,37.61)	2.42(2.12,2.71)
Greece	both	7429.45(1421.40,17623.64)	15136.86(3154.15,34345.54)	1.04	47.42(9.06,112.75)	70.38(14.59,160.24)	1.33(1.24,1.43)
Greenland	both	24.79(4.99,57.74)	90.34(19.00,209.19)	2.64	73.17(14.84,171.53)	126.78(26.60,291.10)	1.58(1.38,1.77)
Grenada	both	21.98(4.90,49.90)	51.31(12.07,109.50)	1.33	31.41(6.98,71.68)	44.49(10.56,93.95)	1.48(1.28,1.69)
Guam	both	46.00(10.03,106.84)	141.40(33.09,313.69)	2.07	62.84(13.49,146.53)	73.61(17.21,162.67)	0.57(0.25,0.89)
Guatemala	both	497.99(112.59,1118.74)	2344.08(541.13,5311.65)	3.71	14.33(3.27,32.26)	21.58(5.00,48.65)	1.06(0.66,1.47)
Guinea	both	248.08(45.45,601.74)	781.26(155.30,1889.06)	2.15	7.63(1.40,18.55)	14.87(3.00,35.90)	2.42(2.29,2.55)
Guinea-Bissau	both	61.12(10.34,166.90)	143.06(28.51,367.85)	1.34	15.55(2.65,42.10)	21.48(4.30,54.73)	1.36(1.25,1.48)
Guyana	both	75.33(17.01,167.77)	193.55(46.56,435.18)	1.57	19.95(4.52,44.11)	29.74(7.21,66.84)	1.50(1.34,1.66)
Haiti	both	872.02(167.33,2487.19)	2209.38(461.33,5780.56)	1.53	26.27(5.08,74.22)	31.85(6.78,81.04)	0.79(0.64,0.95)
Honduras	both	546.26(128.90,1247.02)	3713.14(817.53,9242.43)	5.80	26.96(6.40,61.16)	61.62(13.67,152.65)	3.01(2.85,3.18)
Hungary	both	10828.83(2162.88,25214.26)	20891.43(4582.87,47138.45)	0.93	73.10(14.50,170.69)	112.88(24.33,256.97)	1.48(0.96,1.99)
Iceland	both	85.73(18.75,195.68)	233.56(55.60,520.65)	1.72	30.25(6.58,69.33)	42.92(10.18,95.94)	1.12(0.95,1.30)
India	both	44743.32(8676.93,106444.11)	203815.36(44569.78,471553.48)	3.56	9.99(1.95,23.64)	17.63(3.91,40.55)	1.63(1.50,1.75)
Indonesia	both	17008.61(3311.44,40070.28)	78321.55(15822.90,193158.91)	3.60	17.87(3.48,42.15)	36.97(7.52,89.76)	2.40(2.26,2.55)
Iran (Islamic Republic of)	both	3689.47(701.41,9117.66)	19063.09(4409.22,41272.32)	4.17	14.27(2.77,35.02)	26.91(6.21,57.83)	2.52(2.32,2.72)
Iraq	both	2496.05(491.78,5936.98)	12254.93(2701.50,27861.97)	3.91	32.73(6.48,77.74)	54.86(12.05,123.27)	1.96(1.69,2.22)
Ireland	both	1136.46(236.54,2671.67)	3644.90(843.07,7929.76)	2.21	26.84(5.57,63.31)	48.05(11.10,104.99)	2.12(2.00,2.23)
Israel	both	1431.01(316.89,3213.83)	4549.24(1073.96,9932.77)	2.18	29.10(6.42,65.95)	39.60(9.34,86.86)	0.81(0.40,1.21)
Italy	both	53044.58(10376.00,122572.12)	75324.06(16784.45,162218.62)	0.42	59.05(11.48,136.48)	55.41(12.48,120.25)	-0.09(-0.59,0.41)
Jamaica	both	461.68(101.10,1038.96)	1610.23(337.99,3735.66)	2.49	26.44(5.79,59.89)	54.92(11.55,127.21)	2.51(1.87,3.14)
Japan	both	53584.24(10836.09,122388.29)	96488.46(19599.59,220176.42)	0.80	30.98(6.29,70.67)	26.74(5.35,61.36)	-0.83(-0.97,-0.70)
Jordan	both	354.61(69.99,840.45)	2593.41(558.13,5807.68)	6.31	26.94(5.35,63.90)	40.68(8.82,89.84)	1.69(1.44,1.94)
Kazakhstan	both	6717.46(1369.05,15754.05)	7966.78(1717.43,18266.72)	0.19	50.23(10.42,116.96)	43.77(9.52,99.74)	-0.45(-0.60,-0.30)

Kenya	both	341.17(61.46,849.60)	1644.48(326.26,3959.88)	3.82	4.50(0.82,11.15)	7.99(1.58,18.80)	2.05(1.82,2.28)
Kiribati	both	18.36(3.53,44.60)	57.02(12.40,130.59)	2.11	46.72(9.15,112.75)	76.16(16.61,169.31)	1.68(1.50,1.85)
Kuwait	both	172.10(36.25,387.26)	754.80(168.00,1648.93)	3.39	30.89(6.63,69.21)	34.54(7.69,74.44)	0.42(-0.09,0.92)
Kyrgyzstan	both	705.32(134.58,1681.96)	708.16(140.69,1662.51)	0.00	22.59(4.36,53.84)	15.05(3.02,35.07)	-1.62(-2.07,-1.18)
Lao People's Democratic Republic	both	760.87(137.39,2001.07)	2183.58(472.30,5093.13)	1.87	36.51(6.78,96.14)	52.59(11.50,121.76)	0.95(0.83,1.07)
Latvia	both	1206.62(232.26,2876.50)	1353.15(277.98,3139.68)	0.12	33.20(6.38,79.28)	36.37(7.38,85.04)	0.39(0.01,0.78)
Lebanon	both	893.01(165.10,2237.38)	3889.88(918.37,8555.58)	3.36	38.33(7.11,95.44)	75.00(17.80,165.20)	3.08(2.77,3.40)
Lesotho	both	154.83(29.45,404.71)	463.14(93.86,1145.75)	1.99	15.64(3.01,40.62)	35.90(7.48,87.62)	3.50(3.26,3.74)
Liberia	both	147.63(27.71,352.94)	328.98(63.83,781.24)	1.23	13.33(2.52,31.74)	18.31(3.60,42.95)	1.79(1.42,2.17)
Libya	both	702.63(117.25,1748.12)	3046.16(582.27,6964.56)	3.34	38.70(6.54,96.77)	61.98(12.04,139.44)	1.73(1.51,1.95)
Lithuania	both	1432.48(265.81,3415.41)	1571.63(298.14,3780.75)	0.10	31.30(5.81,74.36)	29.25(5.47,70.73)	-0.18(-0.47,0.11)
Luxembourg	both	193.10(36.23,460.97)	704.90(161.12,1544.86)	2.65	35.28(6.65,84.34)	72.53(16.51,158.82)	2.74(2.56,2.92)
North Macedonia	both	968.05(184.27,2253.04)	3767.99(759.83,8891.21)	2.89	48.93(9.33,113.87)	110.21(22.18,260.05)	2.98(2.58,3.39)
Madagascar	both	317.97(62.30,774.04)	833.06(166.86,2096.30)	1.62	6.49(1.27,15.78)	8.16(1.60,20.07)	0.68(0.59,0.77)
Malawi	both	243.50(48.80,584.28)	695.46(145.23,1607.96)	1.86	6.75(1.36,15.99)	10.12(2.10,23.12)	1.51(1.18,1.85)
Malaysia	both	2491.51(520.05,5741.91)	11793.45(2514.47,27743.70)	3.73	28.79(6.06,66.09)	45.18(9.75,105.67)	1.50(1.12,1.88)
Maldives	both	14.12(2.58,36.83)	46.11(9.94,104.91)	2.26	18.66(3.37,47.38)	17.81(3.83,40.47)	-0.79(-1.03,-0.55)
Mali	both	238.83(48.04,570.61)	850.05(179.55,2040.18)	2.56	5.99(1.22,14.27)	10.59(2.23,25.25)	2.05(1.99,2.12)
Malta	both	209.84(40.03,489.45)	434.11(90.24,948.47)	1.07	47.99(9.13,112.11)	46.27(9.59,102.02)	0.01(-0.09,0.11)
Marshall Islands	both	14.87(2.87,39.23)	51.95(10.94,130.56)	2.49	90.41(17.45,238.89)	144.79(31.98,360.43)	1.83(1.69,1.97)
Mauritania	both	83.46(16.93,203.29)	234.32(49.69,556.90)	1.81	8.50(1.74,20.57)	12.00(2.56,28.30)	1.08(0.89,1.27)
Mauritius	both	219.20(45.67,485.90)	740.44(174.49,1585.10)	2.38	29.76(6.24,65.78)	41.33(9.83,88.34)	1.39(1.12,1.65)
Mexico	both	15995.96(3983.53,33813.57)	32273.83(7873.76,68977.98)	1.02	39.27(9.82,82.70)	27.93(6.80,59.67)	-1.65(-1.93,-1.38)
Republic of Moldova	both	1719.64(340.32,4060.76)	1746.55(348.78,4102.20)	0.02	36.38(7.25,85.56)	29.43(5.87,69.49)	0.04(-0.37,0.45)

Mongolia	both	238.95(46.36,593.62)	595.55(109.27,1482.29)	1.49	23.21(4.47,57.05)	27.18(5.23,65.82)	0.19(0.05,0.33)
Montenegro	both	495.39(97.86,1132.80)	1362.77(282.86,3133.83)	1.75	77.64(15.49,176.68)	134.17(27.72,309.67)	1.97(1.72,2.22)
Morocco	both	2853.62(498.43,7040.91)	13975.27(2762.46,33660.60)	3.90	20.39(3.60,49.85)	42.88(8.60,103.36)	2.52(2.37,2.66)
Mozambique	both	321.71(61.33,768.56)	1361.61(267.81,3291.00)	3.23	5.91(1.14,13.95)	13.33(2.63,32.12)	3.39(3.17,3.61)
Myanmar	both	7980.80(1559.63,21124.02)	21756.25(4863.52,51454.24)	1.73	34.39(6.90,91.28)	48.17(10.81,114.50)	1.03(0.95,1.12)
Namibia	both	67.53(14.06,163.26)	194.87(42.69,445.45)	1.89	9.29(1.96,22.41)	14.46(3.20,32.62)	1.66(1.51,1.81)
Nepal	both	790.21(143.01,2068.68)	3526.38(727.18,8337.12)	3.46	8.45(1.50,22.13)	15.76(3.28,37.07)	1.99(1.79,2.19)
Netherlands	both	11444.85(2129.31,26666.82)	18324.06(4216.45,40874.99)	0.60	57.17(10.63,133.96)	53.41(12.34,119.45)	-0.33(-0.51,-0.15)
New Zealand	both	1291.51(277.51,2999.75)	2713.42(635.33,6032.85)	1.10	32.72(7.03,75.63)	34.69(8.08,77.08)	-0.59(-1.05,-0.12)
Nicaragua	both	224.41(49.29,501.77)	928.36(216.33,2040.66)	3.14	15.60(3.42,34.58)	21.89(5.15,47.89)	1.02(0.89,1.15)
Niger	both	118.40(19.63,318.41)	704.91(128.74,1839.83)	4.95	4.70(0.79,12.48)	10.10(1.86,26.41)	2.87(2.57,3.17)
Nigeria	both	2478.45(456.27,6416.89)	7017.54(1495.69,16948.50)	1.83	6.02(1.10,15.52)	9.41(1.98,22.77)	1.64(1.55,1.73)
Democratic People's Republic of Korea	both	4525.71(921.20,11516.22)	13680.69(2841.19,31456.91)	2.02	27.00(5.68,67.62)	41.83(8.74,96.28)	1.57(1.52,1.62)
Northern Mariana Islands	both	17.73(3.40,41.22)	70.68(15.99,154.23)	2.99	109.18(21.76,249.32)	131.45(29.86,281.69)	0.55(0.42,0.68)
Norway	both	2451.94(542.15,5514.46)	4329.60(1019.72,9413.73)	0.77	37.10(8.15,83.87)	45.29(10.63,98.42)	0.45(0.21,0.69)
Oman	both	103.77(19.61,262.02)	399.91(85.62,926.61)	2.85	16.48(3.19,41.24)	27.43(6.13,60.71)	2.01(1.71,2.31)
Pakistan	both	11602.66(2010.49,28037.26)	41427.26(8173.78,103751.52)	2.57	20.41(3.52,49.35)	36.69(7.32,91.08)	2.14(1.79,2.48)
Palestine	both	326.82(60.81,797.63)	1662.79(359.00,3631.98)	4.09	38.50(7.12,93.23)	72.91(16.22,157.62)	2.40(2.12,2.69)
Panama	both	391.07(81.27,884.91)	1103.70(241.61,2533.29)	1.82	26.83(5.56,60.44)	26.92(5.89,61.61)	-0.13(-0.25,-0.01)
Papua New Guinea	both	736.85(136.90,1901.07)	3160.55(614.21,7808.60)	3.29	39.51(7.18,102.57)	65.90(12.99,160.15)	1.86(1.77,1.94)
Paraguay	both	273.55(58.83,636.84)	1708.31(335.26,4028.55)	5.25	12.64(2.72,29.40)	31.36(6.19,73.77)	2.78(2.42,3.15)
Peru	both	1558.04(322.75,3655.03)	4425.85(997.66,10554.33)	1.84	13.68(2.81,32.18)	14.09(3.18,33.66)	0.01(-0.38,0.41)
Philippines	both	8721.93(1848.76,20216.55)	22316.99(4724.80,51980.79)	1.56	30.81(6.67,71.31)	29.19(6.25,67.37)	-1.01(-1.30,-0.71)
Poland	both	33241.78(6367.04,76554.64)	68239.34(14705.96,149981.30)	1.05	74.30(14.26,171.60)	97.29(20.88,214.40)	0.98(0.86,1.10)

Portugal	both	4701.78(931.63,10627.08)	11066.78(2448.78,24085.08)	1.35	32.70(6.47,74.17)	50.37(11.15,110.15)	1.68(1.31,2.04)
Puerto Rico	both	1391.31(325.25,3011.60)	2356.02(557.42,5241.44)	0.69	37.95(8.83,82.31)	33.58(7.87,74.98)	-0.63(-0.83,-0.44)
Qatar	both	52.98(10.39,126.28)	625.96(141.48,1377.21)	10.81	62.24(13.18,141.49)	87.34(20.72,180.57)	1.34(0.80,1.87)
Romania	both	8411.99(1539.37,19849.67)	18391.20(3694.38,42722.04)	1.19	28.37(5.21,66.85)	52.41(10.50,122.20)	1.96(1.77,2.14)
Russian Federation	both	51396.18(9634.56,122963.76)	62019.43(12169.68,147003.50)	0.21	27.07(5.10,64.59)	25.74(5.05,61.11)	-0.60(-0.90,-0.31)
Rwanda	both	270.61(52.80,682.19)	604.89(116.98,1575.36)	1.24	9.64(1.84,24.36)	10.78(2.12,27.97)	-0.14(-0.44,0.15)
Saint Lucia	both	32.65(7.95,70.40)	87.38(21.48,187.15)	1.68	37.27(9.06,80.47)	40.24(9.90,86.09)	0.11(-0.12,0.33)
Saint Vincent and the Grenadines	both	16.14(3.80,34.79)	44.46(10.87,95.89)	1.75	22.32(5.26,48.22)	32.20(7.92,69.50)	1.10(0.93,1.28)
Samoa	both	22.25(4.77,50.31)	54.84(12.44,125.19)	1.47	25.08(5.40,56.29)	37.23(8.54,85.15)	1.29(1.19,1.40)
Sao Tome and Principe	both	10.66(2.12,24.88)	31.64(7.06,72.27)	1.97	16.78(3.33,38.99)	33.31(7.57,75.40)	2.48(2.36,2.60)
Saudi Arabia	both	998.13(196.86,2405.78)	4780.93(1048.85,10653.67)	3.79	18.10(3.59,44.05)	28.11(6.24,61.28)	1.31(1.13,1.49)
Senegal	both	519.62(101.98,1245.71)	1838.93(398.56,4304.93)	2.54	16.84(3.35,40.01)	26.18(5.65,61.12)	2.15(1.81,2.49)
Serbia	both	8121.22(1569.76,19046.13)	19257.43(4071.48,44081.17)	1.37	65.68(12.89,154.58)	120.40(25.21,279.42)	2.30(2.07,2.53)
Seychelles	both	17.84(3.59,41.20)	61.52(14.28,133.68)	2.45	31.38(6.32,72.82)	57.28(13.66,123.29)	1.79(1.56,2.02)
Sierra Leone	both	108.84(20.36,274.23)	357.05(73.24,872.63)	2.28	5.85(1.09,14.75)	10.82(2.22,26.19)	2.60(2.40,2.81)
Singapore	both	1684.98(369.57,3742.04)	2912.17(675.90,6399.66)	0.73	77.98(17.25,172.27)	37.48(8.72,82.33)	-2.78(-2.97,-2.59)
Slovakia	both	3419.99(618.47,8053.34)	4783.76(924.96,11483.70)	0.40	56.34(10.12,133.39)	50.41(9.69,120.87)	-0.46(-0.72,-0.20)
Slovenia	both	1280.18(230.98,3094.85)	2296.19(470.39,5353.61)	0.79	51.70(9.31,125.13)	55.35(11.28,129.36)	-0.21(-0.56,0.14)
Solomon Islands	both	74.09(10.92,218.56)	342.73(58.90,962.61)	3.63	52.11(7.91,150.56)	106.95(19.44,289.35)	2.65(2.54,2.76)
Somalia	both	166.06(28.04,448.22)	533.66(89.81,1492.98)	2.21	7.19(1.23,19.36)	8.38(1.42,23.60)	0.64(0.58,0.70)
South Africa	both	6156.85(1262.33,15178.34)	18277.79(4153.43,40443.04)	1.97	30.06(6.19,73.85)	41.11(9.49,90.77)	1.02(0.59,1.44)
Republic of Korea	both	10707.53(2253.63,24480.91)	39889.28(8487.18,87802.75)	2.73	34.61(7.42,79.16)	43.95(9.37,97.24)	-0.08(-0.55,0.38)
South Sudan	both	231.65(39.34,615.86)	437.52(80.45,1163.69)	0.89	10.16(1.72,27.21)	12.62(2.38,32.75)	0.76(0.67,0.85)
Spain	both	29988.26(5625.02,68119.93)	53031.68(10787.09,117478.76)	0.77	53.61(10.00,122.02)	60.52(12.21,134.91)	0.00(-0.25,0.24)

Sri Lanka	both	1366.55(287.47,3100.06)	7922.38(1741.56,18922.96)	4.80	13.07(2.77,29.60)	30.01(6.73,70.99)	3.69(3.36,4.02)
Sudan	both	1038.20(174.32,3274.98)	3893.60(775.93,10194.23)	2.75	11.28(1.98,35.30)	21.82(4.28,56.97)	2.30(2.23,2.36)
Suriname	both	72.94(16.48,163.95)	302.67(74.87,657.59)	3.15	28.27(6.39,63.48)	49.43(12.22,106.38)	2.17(1.92,2.43)
Eswatini	both	69.50(13.25,177.40)	235.20(46.48,597.46)	2.38	24.79(4.76,62.64)	41.00(8.27,102.10)	2.10(1.64,2.56)
Sweden	both	3593.17(809.84,8159.07)	6951.58(1683.34,15087.02)	0.93	24.58(5.50,55.96)	33.07(8.02,71.80)	1.21(0.93,1.49)
Switzerland	both	5129.89(1012.58,11743.74)	6494.55(1522.59,14350.43)	0.27	51.12(10.04,117.10)	38.96(9.03,86.35)	-0.65(-0.80,-0.50)
Syrian Arab Republic	both	850.76(179.46,2036.77)	3421.82(737.79,7897.17)	3.02	16.34(3.47,38.75)	27.11(5.94,62.18)	1.63(1.43,1.83)
Taiwan (Province of China)	both	5538.86(1248.98,12581.85)	20394.77(4606.52,46855.27)	2.68	33.88(7.67,76.77)	51.06(11.45,117.09)	1.09(0.79,1.39)
Tajikistan	both	542.78(109.63,1282.90)	1181.88(256.99,2725.97)	1.18	18.71(3.76,44.30)	23.21(5.12,53.97)	1.62(1.03,2.22)
United Republic of Tanzania	both	623.92(115.56,1545.20)	2590.13(507.57,6742.78)	3.15	6.02(1.10,14.83)	11.29(2.22,29.40)	2.62(2.44,2.81)
Thailand	both	12342.50(2741.41,28402.31)	38776.16(8388.18,93736.63)	2.14	35.19(7.80,80.31)	38.01(8.25,91.57)	-0.66(-0.97,-0.36)
Bahamas	both	49.07(10.13,111.61)	147.92(32.36,333.28)	2.01	32.16(6.68,73.17)	36.91(8.13,82.26)	0.53(0.46,0.61)
Gambia	both	19.25(3.69,46.65)	104.25(22.05,242.68)	4.42	5.87(1.15,14.20)	11.67(2.50,26.97)	2.29(2.12,2.47)
Timor-Leste	both	42.30(7.88,106.23)	315.46(64.58,728.65)	6.46	17.10(3.28,42.16)	38.62(8.11,89.22)	2.72(2.44,3.00)
Togo	both	92.83(17.22,225.39)	449.81(92.64,1069.52)	3.85	8.19(1.55,19.78)	13.50(2.76,31.97)	1.79(1.69,1.89)
Tonga	both	30.44(6.18,70.93)	68.24(14.57,152.82)	1.24	55.68(11.23,130.02)	87.75(18.76,196.23)	1.58(1.35,1.81)
Trinidad and Tobago	both	293.23(64.29,632.08)	690.31(146.87,1603.29)	1.35	34.89(7.67,75.07)	35.98(7.66,83.43)	-0.13(-0.25,-0.02)
Tunisia	both	1904.71(331.66,4657.36)	7977.54(1458.77,19531.78)	3.19	37.44(6.56,90.47)	61.89(11.41,151.87)	1.73(1.48,1.98)
Turkey	both	20252.35(3489.62,50368.32)	60921.33(12470.06,143176.07)	2.01	55.37(9.55,134.54)	68.12(14.05,159.52)	2.88(1.61,4.17)
Turkmenistan	both	341.10(66.47,807.29)	694.72(135.84,1654.43)	1.04	16.46(3.22,39.08)	16.34(3.19,38.72)	-0.37(-0.94,0.20)
Uganda	both	455.58(88.82,1103.80)	1639.27(356.40,3724.65)	2.60	7.34(1.43,17.78)	12.44(2.72,28.22)	1.76(1.60,1.92)
Ukraine	both	27057.26(5078.20,63368.47)	21834.53(4231.72,53263.93)	-0.19	36.73(6.91,86.15)	29.21(5.63,71.58)	-1.88(-2.27,-1.49)
United Arab Emirates	both	156.56(31.81,374.25)	2033.79(459.73,4696.34)	11.99	53.04(11.56,123.04)	72.33(16.75,166.59)	1.02(0.41,1.64)
United Kingdom	both	65450.53(14848.54,145693.59)	97188.03(23972.11,203529.99)	0.48	71.40(16.07,159.26)	77.76(19.10,163.38)	0.39(0.22,0.56)

United States of America	both	304121.97(69275.17,662441.38)	533304.48(132870.26,1109806.01)	0.75	98.22(22.35,214.58)	94.43(23.49,196.47)	0.52(0.21,0.84)
Uruguay	both	908.34(160.06,2211.13)	2714.35(548.85,6190.68)	1.99	23.00(4.04,56.01)	52.98(10.64,120.85)	2.95(2.40,3.50)
Uzbekistan	both	1877.26(368.94,4446.58)	5692.34(1185.38,12875.73)	2.03	15.81(3.17,37.25)	24.72(5.30,55.50)	1.44(1.29,1.58)
Vanuatu	both	25.78(4.57,69.58)	133.43(26.84,321.17)	4.18	40.41(7.20,108.19)	76.63(15.43,183.39)	2.16(2.03,2.30)
Venezuela (Bolivarian Republic of)	both	3351.22(759.26,7363.39)	14229.60(3128.61,33801.71)	3.25	35.49(8.10,77.83)	48.22(10.69,113.60)	1.25(0.99,1.51)
Viet nam	both	9337.38(1760.07,23281.05)	43660.36(9026.37,101077.68)	3.68	22.98(4.29,57.14)	47.65(9.95,108.68)	2.53(2.46,2.60)
Virginia	both	7568.34(1690.97,16502.31)	13946.05(3388.32,30595.34)	0.84	107.97(24.12,235.77)	95.92(23.18,209.98)	0.15(-0.15,0.45)
Yemen	both	536.61(90.26,1541.14)	2352.17(451.05,6084.49)	3.38	10.96(1.89,31.41)	18.33(3.59,46.94)	2.03(1.88,2.17)
Zambia	both	301.64(56.35,745.99)	1014.96(210.66,2468.73)	2.36	11.38(2.11,28.21)	16.36(3.44,39.61)	1.05(0.89,1.22)
Zimbabwe	both	752.88(158.26,1732.53)	2133.38(479.35,4737.67)	1.83	18.87(3.99,42.81)	31.00(7.08,68.30)	1.96(1.86,2.06)
Monaco	both	26.73(5.16,64.54)	93.99(21.00,211.95)	2.52	40.79(7.75,99.96)	104.08(23.14,236.77)	3.65(3.18,4.12)
San Marino	both	12.77(2.37,30.55)	34.86(6.77,91.38)	1.73	37.53(6.99,90.45)	57.28(11.04,151.98)	1.70(1.60,1.81)
Saint Kitts and Nevis	both	10.72(2.56,23.27)	21.76(5.04,47.40)	1.03	28.08(6.62,61.18)	31.78(7.45,68.50)	0.20(-0.03,0.42)
Cook Islands	both	9.84(2.00,22.61)	25.28(5.57,54.67)	1.57	79.34(16.22,180.31)	99.66(21.99,216.66)	0.60(0.44,0.76)
Nauru	both	2.88(0.57,7.42)	5.20(1.10,12.95)	0.81	78.82(15.55,199.36)	121.57(26.98,290.75)	1.40(1.24,1.56)
Niue	both	1.63(0.38,3.58)	2.80(0.70,5.92)	0.72	76.19(17.49,167.62)	127.53(31.65,269.46)	1.77(1.61,1.92)
Palau	both	9.50(2.18,21.41)	35.75(8.86,77.24)	2.76	96.20(22.30,216.87)	159.16(39.94,343.92)	1.75(1.61,1.90)
Tokelau	both	0.59(0.13,1.35)	1.03(0.25,2.32)	0.74	42.58(9.59,97.15)	76.33(18.51,173.52)	2.12(2.05,2.18)
Tuvalu	both	3.50(0.73,8.80)	8.96(2.09,20.59)	1.56	49.07(10.37,121.91)	85.47(19.94,195.39)	1.91(1.82,2.01)
Afghanistan	female	241.57(42.85,628.97)	1054.17(216.78,2734.73)	3.36	6.77(1.20,17.51)	15.43(3.29,38.41)	3.14(2.89,3.38)
Albania	female	75.23(13.91,189.61)	254.22(45.46,656.46)	2.38	7.12(1.32,17.94)	11.10(1.98,28.88)	1.08(0.73,1.44)
Algeria	female	363.91(67.15,908.40)	1838.05(371.65,4221.46)	4.05	6.04(1.12,14.89)	11.37(2.32,26.00)	2.18(2.08,2.28)
American Samoa	female	4.57(0.94,10.56)	18.85(4.62,41.70)	3.13	41.46(8.55,95.31)	74.85(18.27,166.28)	2.14(2.03,2.25)

Andorra	female	3.02(0.57,8.17)	13.42(2.45,34.34)	3.45	11.00(2.06,29.61)	19.70(3.57,50.58)	2.01(1.79,2.23)
Angola	female	97.62(17.79,256.75)	561.38(105.25,1423.40)	4.75	5.05(0.90,13.29)	9.39(1.78,23.79)	2.19(2.05,2.33)
Antigua and Barbuda	female	2.30(0.46,5.36)	9.52(2.01,21.81)	3.14	7.84(1.56,18.41)	17.69(3.76,40.49)	2.73(2.64,2.83)
Argentina	female	2184.23(407.32,5200.26)	7664.16(1465.58,18025.25)	2.51	11.95(2.22,28.42)	25.87(4.97,61.13)	2.68(2.62,2.74)
Armenia	female	209.82(38.31,503.59)	456.81(92.70,1085.68)	1.18	13.21(2.41,31.46)	18.90(3.81,44.79)	1.25(0.94,1.56)
Australia	female	1386.55(247.19,3404.79)	4990.56(968.48,11990.69)	2.60	12.79(2.27,31.40)	22.58(4.35,54.17)	1.74(1.29,2.19)
Austria	female	708.07(122.61,1733.66)	2338.38(426.85,5515.83)	2.30	9.75(1.66,23.80)	25.90(4.74,60.66)	3.83(3.60,4.05)
Azerbaijan	female	238.69(44.62,580.09)	869.24(167.80,2126.90)	2.64	8.13(1.51,19.70)	16.34(3.15,39.61)	3.07(2.73,3.41)
Bahrain	female	31.98(6.52,72.83)	152.40(35.96,323.64)	3.77	47.33(9.78,106.81)	49.49(11.80,103.13)	-0.13(-0.69,0.43)
Bangladesh	female	553.25(90.78,1421.91)	3412.55(569.93,9010.77)	5.17	2.68(0.44,7.13)	5.44(0.91,14.43)	2.40(2.28,2.52)
Barbados	female	15.65(3.13,36.28)	43.38(9.31,102.83)	1.77	9.41(1.86,21.80)	15.83(3.39,37.36)	2.18(1.95,2.42)
Belarus	female	569.46(106.53,1383.76)	487.02(85.51,1247.36)	-0.14	6.88(1.30,16.80)	5.00(0.87,12.79)	-1.80(-2.07,-1.53)
Belgium	female	1161.04(211.41,2713.65)	3453.81(668.84,8123.49)	1.97	13.35(2.44,31.33)	30.62(5.91,72.83)	3.36(3.11,3.62)
Belize	female	3.53(0.68,8.33)	23.48(4.67,56.07)	5.65	7.79(1.49,18.38)	17.08(3.43,40.75)	2.97(2.54,3.41)
Benin	female	42.94(7.57,110.87)	215.83(40.49,522.09)	4.03	4.37(0.76,11.14)	8.98(1.72,21.43)	2.57(2.38,2.75)
Bermuda	female	7.58(1.37,18.65)	14.26(2.84,35.23)	0.88	21.39(3.87,52.53)	19.36(3.79,48.00)	-0.82(-1.01,-0.64)
Bhutan	female	3.49(0.57,10.08)	19.98(3.52,51.39)	4.72	2.84(0.45,8.36)	7.37(1.31,19.02)	3.35(3.30,3.40)
Bolivia (Plurinational State of)	female	166.15(30.55,430.86)	818.87(155.37,2147.29)	3.93	9.96(1.81,25.56)	17.92(3.43,47.16)	1.86(1.76,1.96)
Bosnia and Herzegovina	female	387.49(69.58,931.56)	1373.80(284.36,3395.41)	2.55	16.45(2.96,39.22)	40.49(8.30,100.61)	3.65(3.40,3.91)
Botswana	female	31.99(6.11,80.18)	197.55(35.88,500.70)	5.18	10.41(2.01,26.12)	26.47(4.77,66.11)	3.65(3.45,3.85)
Brazil	female	6990.79(1321.18,16493.31)	26799.60(5074.73,62727.62)	2.83	14.81(2.79,34.84)	20.50(3.89,47.96)	1.42(1.29,1.55)
Brunei Darussalam	female	40.70(8.56,91.22)	159.42(35.07,348.21)	2.92	92.70(19.65,206.63)	111.42(24.84,236.21)	0.64(0.47,0.81)
Bulgaria	female	717.41(140.00,1774.52)	1662.62(316.91,4064.93)	1.32	10.20(2.00,25.35)	22.03(4.21,54.45)	4.06(3.53,4.59)
Burkina Faso	female	88.10(15.00,229.45)	317.91(55.40,803.28)	2.61	4.04(0.70,10.46)	6.82(1.21,17.12)	1.91(1.67,2.16)
Burundi	female	37.14(6.47,96.34)	77.02(11.78,205.63)	1.07	2.92(0.52,7.57)	3.62(0.56,9.63)	0.55(0.44,0.65)

Cambodia	female	191.50(33.96,493.15)	1558.30(306.59,3785.66)	7.14	7.61(1.31,19.44)	22.75(4.53,54.62)	3.75(3.47,4.03)
Cameroon	female	112.99(21.34,303.42)	657.11(121.67,1748.45)	4.82	5.34(1.02,14.28)	11.64(2.18,30.51)	2.78(2.62,2.95)
Canada	female	4031.53(699.26,9964.95)	14138.51(2607.40,34024.38)	2.51	22.33(3.88,55.23)	38.59(7.03,92.88)	1.47(1.17,1.77)
Cabo Verde	female	11.06(1.95,27.27)	51.63(10.52,123.47)	3.67	8.21(1.46,20.37)	22.25(4.49,52.70)	3.75(3.56,3.95)
Central African Republic	female	31.60(5.74,80.44)	81.80(14.62,211.01)	1.59	4.99(0.95,12.71)	6.95(1.24,17.49)	1.29(1.19,1.38)
Chad	female	47.25(7.72,126.13)	180.30(31.70,470.28)	2.82	3.36(0.55,8.98)	7.65(1.38,19.86)	3.11(3.03,3.20)
Chile	female	729.96(141.18,1766.74)	3463.61(700.13,7757.48)	3.74	13.41(2.60,32.41)	25.87(5.24,57.97)	2.67(2.41,2.94)
China	female	106528.57(20273.06,269673.61)	318902.03(59577.67,789167.55)	1.99	24.32(4.63,61.22)	30.00(5.59,74.25)	0.80(0.42,1.18)
Colombia	female	1894.20(360.28,4401.76)	5752.68(1143.07,13645.76)	2.04	21.83(4.19,50.67)	20.04(3.98,47.54)	-0.78(-0.97,-0.59)
Comoros	female	3.26(0.57,8.56)	12.51(2.17,31.90)	2.83	2.89(0.51,7.61)	4.78(0.84,12.15)	1.61(1.49,1.74)
Congo	female	54.46(9.95,148.46)	225.38(38.94,601.43)	3.14	8.98(1.70,24.13)	16.78(3.00,43.82)	2.25(2.13,2.38)
Costa Rica	female	91.43(17.67,217.23)	344.14(69.68,838.86)	2.76	10.50(2.03,24.91)	12.53(2.54,30.56)	0.14(-0.06,0.35)
Côte d'Ivoire	female	81.49(14.80,207.10)	472.55(89.43,1180.29)	4.80	5.01(0.93,12.59)	10.41(1.95,25.64)	2.71(2.64,2.78)
Croatia	female	632.73(121.33,1497.90)	1366.79(260.96,3373.82)	1.16	16.32(3.14,38.98)	28.78(5.43,71.55)	2.71(2.40,3.02)
Cuba	female	1596.81(312.11,3757.77)	4674.40(890.48,11170.29)	1.93	30.61(5.99,72.22)	46.85(8.87,112.12)	1.89(1.68,2.10)
Cyprus	female	61.20(13.04,142.23)	222.37(43.01,498.36)	2.63	13.38(2.88,31.09)	21.30(4.14,48.02)	1.72(1.40,2.04)
Czechia	female	1626.46(311.86,3790.16)	5285.75(1137.45,12417.66)	2.25	20.05(3.80,47.15)	45.30(9.77,107.04)	3.02(2.82,3.23)
Democratic Republic of the Congo	female	428.51(72.95,1135.29)	1491.60(266.19,3837.40)	2.48	5.08(0.85,13.79)	7.84(1.40,20.42)	1.30(0.94,1.67)
Denmark	female	1030.68(185.99,2509.58)	2801.54(539.60,6719.68)	1.72	24.79(4.43,60.51)	46.23(8.88,112.29)	2.03(1.76,2.29)
Djibouti	female	2.20(0.38,5.61)	16.70(2.98,43.11)	6.60	3.44(0.62,8.75)	6.41(1.13,16.34)	2.24(2.16,2.32)
Dominica	female	6.36(1.27,15.08)	12.59(2.65,30.11)	0.98	15.25(3.03,36.47)	27.62(5.84,66.11)	2.40(2.21,2.60)
Dominican Republic	female	118.81(20.68,290.01)	719.70(136.68,1896.20)	5.06	6.43(1.11,15.89)	15.05(2.86,39.60)	3.60(3.28,3.93)
Ecuador	female	195.00(36.68,467.49)	1306.55(256.15,3004.01)	5.70	7.52(1.43,18.00)	16.84(3.32,38.56)	3.02(2.65,3.39)
Egypt	female	590.21(111.11,1465.55)	4055.52(756.60,10898.42)	5.87	4.12(0.77,10.14)	13.99(2.72,37.16)	4.37(4.26,4.48)

El Salvador	female	146.62(26.46,353.79)	645.71(136.02,1621.64)	3.40	9.46(1.72,22.81)	19.22(4.02,48.56)	2.29(2.13,2.46)
Equatorial Guinea	female	4.43(0.75,12.21)	48.33(8.29,132.01)	9.90	4.01(0.65,11.08)	18.18(3.19,49.11)	6.30(5.90,6.70)
Eritrea	female	14.52(2.34,41.97)	92.12(16.30,235.50)	5.34	2.56(0.42,7.40)	6.11(1.10,15.48)	2.93(2.68,3.18)
Estonia	female	136.66(25.21,333.13)	243.33(44.43,608.73)	0.78	10.37(1.92,25.35)	15.67(2.80,39.13)	1.81(1.58,2.05)
Ethiopia	female	156.32(25.26,426.77)	457.69(66.13,1325.51)	1.93	1.63(0.26,4.52)	2.38(0.35,6.85)	0.91(0.73,1.08)
Micronesia (Federated States of)	female	5.42(1.11,13.59)	22.48(4.42,55.83)	3.15	22.74(4.68,57.65)	59.56(12.00,148.20)	3.42(3.10,3.73)
Fiji	female	30.86(5.96,73.50)	122.41(26.20,281.38)	2.97	16.91(3.30,40.14)	30.15(6.60,69.27)	2.07(1.95,2.20)
Finland	female	658.43(119.66,1575.25)	1754.15(356.90,4069.90)	1.66	15.03(2.72,36.29)	26.58(5.40,62.76)	2.65(2.41,2.90)
France	female	2116.56(365.32,5305.52)	10495.40(1929.96,25929.11)	3.96	4.50(0.77,11.30)	15.75(2.88,39.23)	5.26(4.86,5.66)
Gabon	female	28.22(5.26,72.64)	94.31(16.56,248.82)	2.34	9.03(1.69,23.20)	17.56(3.09,45.71)	2.21(1.89,2.53)
Georgia	female	350.74(66.35,862.61)	547.26(109.18,1293.91)	0.56	9.19(1.74,22.56)	16.15(3.24,38.99)	2.97(2.45,3.50)
Germany	female	12643.96(2394.51,29381.29)	41090.26(8275.56,92989.76)	2.25	16.60(3.10,38.76)	43.67(8.75,100.03)	3.35(3.19,3.50)
Ghana	female	185.59(33.19,471.19)	677.67(130.76,1681.83)	2.65	5.92(1.09,14.92)	7.90(1.54,19.53)	0.59(0.30,0.89)
Greece	female	1060.05(184.34,2581.44)	2574.90(481.60,6053.42)	1.43	12.44(2.15,30.33)	22.04(4.05,53.06)	2.04(1.97,2.11)
Greenland	female	5.61(0.93,14.32)	24.86(4.69,64.07)	3.43	33.28(5.49,84.51)	76.56(14.66,197.59)	1.88(1.30,2.46)
Grenada	female	5.74(1.11,13.38)	14.79(3.04,33.24)	1.58	14.43(2.75,33.74)	25.16(5.14,56.75)	2.31(2.04,2.58)
Guam	female	12.04(2.56,28.80)	45.77(9.34,112.33)	2.80	33.46(7.13,80.21)	46.85(9.57,115.16)	1.18(0.85,1.52)
Guatemala	female	209.11(38.78,513.35)	937.25(195.19,2209.30)	3.48	12.03(2.27,28.95)	15.59(3.26,36.50)	0.03(-0.33,0.39)
Guinea	female	48.88(8.90,126.07)	169.18(29.27,426.41)	2.46	3.02(0.55,7.71)	6.63(1.14,16.72)	2.75(2.69,2.81)
Guinea-Bissau	female	9.66(1.79,24.70)	36.46(6.67,92.44)	2.77	4.83(0.90,12.26)	9.84(1.79,24.83)	2.66(2.58,2.75)
Guyana	female	22.88(4.49,53.19)	72.16(14.83,169.48)	2.15	11.68(2.31,27.15)	20.77(4.37,48.53)	2.02(1.83,2.20)
Haiti	female	197.12(39.35,485.24)	738.21(141.41,1868.92)	2.74	11.33(2.26,27.62)	19.51(3.73,48.26)	2.00(1.92,2.08)
Honduras	female	226.77(43.55,556.54)	2017.17(362.84,5512.63)	7.90	21.90(4.27,53.80)	63.11(11.44,171.25)	3.67(3.38,3.96)
Hungary	female	1969.32(384.93,4778.56)	6516.39(1234.31,15832.93)	2.31	22.96(4.48,56.01)	61.69(11.71,150.23)	3.78(3.22,4.34)

Iceland	female	34.03(6.11,83.60)	94.81(17.77,226.26)	1.79	22.64(4.09,55.46)	33.89(6.48,81.41)	1.11(0.83,1.38)
India	female	7698.06(1405.01,19118.83)	53818.45(10535.50,131262.28)	5.99	3.60(0.67,8.92)	9.15(1.80,22.25)	2.97(2.75,3.18)
Indonesia	female	4437.93(771.86,12285.77)	22656.75(3777.72,67524.35)	4.11	8.63(1.53,23.64)	20.33(3.36,60.20)	2.71(2.48,2.94)
Iran (Islamic Republic of)	female	672.44(127.17,1610.03)	6068.86(1284.29,14047.85)	8.03	5.63(1.05,13.41)	17.15(3.65,39.62)	4.05(3.90,4.21)
Iraq	female	409.14(73.55,1025.17)	3284.52(705.30,7990.18)	7.03	10.32(1.85,25.95)	28.15(6.10,67.33)	3.82(3.59,4.05)
Ireland	female	321.24(57.00,802.44)	1386.90(263.75,3276.63)	3.32	13.92(2.47,34.76)	35.10(6.70,82.58)	3.36(3.22,3.51)
Israel	female	439.36(80.35,1039.88)	1462.04(272.92,3389.61)	2.33	16.14(2.94,38.25)	23.20(4.38,54.36)	1.13(0.74,1.52)
Italy	female	7591.33(1456.46,17748.61)	17804.75(3455.66,40962.97)	1.35	14.73(2.82,34.58)	24.30(4.85,56.11)	2.10(1.70,2.51)
Jamaica	female	108.91(21.45,249.50)	347.73(71.91,836.22)	2.19	11.65(2.28,26.73)	23.03(4.75,55.45)	2.28(2.00,2.56)
Japan	female	10486.18(1910.51,25572.22)	20732.78(3807.43,51115.17)	0.98	10.66(1.95,26.03)	9.86(1.76,24.31)	-0.93(-1.17,-0.69)
Jordan	female	55.08(10.14,135.88)	480.44(99.28,1133.20)	7.72	8.88(1.63,21.25)	16.19(3.41,37.52)	2.02(1.67,2.36)
Kazakhstan	female	1458.42(272.92,3408.47)	1811.16(355.52,4133.96)	0.24	18.56(3.46,43.32)	17.45(3.42,39.98)	-0.27(-0.48,-0.07)
Kenya	female	48.14(7.11,142.74)	293.53(46.80,801.45)	5.10	1.20(0.18,3.53)	2.58(0.42,6.97)	3.06(2.81,3.30)
Kiribati	female	2.85(0.55,6.97)	8.46(1.70,20.15)	1.97	13.44(2.65,32.45)	21.16(4.29,50.09)	1.14(0.94,1.33)
Kuwait	female	31.77(6.34,79.98)	134.97(27.38,304.69)	3.25	15.08(2.99,37.04)	14.14(2.93,31.99)	-0.23(-0.64,0.18)
Kyrgyzstan	female	131.47(24.33,327.42)	167.30(29.81,411.89)	0.27	7.32(1.37,18.08)	6.44(1.15,15.81)	-0.74(-1.11,-0.36)
Lao People's Democratic Republic	female	148.46(27.32,379.44)	584.38(119.69,1457.08)	2.94	13.48(2.43,34.53)	26.61(5.55,65.40)	2.07(1.94,2.19)
Latvia	female	199.24(38.44,504.79)	287.17(54.14,702.64)	0.44	8.58(1.66,21.71)	12.24(2.28,30.24)	1.77(1.41,2.13)
Lebanon	female	157.41(29.85,386.39)	1122.44(219.03,2674.10)	6.13	13.56(2.54,33.12)	39.44(7.73,94.36)	4.25(4.01,4.50)
Lesotho	female	29.03(5.10,73.71)	119.41(21.53,312.33)	3.11	5.30(0.93,13.32)	16.06(2.94,41.61)	4.77(4.42,5.11)
Liberia	female	29.31(5.35,73.76)	88.24(16.11,216.87)	2.01	5.81(1.07,14.51)	9.74(1.79,23.81)	2.67(2.19,3.16)
Libya	female	45.75(8.57,119.73)	379.58(76.78,911.46)	7.30	5.36(1.00,13.61)	15.52(3.21,37.10)	3.94(3.64,4.24)
Lithuania	female	184.88(33.63,448.39)	267.07(49.08,662.86)	0.44	6.62(1.21,16.04)	7.95(1.47,19.61)	1.10(0.80,1.41)
Luxembourg	female	30.00(5.40,73.73)	186.25(38.23,429.92)	5.21	9.54(1.69,23.53)	36.68(7.53,85.16)	5.25(4.97,5.53)

North Macedonia	female	144.35(27.35,346.60)	628.80(124.79,1510.85)	3.36	14.22(2.71,34.25)	36.08(7.12,87.17)	3.68(3.34,4.01)
Madagascar	female	64.05(11.69,167.68)	233.26(43.05,618.58)	2.64	2.53(0.47,6.57)	4.16(0.78,10.90)	1.58(1.48,1.68)
Malawi	female	52.25(9.64,130.31)	156.46(28.00,395.44)	1.99	2.66(0.49,6.58)	4.05(0.72,10.14)	1.60(1.54,1.66)
Malaysia	female	595.24(113.53,1409.70)	3467.62(712.95,8482.17)	4.83	13.17(2.50,31.05)	26.54(5.43,64.08)	2.64(2.19,3.08)
Maldives	female	2.16(0.39,5.45)	9.98(1.93,24.01)	3.63	6.49(1.23,15.91)	8.16(1.58,19.48)	0.07(-0.21,0.35)
Mali	female	69.64(12.46,175.66)	279.11(51.29,692.06)	3.01	3.46(0.63,8.78)	7.11(1.31,17.66)	2.51(2.47,2.54)
Malta	female	23.68(4.64,56.09)	82.96(16.69,191.87)	2.50	9.82(1.93,23.23)	17.27(3.40,40.25)	2.05(1.97,2.13)
Marshall Islands	female	2.81(0.60,6.51)	13.76(2.91,31.76)	3.90	33.72(7.15,78.73)	77.36(16.63,177.98)	2.80(2.64,2.97)
Mauritania	female	28.16(4.76,78.63)	93.59(16.87,236.24)	2.32	5.46(0.93,15.18)	9.60(1.74,24.11)	1.81(1.66,1.96)
Mauritius	female	47.96(9.72,111.12)	202.16(43.33,462.38)	3.21	11.97(2.42,27.67)	20.93(4.47,47.92)	2.06(1.74,2.38)
Mexico	female	5118.79(1058.98,11208.65)	10662.25(2137.85,24389.34)	1.08	24.22(5.05,52.74)	17.15(3.45,39.08)	-1.62(-1.83,-1.41)
Republic of Moldova	female	310.47(58.46,754.01)	328.41(62.46,798.93)	0.06	11.44(2.14,27.67)	9.60(1.84,23.38)	0.18(-0.16,0.52)
Mongolia	female	52.53(9.58,135.73)	114.29(20.77,288.94)	1.18	9.39(1.73,24.29)	9.86(1.78,24.59)	-0.56(-0.87,-0.24)
Montenegro	female	96.40(17.48,236.89)	298.30(61.87,721.02)	2.09	27.36(4.99,66.96)	54.85(11.39,133.06)	2.74(2.47,3.01)
Morocco	female	224.92(40.75,568.38)	1605.21(310.25,3830.12)	6.14	3.30(0.60,8.39)	10.12(1.98,23.97)	4.13(4.04,4.22)
Mozambique	female	68.69(12.51,174.78)	313.45(55.37,840.33)	3.56	2.32(0.42,5.89)	5.41(0.97,14.46)	3.32(3.08,3.56)
Myanmar	female	2051.95(371.40,5278.15)	7544.37(1508.49,18390.94)	2.68	16.47(2.92,42.72)	29.28(5.92,70.85)	1.95(1.88,2.02)
Namibia	female	17.31(3.23,43.19)	62.62(11.86,157.25)	2.62	4.41(0.82,10.95)	7.96(1.52,19.81)	1.91(1.69,2.14)
Nepal	female	140.92(21.58,408.43)	959.13(165.73,2479.34)	5.81	3.16(0.47,9.49)	8.28(1.44,21.15)	3.25(3.10,3.41)
Netherlands	female	1499.18(273.19,3629.81)	6341.31(1209.35,15132.28)	3.23	13.86(2.51,33.54)	36.39(6.95,87.31)	3.53(3.20,3.85)
New Zealand	female	431.16(83.33,1050.64)	1204.97(222.84,2919.14)	1.79	20.83(4.03,50.69)	29.50(5.44,71.35)	0.52(0.10,0.94)
Nicaragua	female	52.75(9.22,130.78)	332.33(66.35,756.19)	5.30	6.78(1.19,16.95)	14.08(2.81,31.84)	2.34(2.05,2.62)
Niger	female	20.94(3.26,58.79)	156.66(24.08,447.37)	6.48	1.75(0.28,4.93)	4.26(0.67,11.65)	3.06(2.77,3.35)
Nigeria	female	466.58(76.52,1258.12)	2027.62(346.70,5321.76)	3.35	2.28(0.38,6.15)	4.98(0.89,12.99)	2.60(2.15,3.04)

Democratic People's Republic of Korea	female	1414.86(256.17,3627.95)	4311.93(822.91,10904.54)	2.05	14.24(2.58,35.82)	22.81(4.37,57.89)	1.67(1.62,1.71)
Northern Mariana Islands	female	2.95(0.59,7.31)	16.17(3.27,37.22)	4.48	45.15(9.06,111.82)	60.25(12.27,138.89)	0.97(0.91,1.03)
Norway	female	606.64(116.02,1440.27)	1838.91(365.35,4326.27)	2.03	16.78(3.23,40.18)	37.18(7.32,87.92)	2.51(2.26,2.76)
Oman	female	17.56(3.03,48.09)	103.10(20.38,240.10)	4.87	6.09(1.06,16.71)	15.57(3.20,36.25)	3.10(2.72,3.47)
Pakistan	female	938.17(162.98,2382.30)	6311.41(1249.85,15628.45)	5.73	3.67(0.64,9.30)	11.81(2.31,29.44)	4.44(4.15,4.72)
Palestine	female	41.53(7.05,111.61)	284.27(57.91,677.89)	5.84	8.81(1.50,23.64)	24.22(4.97,57.18)	3.85(3.61,4.09)
Panama	female	78.01(14.95,186.22)	357.60(72.03,863.62)	3.58	10.83(2.09,25.78)	16.96(3.42,40.87)	1.23(1.03,1.44)
Papua New Guinea	female	102.53(14.59,298.71)	529.22(90.01,1459.09)	4.16	11.10(1.56,32.85)	23.39(4.03,63.89)	2.64(2.61,2.67)
Paraguay	female	72.05(13.06,176.22)	370.86(72.73,925.81)	4.15	6.34(1.15,15.54)	13.03(2.56,32.44)	2.50(2.28,2.72)
Peru	female	528.16(92.92,1302.15)	2031.91(377.08,5164.58)	2.85	8.98(1.59,22.10)	12.44(2.31,31.38)	0.71(0.39,1.03)
Philippines	female	2313.44(420.38,5457.24)	7435.72(1434.36,18383.14)	2.21	16.17(2.93,38.12)	18.18(3.54,44.73)	-0.44(-0.77,-0.12)
Poland	female	5210.94(987.84,12241.68)	17530.89(3216.95,41716.13)	2.36	20.14(3.80,47.45)	44.45(8.26,105.92)	3.25(3.05,3.45)
Portugal	female	821.00(154.80,1915.73)	2393.96(491.52,5488.77)	1.92	10.08(1.90,23.55)	19.30(3.87,44.44)	2.49(2.23,2.76)
Puerto Rico	female	414.47(85.19,926.55)	854.60(182.20,1977.30)	1.06	20.95(4.30,47.03)	21.76(4.57,50.46)	-0.06(-0.26,0.16)
Qatar	female	5.61(1.08,13.69)	89.48(21.42,192.68)	14.95	17.98(3.36,44.71)	49.27(12.37,102.81)	3.99(3.31,4.67)
Romania	female	1225.66(211.44,2994.63)	3432.55(627.48,8455.85)	1.80	7.65(1.32,18.75)	17.28(3.20,42.14)	2.96(2.85,3.08)
Russian Federation	female	8155.48(1518.08,19945.75)	11994.35(2147.91,29437.15)	0.47	6.79(1.27,16.56)	8.18(1.46,20.17)	0.69(0.45,0.93)
Rwanda	female	58.26(9.83,167.12)	171.22(30.97,426.04)	1.94	3.64(0.62,10.28)	5.12(0.92,12.75)	1.03(0.85,1.21)
Saint Lucia	female	11.62(2.38,25.71)	30.22(6.93,67.87)	1.60	23.83(4.89,52.92)	26.66(6.10,59.82)	-0.11(-0.47,0.24)
Saint Vincent and the Grenadines	female	5.93(1.23,13.50)	15.68(3.40,36.28)	1.64	14.84(3.11,33.98)	23.57(5.11,54.32)	1.67(1.47,1.87)
Samoa	female	5.96(1.18,14.44)	18.45(3.92,43.64)	2.09	13.38(2.65,32.35)	24.90(5.30,58.34)	2.05(1.96,2.14)
Sao Tome and Principe	female	2.43(0.46,6.07)	9.20(1.47,26.39)	2.79	7.24(1.38,18.10)	18.05(2.90,52.26)	3.26(3.14,3.38)
Saudi Arabia	female	148.39(26.02,399.96)	1105.62(209.09,2660.06)	6.45	6.28(1.13,16.96)	14.99(2.99,35.65)	2.25(1.77,2.73)

Senegal	female	93.13(16.65,229.26)	451.74(85.16,1090.95)	3.85	6.20(1.12,15.25)	12.28(2.35,29.42)	2.95(2.65,3.25)
Serbia	female	1328.69(247.34,3177.21)	4583.01(922.23,11079.37)	2.45	20.91(3.96,50.18)	53.63(10.76,131.40)	3.88(3.63,4.12)
Seychelles	female	3.01(0.58,7.31)	14.18(3.10,32.12)	3.71	9.26(1.76,22.58)	25.27(5.56,57.25)	3.37(3.01,3.73)
Sierra Leone	female	18.72(3.26,47.34)	99.95(17.30,251.36)	4.34	2.11(0.37,5.33)	5.98(1.02,15.14)	4.23(3.99,4.48)
Singapore	female	445.99(82.93,1034.92)	814.68(149.68,1925.51)	0.83	38.68(7.20,89.88)	20.27(3.72,47.94)	-2.29(-2.54,-2.03)
Slovakia	female	360.57(68.46,880.99)	955.33(184.40,2403.99)	1.65	10.30(1.94,25.13)	18.06(3.44,45.93)	2.44(2.15,2.73)
Slovenia	female	229.24(43.04,579.59)	554.98(104.25,1374.67)	1.42	15.36(2.85,39.03)	24.92(4.73,62.26)	1.88(1.58,2.18)
Solomon Islands	female	8.50(1.65,22.85)	52.96(9.92,136.11)	5.23	12.66(2.41,32.68)	32.50(6.28,81.69)	3.24(3.03,3.45)
Somalia	female	24.12(3.85,64.32)	92.24(13.61,275.69)	2.83	1.86(0.30,4.93)	2.47(0.37,7.42)	1.27(1.17,1.37)
South Africa	female	1815.17(341.99,4352.44)	6177.09(1222.77,14368.78)	2.40	15.51(2.93,37.31)	24.20(4.79,56.01)	1.56(1.19,1.94)
Republic of Korea	female	2222.03(423.20,5380.84)	8783.46(1660.94,20579.31)	2.95	12.96(2.47,31.19)	17.37(3.30,40.56)	-0.10(-0.60,0.40)
South Sudan	female	34.40(5.63,93.62)	79.47(13.16,225.48)	1.31	3.39(0.56,9.18)	4.61(0.79,12.70)	1.09(0.98,1.20)
Spain	female	2759.71(524.32,6389.19)	9058.63(1697.92,21019.70)	2.28	8.59(1.63,19.97)	19.60(3.63,45.53)	3.83(3.45,4.21)
Sri Lanka	female	328.63(62.63,774.42)	2389.75(529.95,5494.89)	6.27	6.40(1.24,15.06)	16.46(3.69,37.82)	3.71(3.52,3.90)
Sudan	female	153.93(24.99,407.30)	921.43(178.26,2322.48)	4.99	3.51(0.57,9.45)	10.93(2.12,26.85)	4.07(3.98,4.16)
Suriname	female	21.00(4.12,50.81)	101.03(21.32,237.93)	3.81	15.72(3.08,37.88)	30.94(6.55,72.93)	2.71(2.45,2.96)
Eswatini	female	16.69(3.02,43.28)	68.93(12.08,177.16)	3.13	10.81(1.95,27.78)	20.48(3.57,52.34)	2.54(2.08,3.00)
Sweden	female	1040.52(188.30,2530.99)	3204.86(662.96,7537.41)	2.08	13.32(2.48,32.35)	29.62(6.10,69.85)	3.08(2.68,3.48)
Switzerland	female	989.22(191.70,2372.43)	2223.06(422.00,5233.25)	1.25	17.18(3.34,41.46)	25.48(4.83,60.52)	2.11(1.68,2.53)
Syrian Arab Republic	female	168.99(32.38,432.93)	866.56(168.07,2046.00)	4.13	6.78(1.29,17.18)	14.75(2.96,34.61)	2.28(1.95,2.62)
Taiwan (Province of China)	female	1711.21(322.47,4049.62)	7402.22(1442.35,17651.40)	3.33	22.90(4.35,54.01)	34.53(6.76,82.55)	1.08(0.84,1.32)
Tajikistan	female	124.70(22.21,304.51)	350.35(64.90,852.96)	1.81	8.06(1.44,19.72)	13.02(2.53,31.51)	2.76(2.13,3.39)
United Republic of Tanzania	female	126.48(21.68,321.60)	636.39(117.52,1604.59)	4.03	2.34(0.40,5.91)	5.19(0.96,12.98)	3.38(3.20,3.56)
Thailand	female	4144.61(832.90,10201.32)	12868.47(2504.54,31959.89)	2.10	22.22(4.39,54.38)	23.06(4.48,57.30)	-1.05(-1.40,-0.69)
Bahamas	female	11.01(2.09,26.02)	41.91(8.14,99.04)	2.81	12.94(2.46,30.60)	19.32(3.75,45.39)	1.75(1.62,1.87)

Gambia	female	4.25(0.75,10.84)	35.48(6.36,93.48)	7.35	2.76(0.49,7.05)	7.62(1.37,20.16)	3.58(3.39,3.77)
Timor-Leste	female	9.29(1.60,24.72)	83.76(15.80,211.88)	8.02	7.19(1.23,19.01)	20.18(3.81,50.20)	3.75(3.43,4.07)
Togo	female	22.21(4.02,57.41)	127.82(22.86,316.69)	4.75	3.73(0.67,9.54)	6.65(1.19,16.44)	1.90(1.73,2.08)
Tonga	female	6.62(1.29,15.61)	15.80(3.38,37.89)	1.39	22.97(4.45,53.99)	38.20(8.12,91.30)	1.68(1.55,1.81)
Trinidad and Tobago	female	62.69(12.93,137.46)	154.13(32.47,375.86)	1.46	14.30(2.96,31.20)	15.72(3.31,38.34)	0.04(-0.13,0.22)
Tunisia	female	89.23(16.58,218.59)	631.02(129.94,1542.49)	6.07	3.68(0.69,9.02)	9.72(2.01,23.76)	3.33(3.19,3.47)
Turkey	female	2214.25(379.16,5493.25)	9140.73(1726.30,21979.09)	3.13	11.85(2.02,29.06)	19.57(3.69,46.99)	3.46(2.42,4.50)
Turkmenistan	female	69.69(12.23,170.28)	166.06(31.46,405.70)	1.38	6.12(1.08,14.86)	7.34(1.41,17.89)	0.58(0.10,1.07)
Uganda	female	81.29(14.39,206.42)	520.72(97.97,1298.41)	5.41	2.55(0.45,6.36)	6.91(1.32,17.09)	3.66(3.42,3.90)
Ukraine	female	4685.82(824.72,11479.68)	3025.04(533.27,7744.55)	-0.35	10.10(1.78,24.91)	6.67(1.18,17.19)	-2.24(-2.60,-1.88)
United Arab Emirates	female	30.14(4.84,81.40)	367.71(72.39,860.90)	11.20	27.23(4.35,76.75)	46.40(8.55,115.01)	2.07(1.04,3.11)
United Kingdom	female	18705.78(3549.54,44304.28)	40221.41(8203.16,91258.72)	1.15	36.40(6.86,86.17)	60.21(12.17,137.55)	1.83(1.70,1.95)
United States of America	female	97268.80(18402.20,227181.67)	204567.00(42460.94,462476.34)	1.10	56.58(10.66,131.92)	67.46(13.93,153.07)	1.40(1.04,1.75)
Uruguay	female	113.77(20.25,279.40)	545.78(100.76,1329.32)	3.80	5.13(0.91,12.59)	18.89(3.48,45.90)	4.99(4.57,5.41)
Uzbekistan	female	406.50(76.70,989.14)	1403.02(262.11,3379.70)	2.45	6.19(1.16,15.10)	11.51(2.20,27.52)	2.09(1.94,2.24)
Vanuatu	female	3.74(0.65,10.02)	25.73(5.04,62.86)	5.89	12.87(2.25,33.75)	30.96(6.08,75.49)	2.88(2.64,3.11)
Venezuela (Bolivarian Republic of)	female	1139.99(218.97,2675.75)	4954.25(991.07,12660.60)	3.35	22.95(4.38,53.61)	31.78(6.32,80.74)	1.39(1.23,1.55)
Viet nam	female	1960.84(390.74,5062.68)	11715.51(2207.39,29013.88)	4.97	8.44(1.69,21.74)	22.80(4.34,55.99)	3.65(3.56,3.74)
Virginia	female	2349.37(446.10,5562.27)	5367.31(1083.24,12893.29)	1.28	60.03(11.34,142.56)	68.54(13.89,165.54)	1.19(0.80,1.58)
Yemen	female	68.47(10.79,206.06)	492.88(92.12,1223.83)	6.20	2.76(0.42,8.37)	7.39(1.37,18.12)	4.01(3.76,4.26)
Zambia	female	63.39(11.50,167.25)	250.73(45.61,652.50)	2.96	4.80(0.90,12.71)	7.45(1.36,19.12)	1.44(1.24,1.65)
Zimbabwe	female	212.90(39.26,517.49)	1013.75(193.52,2482.44)	3.76	10.66(1.97,25.83)	25.29(4.82,61.73)	3.95(3.48,4.42)
Monaco	female	5.89(1.07,15.09)	29.73(5.80,72.29)	4.05	15.56(2.81,40.52)	62.11(11.96,153.49)	5.44(4.70,6.20)
San Marino	female	1.99(0.36,4.90)	8.93(1.61,26.30)	3.49	10.79(1.98,26.74)	28.44(5.07,84.03)	4.28(3.91,4.65)

Saint Kitts and Nevis	female	4.05(0.80,9.42)	6.80(1.44,16.42)	0.68	18.91(3.76,43.74)	19.67(4.26,47.16)	0.63(0.43,0.82)
Cook Islands	female	1.78(0.36,4.25)	4.74(1.08,10.98)	1.67	30.30(6.13,72.29)	36.95(8.46,86.09)	0.54(0.44,0.63)
Nauru	female	0.64(0.11,1.62)	1.55(0.27,4.19)	1.44	36.01(6.64,89.36)	65.88(11.53,170.81)	1.97(1.69,2.26)
Niue	female	0.49(0.10,1.14)	0.92(0.21,2.13)	0.89	39.82(8.26,94.60)	77.89(17.45,181.51)	2.34(2.22,2.45)
Palau	female	4.02(0.80,9.86)	16.15(3.41,36.86)	3.02	80.99(16.16,199.75)	145.91(31.15,329.24)	2.07(1.93,2.21)
Tokelau	female	0.22(0.04,0.55)	0.41(0.09,1.04)	0.81	29.91(5.84,73.76)	59.72(12.58,151.65)	2.40(2.34,2.46)
Tuvalu	female	1.00(0.21,2.40)	2.93(0.60,6.89)	1.94	24.84(5.20,60.18)	52.19(10.72,122.63)	2.53(2.39,2.67)
Afghanistan	male	1345.51(160.39,4358.49)	2922.04(431.02,8455.94)	1.17	34.82(4.25,112.74)	53.45(8.22,149.71)	1.68(1.60,1.76)
Albania	male	465.64(71.58,1171.21)	1245.62(195.44,3300.48)	1.68	47.68(7.30,120.10)	58.36(9.13,154.60)	0.48(0.21,0.75)
Algeria	male	1852.82(309.11,4654.79)	6815.86(1265.39,16252.03)	2.68	31.11(5.24,77.37)	40.38(7.59,95.74)	1.08(0.73,1.43)
American Samoa	male	17.36(3.05,38.64)	43.40(8.56,93.05)	1.50	156.08(28.21,341.99)	184.36(36.19,391.67)	0.60(0.47,0.74)
Andorra	male	23.04(3.49,59.28)	66.69(10.61,159.26)	1.89	78.70(11.96,201.12)	94.56(15.04,224.94)	0.61(0.58,0.65)
Angola	male	830.76(122.51,2281.98)	2658.98(414.92,6466.44)	2.20	42.96(6.49,115.17)	53.60(8.31,128.06)	0.85(0.67,1.03)
Antigua and Barbuda	male	8.13(1.35,18.96)	16.90(3.07,38.36)	1.08	35.57(5.87,82.71)	35.28(6.42,80.01)	0.06(-0.29,0.42)
Argentina	male	10478.06(1690.20,25117.90)	19382.18(3233.38,44971.07)	0.85	70.45(11.36,168.23)	80.70(13.49,187.63)	0.02(-0.15,0.20)
Armenia	male	1067.96(169.68,2610.92)	2183.85(349.96,5231.14)	1.04	81.05(12.81,198.35)	116.53(18.49,278.37)	1.10(0.79,1.42)
Australia	male	4801.86(767.85,11532.78)	9445.92(1611.38,22244.02)	0.97	54.16(8.67,129.98)	47.16(7.99,111.84)	-0.71(-0.86,-0.56)
Austria	male	2418.54(375.47,5869.24)	4669.18(763.26,10898.20)	0.93	52.53(8.12,127.54)	60.06(9.79,140.44)	0.63(0.41,0.85)
Azerbaijan	male	1234.63(197.41,3110.71)	3768.08(614.74,9226.39)	2.05	52.62(8.37,132.21)	78.32(12.97,191.67)	1.81(1.37,2.26)
Bahrain	male	158.94(28.84,360.77)	512.76(98.35,1119.99)	2.23	211.11(39.11,463.87)	118.67(23.46,252.09)	-2.63(-3.11,-2.14)
Bangladesh	male	4526.07(687.14,11826.49)	12482.60(1888.45,36608.66)	1.76	17.90(2.71,46.87)	18.26(2.79,53.33)	0.34(0.11,0.57)
Barbados	male	37.16(6.11,86.70)	75.78(13.00,182.09)	1.04	30.32(4.99,70.93)	33.33(5.73,79.71)	-0.31(-0.56,-0.06)
Belarus	male	3497.86(556.03,8699.90)	3480.35(563.03,8787.40)	-0.01	67.46(10.62,167.61)	54.59(8.88,137.70)	-1.65(-2.05,-1.25)
Belgium	male	7769.63(1257.02,18883.16)	9605.83(1568.61,22349.60)	0.24	117.24(19.01,285.26)	94.65(15.45,220.53)	-0.87(-1.05,-0.69)
Belize	male	8.52(1.34,20.88)	52.76(8.72,127.51)	5.19	18.74(2.95,46.06)	37.61(6.16,90.05)	1.88(1.25,2.51)

Benin	male	170.03(25.88,425.79)	595.34(95.04,1449.10)	2.50	18.24(2.77,45.51)	29.37(4.72,70.56)	1.89(1.76,2.01)
Bermuda	male	26.09(4.29,61.80)	41.82(6.99,99.47)	0.60	97.69(16.16,231.10)	71.53(11.93,170.13)	-0.93(-1.10,-0.75)
Bhutan	male	13.16(1.93,36.64)	58.40(9.06,153.78)	3.44	11.09(1.62,30.12)	20.63(3.23,54.53)	2.42(2.28,2.55)
Bolivia (Plurinational State of)	male	368.12(54.08,1010.97)	1408.42(220.92,3687.77)	2.83	25.91(3.81,71.34)	34.71(5.47,90.55)	0.89(0.81,0.98)
Bosnia and Herzegovina	male	2004.77(319.64,4873.53)	5443.14(927.03,13143.77)	1.72	107.32(17.17,258.24)	193.75(32.96,467.26)	2.25(1.95,2.55)
Botswana	male	113.65(18.21,292.42)	438.70(77.58,1078.86)	2.86	45.27(7.51,115.12)	78.14(13.98,189.47)	1.66(1.18,2.15)
Brazil	male	20006.51(3161.89,47323.80)	42269.08(6887.62,97400.98)	1.11	48.16(7.63,113.04)	39.77(6.49,91.64)	-0.50(-0.62,-0.38)
Brunei Darussalam	male	105.45(21.16,234.54)	262.33(51.44,563.99)	1.49	267.70(53.84,601.54)	229.10(46.52,478.27)	0.12(-0.20,0.44)
Bulgaria	male	4355.97(704.09,10303.29)	7856.83(1277.31,19492.49)	0.80	70.72(11.39,168.30)	127.98(20.77,320.10)	3.52(2.92,4.12)
Burkina Faso	male	302.17(45.69,797.25)	1031.17(166.49,2646.86)	2.41	15.11(2.27,38.74)	27.46(4.48,69.31)	2.25(2.09,2.41)
Burundi	male	169.34(25.43,436.73)	341.03(53.42,873.63)	1.01	16.40(2.47,42.08)	15.70(2.48,40.17)	-0.38(-0.48,-0.27)
Cambodia	male	800.81(119.13,2098.31)	4639.63(806.12,11336.30)	4.79	43.68(6.43,114.60)	100.60(17.60,242.73)	2.93(2.77,3.10)
Cameroon	male	352.02(51.68,918.01)	1791.04(273.70,4591.33)	4.09	17.49(2.61,44.33)	34.80(5.39,88.76)	2.61(2.12,3.11)
Canada	male	9971.12(1580.36,24422.36)	20862.31(3421.54,48924.06)	1.09	67.79(10.73,165.97)	63.36(10.39,148.21)	-0.60(-0.72,-0.49)
Cabo Verde	male	16.28(2.55,40.50)	84.31(13.66,213.93)	4.18	16.17(2.51,40.22)	52.48(8.47,135.12)	3.01(2.40,3.63)
Central African Republic	male	268.43(31.90,898.46)	572.82(70.60,1908.86)	1.13	47.42(5.87,155.55)	55.91(7.59,179.64)	0.61(0.45,0.77)
Chad	male	195.55(28.47,522.38)	793.52(118.44,2142.69)	3.06	14.33(2.08,38.29)	28.25(4.29,75.64)	2.60(2.27,2.92)
Chile	male	1940.53(319.14,4622.63)	5328.98(932.99,12074.37)	1.75	42.87(7.03,101.63)	48.50(8.52,109.63)	0.59(0.48,0.71)
China	male	239040.34(37068.00,598716.77)	788751.67(130263.23,1958501.20)	2.30	57.49(8.96,142.24)	80.41(13.29,198.78)	1.48(1.12,1.84)
Colombia	male	3652.35(634.25,8481.62)	8178.49(1389.07,19932.67)	1.24	44.83(7.87,103.58)	34.50(5.87,83.93)	-1.31(-1.54,-1.08)
Comoros	male	12.55(1.98,33.09)	30.45(4.50,80.10)	1.43	12.01(1.90,31.64)	14.52(2.15,37.87)	0.52(0.40,0.65)
Congo	male	285.35(39.09,809.28)	651.73(107.04,1639.23)	1.28	59.68(8.36,165.58)	53.65(8.93,131.45)	-0.65(-0.86,-0.44)
Costa Rica	male	284.74(48.17,658.36)	831.75(145.53,2007.49)	1.92	34.97(5.94,80.58)	35.43(6.17,85.05)	-0.20(-0.43,0.03)
Côte d'Ivoire	male	387.65(58.37,1039.16)	1546.55(243.47,3895.24)	2.99	20.21(3.04,53.99)	32.14(5.16,80.35)	1.51(1.30,1.73)

Croatia	male	3853.76(607.50,9057.77)	5264.95(857.02,12665.04)	0.37	138.75(22.13,324.62)	137.90(22.43,333.98)	0.17(-0.11,0.46)
Cuba	male	5023.19(857.32,11501.63)	10840.90(1908.66,25056.84)	1.16	99.01(16.91,226.87)	121.31(21.31,278.61)	0.79(0.60,0.99)
Cyprus	male	328.56(54.51,758.45)	948.29(166.81,2094.33)	1.89	82.08(13.72,188.43)	97.42(17.16,215.19)	0.72(0.50,0.95)
Czechia	male	11657.00(2035.94,27044.85)	14878.84(2702.70,33839.45)	0.28	199.07(34.62,461.77)	155.23(28.03,354.24)	-0.81(-1.04,-0.57)
Democratic Republic of the Congo	male	3082.23(358.46,12811.48)	7240.76(896.80,27010.53)	1.35	41.86(5.08,172.25)	46.23(5.78,170.06)	0.17(0.02,0.33)
Denmark	male	2229.53(354.08,5492.86)	3436.26(550.78,8164.57)	0.54	63.13(10.00,155.31)	62.08(9.95,147.27)	-0.05(-0.23,0.14)
Djibouti	male	9.22(1.27,25.68)	70.12(10.00,215.16)	6.60	15.10(2.19,41.55)	24.08(3.54,71.57)	1.62(1.51,1.72)
Dominica	male	18.18(3.07,42.68)	38.50(6.98,87.42)	1.12	61.14(10.06,143.97)	85.68(15.48,193.51)	1.15(1.07,1.23)
Dominican Republic	male	241.48(36.91,597.22)	1398.41(220.51,3646.80)	4.79	13.47(2.05,33.13)	31.63(5.02,81.93)	3.58(3.36,3.80)
Ecuador	male	352.25(56.34,847.79)	1660.10(289.05,4025.86)	3.71	14.09(2.27,33.80)	23.72(4.16,57.17)	1.98(1.67,2.29)
Egypt	male	1204.12(184.87,3044.05)	8883.57(1518.50,23106.65)	6.38	7.92(1.22,20.04)	24.24(4.15,62.31)	4.24(4.09,4.38)
El Salvador	male	225.55(37.37,546.46)	815.31(137.81,1971.10)	2.61	16.76(2.78,40.51)	32.97(5.56,80.00)	2.33(1.94,2.72)
Equatorial Guinea	male	40.27(5.03,124.24)	111.43(17.43,300.78)	1.77	44.66(5.81,135.67)	59.35(9.48,159.46)	1.20(1.04,1.36)
Eritrea	male	53.51(8.21,150.42)	220.69(35.68,558.73)	3.12	13.84(2.08,38.15)	21.40(3.50,53.07)	1.29(1.01,1.57)
Estonia	male	720.50(111.00,1785.02)	749.44(118.17,1825.96)	0.04	91.46(14.10,224.78)	75.31(11.84,183.37)	-0.71(-1.06,-0.34)
Ethiopia	male	1331.87(188.18,4204.85)	1953.16(304.83,5144.99)	0.47	13.40(1.87,41.35)	10.10(1.58,26.38)	-1.43(-1.60,-1.26)
Micronesia (Federated States of)	male	18.82(3.08,50.20)	58.30(9.29,159.68)	2.10	81.42(13.59,210.92)	167.92(27.14,448.91)	2.55(2.20,2.90)
Fiji	male	76.50(13.14,177.09)	227.05(44.75,503.95)	1.97	46.59(8.22,106.90)	66.20(13.47,144.20)	0.89(0.73,1.06)
Finland	male	2625.09(436.94,6176.94)	3566.20(605.60,8276.29)	0.36	91.86(15.26,215.85)	63.20(10.72,146.60)	-1.16(-1.31,-1.00)
France	male	18090.05(2838.31,44647.44)	34306.22(5372.07,83193.18)	0.90	52.13(8.16,128.44)	60.08(9.37,146.53)	0.44(0.31,0.57)
Gabon	male	172.36(23.96,518.22)	405.59(63.81,1055.11)	1.35	67.71(9.53,200.26)	81.75(13.53,209.70)	0.57(0.46,0.68)
Georgia	male	1887.88(296.61,4646.95)	3926.13(663.13,9220.48)	1.08	69.29(10.84,170.64)	157.98(26.61,371.99)	4.28(3.55,5.02)
Germany	male	52437.24(8659.91,124923.49)	86452.97(15227.60,197950.42)	0.65	105.89(17.53,251.77)	103.30(18.32,237.14)	-0.35(-0.47,-0.23)

Ghana	male	333.30(50.69,846.28)	1773.61(297.00,4291.86)	4.32	12.15(1.90,30.47)	27.23(4.64,65.35)	3.47(3.02,3.91)
Greece	male	6369.40(1036.47,15585.70)	12561.96(2132.02,29311.03)	0.97	88.72(14.44,217.08)	125.58(21.26,293.94)	1.15(1.04,1.26)
Greenland	male	19.18(3.00,48.02)	65.48(10.76,160.18)	2.41	117.06(18.96,287.99)	170.31(28.24,411.33)	1.21(1.15,1.28)
Grenada	male	16.24(2.78,37.99)	36.51(6.64,82.33)	1.25	54.30(9.19,126.99)	66.24(12.05,147.36)	0.99(0.51,1.48)
Guam	male	33.95(5.34,83.41)	95.63(16.41,223.90)	1.82	93.97(15.02,230.37)	102.30(17.49,238.34)	0.37(0.02,0.72)
Guatemala	male	288.88(44.65,698.50)	1406.83(250.03,3360.23)	3.87	16.68(2.63,40.41)	28.87(5.13,68.72)	1.89(1.38,2.39)
Guinea	male	199.21(29.09,503.01)	612.08(100.50,1494.03)	2.07	12.18(1.78,30.54)	22.70(3.75,55.34)	2.24(2.03,2.46)
Guinea-Bissau	male	51.46(6.76,148.15)	106.60(16.54,295.60)	1.07	26.65(3.57,75.67)	36.12(5.65,98.59)	1.30(1.18,1.41)
Guyana	male	52.45(8.72,123.27)	121.39(21.28,287.00)	1.31	28.91(4.84,67.18)	40.14(7.19,93.60)	1.30(1.15,1.45)
Haiti	male	674.90(95.88,2099.96)	1471.17(221.41,4326.58)	1.18	42.71(6.24,131.67)	45.69(7.00,132.04)	0.35(0.13,0.56)
Honduras	male	319.49(52.81,797.02)	1695.97(276.38,4414.89)	4.31	32.23(5.33,80.58)	59.97(9.80,155.60)	2.42(2.22,2.62)
Hungary	male	8859.51(1438.89,21230.09)	14375.04(2444.78,33880.88)	0.62	140.69(22.96,338.07)	180.69(30.83,428.46)	0.71(0.16,1.27)
Iceland	male	51.70(8.32,125.17)	138.74(23.10,326.83)	1.68	39.09(6.29,94.86)	52.43(8.71,124.15)	1.06(0.87,1.25)
India	male	37045.26(5899.89,92520.80)	149996.91(26583.41,362405.35)	3.05	16.04(2.56,39.80)	26.62(4.75,63.96)	1.43(1.33,1.54)
Indonesia	male	12570.68(1937.08,30604.17)	55664.80(9217.54,141609.02)	3.43	28.16(4.36,68.30)	55.99(9.24,141.31)	2.34(2.23,2.45)
Iran (Islamic Republic of)	male	3017.03(490.61,7665.59)	12994.23(2246.65,29904.48)	3.31	22.42(3.61,57.23)	36.87(6.39,84.69)	2.16(1.90,2.42)
Iraq	male	2086.90(329.95,5243.28)	8970.41(1549.06,21316.07)	3.30	56.39(8.89,140.88)	83.38(14.66,194.76)	1.47(1.19,1.74)
Ireland	male	815.22(127.15,1998.82)	2258.00(374.86,5298.87)	1.77	42.41(6.60,104.08)	62.36(10.33,146.67)	1.42(1.25,1.58)
Israel	male	991.65(161.52,2383.22)	3087.20(534.35,7148.68)	2.11	44.54(7.24,107.05)	58.33(9.98,135.43)	0.62(0.18,1.06)
Italy	male	45453.25(7384.27,107707.48)	57519.31(10025.38,129204.32)	0.27	115.13(18.72,273.17)	92.29(16.01,207.89)	-0.67(-1.22,-0.11)
Jamaica	male	352.78(58.29,839.70)	1262.49(218.82,2995.67)	2.58	43.12(7.09,103.06)	88.18(15.38,208.88)	2.47(1.75,3.20)
Japan	male	43098.06(6983.04,103015.33)	75755.68(12464.10,179087.86)	0.76	58.65(9.52,139.53)	46.83(7.67,111.42)	-1.04(-1.18,-0.90)
Jordan	male	299.53(48.32,739.56)	2112.97(387.03,4910.26)	6.05	44.58(7.33,109.80)	63.21(11.97,146.67)	1.57(1.27,1.88)
Kazakhstan	male	5259.05(839.26,12912.09)	6155.63(994.98,14956.18)	0.17	98.50(15.81,241.13)	82.09(13.46,197.89)	-0.51(-0.67,-0.35)
Kenya	male	293.02(46.71,737.76)	1350.95(215.78,3332.65)	3.61	7.93(1.27,19.71)	14.41(2.35,35.29)	2.09(1.74,2.45)

Kiribati	male	15.51(2.51,38.95)	48.56(9.03,113.50)	2.13	88.78(14.14,218.13)	151.27(28.90,347.82)	1.94(1.73,2.14)
Kuwait	male	140.34(24.58,334.34)	619.83(111.60,1397.10)	3.42	40.99(7.31,96.60)	47.84(8.78,105.69)	0.62(0.11,1.13)
Kyrgyzstan	male	573.86(87.65,1415.90)	540.87(79.26,1346.77)	-0.06	44.22(6.78,109.29)	26.38(3.90,65.25)	-1.92(-2.42,-1.42)
Lao People's Democratic Republic	male	612.41(96.53,1652.96)	1599.20(269.81,4011.51)	1.61	62.51(9.92,168.67)	81.19(13.72,201.19)	0.61(0.50,0.71)
Latvia	male	1007.38(155.51,2530.80)	1065.99(166.25,2635.23)	0.06	73.03(11.30,183.13)	73.06(11.43,180.64)	0.06(-0.38,0.49)
Lebanon	male	735.60(109.91,1968.46)	2767.45(503.44,6479.20)	2.76	63.35(9.52,166.85)	118.14(21.49,275.44)	3.00(2.66,3.35)
Lesotho	male	125.80(19.42,341.00)	343.73(56.49,885.80)	1.73	29.87(4.77,80.24)	65.92(10.86,170.59)	3.30(3.04,3.56)
Liberia	male	118.31(18.54,295.17)	240.74(38.74,597.56)	1.03	19.66(3.07,48.82)	26.72(4.41,65.87)	1.73(1.39,2.08)
Libya	male	656.88(103.83,1656.46)	2666.58(468.40,6290.64)	3.06	68.72(11.02,172.83)	106.86(18.88,248.18)	1.63(1.42,1.85)
Lithuania	male	1247.60(190.25,3055.09)	1304.56(201.47,3255.27)	0.05	68.89(10.48,168.35)	61.29(9.48,152.57)	-0.38(-0.71,-0.04)
Luxembourg	male	163.10(25.95,404.63)	518.65(88.99,1188.86)	2.18	71.05(11.36,176.23)	111.93(19.23,255.97)	1.73(1.56,1.91)
North Macedonia	male	823.70(136.54,1989.38)	3139.19(554.89,7609.36)	2.81	86.95(14.38,209.70)	189.33(33.61,457.08)	2.79(2.35,3.23)
Madagascar	male	253.92(41.31,648.06)	599.80(89.66,1609.74)	1.36	10.17(1.67,26.02)	12.57(1.87,33.21)	0.65(0.52,0.78)
Malawi	male	191.24(31.00,477.77)	539.00(86.13,1298.25)	1.82	11.58(1.88,28.48)	18.32(2.86,43.80)	1.72(1.29,2.15)
Malaysia	male	1896.27(297.90,4577.30)	8325.83(1393.74,20774.78)	3.39	45.66(7.15,109.93)	64.17(10.80,158.85)	0.96(0.57,1.36)
Maldives	male	11.97(1.87,32.95)	36.12(5.99,85.06)	2.02	26.15(4.23,69.85)	26.43(4.41,61.54)	-0.54(-0.77,-0.31)
Mali	male	169.20(24.83,429.07)	570.94(94.55,1444.21)	2.37	8.57(1.27,21.83)	13.80(2.30,34.62)	1.77(1.66,1.87)
Malta	male	186.16(30.55,443.23)	351.15(58.65,794.95)	0.89	95.99(15.76,228.19)	78.98(13.29,180.11)	-0.53(-0.67,-0.39)
Marshall Islands	male	12.06(1.84,34.04)	38.19(6.42,104.18)	2.17	151.92(23.61,428.04)	207.94(34.71,556.20)	1.32(1.17,1.48)
Mauritania	male	55.29(8.28,141.32)	140.73(21.31,378.84)	1.55	12.00(1.84,30.59)	14.29(2.19,38.06)	0.51(0.28,0.74)
Mauritius	male	171.24(28.53,403.94)	538.28(95.29,1196.13)	2.14	52.28(8.78,122.96)	66.64(11.82,146.65)	1.14(0.86,1.42)
Mexico	male	10877.17(1933.43,24019.32)	21611.58(3932.08,48505.16)	0.99	55.64(9.96,122.56)	40.44(7.35,90.73)	-1.60(-1.92,-1.29)
Republic of Moldova	male	1409.17(221.75,3455.74)	1418.14(223.86,3414.11)	0.01	70.79(11.18,172.46)	56.33(8.96,135.56)	-0.02(-0.46,0.41)
Mongolia	male	186.42(28.66,490.20)	481.25(78.05,1246.67)	1.58	40.55(6.24,106.49)	51.42(8.32,130.70)	0.58(0.48,0.69)

Montenegro	male	398.99(65.55,939.14)	1064.47(177.59,2517.45)	1.67	142.07(23.65,334.26)	228.24(38.05,540.52)	1.65(1.42,1.89)
Morocco	male	2628.70(402.16,6629.96)	12370.06(2156.57,30656.41)	3.71	37.64(5.80,93.79)	75.88(13.13,186.73)	2.32(2.19,2.45)
Mozambique	male	253.02(38.17,619.17)	1048.16(160.53,2580.72)	3.14	10.09(1.54,24.81)	24.12(3.85,58.09)	3.67(3.37,3.97)
Myanmar	male	5928.85(882.00,17112.62)	14211.88(2332.98,37238.84)	1.40	55.56(8.31,159.99)	73.78(12.16,193.83)	0.82(0.70,0.94)
Namibia	male	50.23(7.90,122.80)	132.26(21.56,320.18)	1.63	15.41(2.43,37.30)	23.81(3.87,57.22)	1.74(1.60,1.88)
Nepal	male	649.29(96.61,1747.91)	2567.25(431.20,6318.62)	2.95	13.45(2.00,36.36)	23.91(4.04,58.36)	1.79(1.56,2.02)
Netherlands	male	9945.67(1593.29,23865.95)	11982.75(1958.38,28631.57)	0.20	115.36(18.47,276.46)	73.18(11.92,174.88)	-1.83(-2.02,-1.64)
New Zealand	male	860.35(135.87,2123.03)	1508.44(245.33,3564.43)	0.75	48.31(7.61,118.94)	40.71(6.61,96.25)	-1.45(-1.93,-0.97)
Nicaragua	male	171.66(28.42,397.60)	596.04(106.87,1383.79)	2.47	25.98(4.40,60.55)	31.73(5.77,73.28)	0.55(0.44,0.66)
Niger	male	97.46(14.15,269.54)	548.25(76.22,1532.16)	4.63	7.46(1.05,20.93)	16.24(2.36,45.17)	3.02(2.69,3.36)
Nigeria	male	2011.87(287.18,5478.15)	4989.92(783.45,12987.79)	1.48	10.16(1.45,27.12)	14.10(2.25,36.28)	1.27(1.15,1.39)
Democratic People's Republic of Korea	male	3110.85(450.74,8202.62)	9368.75(1470.96,23900.90)	2.01	48.80(7.10,126.91)	69.00(10.95,173.96)	1.27(1.20,1.33)
Northern Mariana Islands	male	14.77(2.35,35.90)	54.51(10.03,123.00)	2.69	158.45(26.27,379.19)	201.99(37.37,447.82)	0.77(0.63,0.91)
Norway	male	1845.30(301.85,4386.06)	2490.69(409.18,5743.89)	0.35	61.99(10.06,148.17)	54.40(8.93,125.80)	-0.69(-0.98,-0.40)
Oman	male	86.21(13.24,223.49)	296.81(50.84,727.90)	2.44	26.49(4.23,66.94)	38.19(6.88,88.00)	1.63(1.39,1.87)
Pakistan	male	10664.49(1652.03,26190.01)	35115.85(5880.88,91967.74)	2.29	33.88(5.28,83.03)	59.79(10.10,155.24)	2.05(1.67,2.43)
Palestine	male	285.29(45.05,709.78)	1378.52(240.59,3075.27)	3.83	74.20(11.78,184.20)	127.14(22.26,278.35)	2.04(1.80,2.29)
Panama	male	313.06(52.07,718.01)	746.10(127.23,1791.58)	1.38	42.40(7.08,97.27)	37.44(6.42,89.52)	-0.51(-0.62,-0.40)
Papua New Guinea	male	634.31(101.66,1645.74)	2631.33(455.64,6816.43)	3.15	66.53(10.76,173.11)	103.62(17.79,263.00)	1.60(1.50,1.69)
Paraguay	male	201.49(32.31,485.41)	1337.45(213.68,3220.20)	5.64	19.65(3.15,47.11)	51.43(8.24,123.37)	2.87(2.44,3.31)
Peru	male	1029.88(162.46,2575.56)	2393.94(381.18,6243.53)	1.32	18.68(2.93,46.32)	15.88(2.54,41.29)	-0.47(-0.94,0.00)
Philippines	male	6408.48(1010.46,15760.76)	14881.27(2272.16,38519.85)	1.32	46.85(7.40,114.58)	42.65(6.61,109.52)	-1.10(-1.38,-0.82)
Poland	male	28030.84(4515.15,67634.53)	50708.45(8814.72,117586.19)	0.81	146.69(23.59,352.81)	165.15(28.64,383.69)	0.33(0.22,0.45)
Portugal	male	3880.78(629.67,9018.09)	8672.82(1469.61,19780.48)	1.23	61.48(9.96,142.82)	87.88(14.68,201.42)	1.41(1.02,1.79)

Puerto Rico	male	976.84(169.59,2234.16)	1501.42(282.37,3449.98)	0.54	57.74(10.01,132.46)	48.15(8.99,111.29)	-0.85(-1.07,-0.63)
Qatar	male	47.37(8.09,114.53)	536.48(107.16,1225.18)	10.32	93.58(16.95,217.92)	100.65(20.99,213.95)	0.40(-0.11,0.91)
Romania	male	7186.33(1151.10,17477.46)	14958.65(2336.96,35886.21)	1.08	52.53(8.36,127.70)	94.72(14.85,225.90)	1.85(1.62,2.07)
Russian Federation	male	43240.70(6621.04,108080.93)	50025.08(8110.95,122424.36)	0.16	63.06(9.61,158.36)	53.32(8.64,130.71)	-0.96(-1.28,-0.65)
Rwanda	male	212.36(33.77,559.77)	433.67(65.58,1252.38)	1.04	17.15(2.65,45.01)	19.30(2.95,54.77)	-0.21(-0.50,0.09)
Saint Lucia	male	21.04(3.65,48.69)	57.17(10.40,128.10)	1.72	55.57(9.89,128.00)	55.50(10.19,123.68)	0.00(-0.19,0.19)
Saint Vincent and the Grenadines	male	10.21(1.74,23.43)	28.77(5.03,65.37)	1.82	32.14(5.49,73.51)	40.34(7.09,91.76)	0.47(0.27,0.68)
Samoa	male	16.29(2.79,39.51)	36.39(6.43,86.77)	1.23	37.33(6.50,89.52)	50.07(8.91,117.71)	0.96(0.83,1.09)
Sao Tome and Principe	male	8.23(1.34,19.99)	22.44(3.68,53.64)	1.73	27.98(4.45,68.13)	50.47(8.50,119.76)	2.19(2.08,2.30)
Saudi Arabia	male	849.73(132.85,2129.74)	3675.31(676.19,8399.19)	3.33	26.89(4.25,66.81)	36.60(6.73,82.65)	0.94(0.78,1.11)
Senegal	male	426.49(70.19,1080.13)	1387.19(229.45,3438.19)	2.25	26.99(4.48,68.20)	41.21(6.93,102.19)	2.11(1.75,2.48)
Serbia	male	6792.53(1093.84,16420.80)	14674.42(2557.17,35275.96)	1.16	117.35(18.96,284.07)	196.49(34.05,474.77)	1.89(1.65,2.14)
Seychelles	male	14.82(2.57,35.44)	47.34(8.24,105.53)	2.19	61.08(10.52,145.98)	93.99(16.72,208.26)	1.13(0.88,1.38)
Sierra Leone	male	90.12(12.87,236.95)	257.10(37.73,663.88)	1.85	9.30(1.34,24.32)	15.78(2.37,40.48)	2.30(2.09,2.51)
Singapore	male	1238.99(205.50,2857.87)	2097.49(353.36,4810.22)	0.69	125.29(20.89,286.24)	56.65(9.57,129.65)	-3.03(-3.23,-2.83)
Slovakia	male	3059.42(490.82,7497.71)	3828.42(627.12,9629.82)	0.25	116.01(18.61,284.33)	92.33(15.19,233.68)	-0.98(-1.22,-0.74)
Slovenia	male	1050.94(169.63,2618.78)	1741.21(297.60,4200.67)	0.66	105.87(17.03,263.19)	90.60(15.50,219.46)	-1.17(-1.56,-0.78)
Solomon Islands	male	65.59(8.23,199.00)	289.77(43.56,867.87)	3.42	82.56(11.20,245.14)	178.63(28.84,507.57)	2.88(2.74,3.02)
Somalia	male	141.94(21.05,401.92)	441.42(62.09,1279.23)	2.11	13.41(2.01,37.24)	17.16(2.39,48.68)	0.94(0.85,1.02)
South Africa	male	4341.68(698.24,11448.62)	12100.70(2037.76,28330.47)	1.79	50.12(8.11,132.09)	64.82(11.01,150.29)	0.76(0.28,1.25)
Republic of Korea	male	8485.50(1381.59,20294.08)	31105.82(4887.62,71862.42)	2.67	65.09(10.71,155.63)	77.55(12.23,179.27)	-0.28(-0.79,0.23)
South Sudan	male	197.25(29.51,546.18)	358.05(53.68,955.06)	0.82	15.75(2.37,42.94)	19.51(2.90,50.63)	0.76(0.68,0.83)
Spain	male	27228.55(4580.16,63156.80)	43973.04(7428.35,101690.97)	0.61	110.96(18.72,257.58)	108.26(18.10,250.98)	-0.62(-0.90,-0.34)
Sri Lanka	male	1037.91(173.65,2509.49)	5532.63(962.12,13862.81)	4.33	19.69(3.33,47.47)	47.15(8.27,116.86)	4.02(3.58,4.45)

Sudan	male	884.27(110.84,2949.02)	2972.17(454.63,8126.97)	2.36	18.15(2.28,60.23)	30.64(4.73,83.98)	1.80(1.73,1.87)
Suriname	male	51.95(8.60,123.94)	201.63(36.69,462.32)	2.88	42.00(6.96,99.25)	71.12(13.02,163.16)	2.04(1.76,2.32)
Eswatini	male	52.81(7.72,143.16)	166.28(25.43,449.38)	2.15	43.80(6.49,116.45)	73.42(11.61,195.94)	2.20(1.74,2.66)
Sweden	male	2552.66(415.25,6056.84)	3746.72(623.41,8610.31)	0.47	38.38(6.21,91.34)	37.01(6.11,85.14)	-0.05(-0.30,0.20)
Switzerland	male	4140.67(676.39,9976.53)	4271.48(707.42,10022.05)	0.03	94.75(15.45,228.63)	54.28(8.96,127.68)	-1.82(-1.95,-1.70)
Syrian Arab Republic	male	681.76(112.20,1654.89)	2555.26(416.59,6222.87)	2.75	24.91(4.12,60.16)	38.92(6.35,93.71)	1.52(1.32,1.72)
Taiwan (Province of China)	male	3827.64(613.75,9171.81)	12992.56(2169.02,31661.11)	2.39	43.51(7.03,103.36)	70.25(11.78,170.78)	1.37(1.05,1.69)
Tajikistan	male	418.08(63.18,1027.10)	831.53(138.63,2027.79)	0.99	31.49(4.80,77.25)	34.39(5.62,83.58)	1.13(0.50,1.77)
United Republic of Tanzania	male	497.45(73.81,1318.75)	1953.74(295.65,5392.24)	2.93	9.98(1.50,25.82)	18.10(2.76,50.09)	2.47(2.21,2.72)
Thailand	male	8197.88(1311.16,19892.59)	25907.70(4195.98,64912.29)	2.16	50.62(8.05,121.85)	56.11(9.19,140.01)	-0.43(-0.71,-0.14)
Bahamas	male	38.06(6.11,90.64)	106.01(18.28,248.65)	1.79	57.09(9.31,135.45)	58.32(10.06,135.91)	-0.01(-0.11,0.09)
Gambia	male	15.00(2.20,38.46)	68.78(11.15,167.43)	3.58	8.82(1.29,22.54)	16.11(2.58,38.78)	1.99(1.79,2.19)
Timor-Leste	male	33.02(4.75,85.65)	231.69(38.11,552.96)	6.02	26.79(3.92,68.20)	57.76(9.57,136.87)	2.54(2.25,2.84)
Togo	male	70.62(10.76,181.81)	322.00(48.65,826.62)	3.56	13.12(2.00,33.63)	23.86(3.73,61.15)	2.29(2.08,2.50)
Tonga	male	23.83(4.07,58.05)	52.44(9.62,121.25)	1.20	90.55(15.22,220.67)	145.27(26.80,335.74)	1.69(1.39,2.00)
Trinidad and Tobago	male	230.54(41.86,522.01)	536.18(96.44,1293.40)	1.33	57.53(10.47,130.03)	57.50(10.36,137.96)	-0.22(-0.36,-0.07)
Tunisia	male	1815.48(294.25,4477.36)	7346.52(1300.65,17960.19)	3.05	68.62(11.07,168.05)	116.42(20.50,283.92)	1.82(1.53,2.11)
Turkey	male	18038.09(2932.10,45503.98)	51780.60(8751.94,126314.18)	1.87	102.76(16.58,256.31)	122.48(20.67,295.11)	2.84(1.56,4.15)
Turkmenistan	male	271.41(42.85,671.75)	528.66(88.85,1281.98)	0.95	29.94(4.73,73.51)	27.58(4.60,67.23)	-0.67(-1.27,-0.06)
Uganda	male	374.28(56.96,940.11)	1118.55(177.43,2714.39)	1.99	12.50(1.92,30.92)	19.98(3.22,47.64)	1.49(1.29,1.69)
Ukraine	male	22371.45(3482.97,54916.42)	18809.49(3206.16,47434.69)	-0.16	78.95(12.36,193.03)	62.73(10.71,157.63)	-1.86(-2.24,-1.47)
United Arab Emirates	male	126.42(21.17,311.11)	1666.07(304.45,3973.16)	12.18	72.31(12.31,173.26)	84.63(16.68,195.40)	0.31(-0.20,0.82)
United Kingdom	male	46744.75(7638.76,109430.61)	56966.62(9982.35,126127.06)	0.22	118.18(19.30,277.88)	97.58(16.98,216.13)	-0.56(-0.76,-0.36)
United States of America	male	206853.17(34628.15,475323.24)	328737.47(58853.47,716797.31)	0.59	151.40(25.36,347.74)	125.96(22.50,274.99)	-0.06(-0.36,0.24)
Uruguay	male	794.57(121.38,1990.41)	2168.57(356.34,5080.17)	1.73	45.10(6.88,113.09)	96.41(15.86,226.41)	2.60(2.05,3.17)

Uzbekistan	male	1470.76(229.98,3670.00)	4289.31(687.69,10104.42)	1.92	28.47(4.51,70.88)	41.66(6.72,96.08)	1.28(1.14,1.42)
Vanuatu	male	22.05(3.23,61.62)	107.69(17.88,274.20)	3.88	62.21(9.34,171.97)	117.90(19.50,296.74)	2.22(2.11,2.32)
Venezuela (Bolivarian Republic of)	male	2211.22(357.18,5167.90)	9275.35(1577.66,22660.97)	3.19	49.52(8.03,114.69)	66.87(11.49,162.78)	1.18(0.80,1.56)
Viet nam	male	7376.54(1133.85,19204.00)	31944.85(5087.35,77915.16)	3.33	43.32(6.68,112.33)	81.97(13.26,198.27)	2.16(2.08,2.24)
Virginia	male	5218.96(848.09,12347.97)	8578.74(1564.77,20354.44)	0.64	170.22(27.58,402.01)	128.21(23.36,304.02)	-0.54(-0.83,-0.25)
Yemen	male	468.15(65.25,1382.72)	1859.29(286.18,5111.29)	2.97	20.39(2.95,59.97)	29.58(4.66,80.06)	1.48(1.37,1.60)
Zambia	male	238.25(36.18,608.92)	764.23(123.96,1926.84)	2.21	16.98(2.56,43.07)	26.68(4.38,66.56)	1.43(1.18,1.67)
Zimbabwe	male	539.98(88.22,1312.59)	1119.63(184.61,2723.38)	1.07	27.65(4.48,66.62)	39.28(6.57,94.17)	1.17(1.00,1.33)
Monaco	male	20.84(3.12,51.99)	64.26(11.15,154.14)	2.08	72.32(10.86,182.59)	150.47(25.53,362.78)	2.87(2.50,3.23)
San Marino	male	10.78(1.66,26.68)	25.93(4.15,68.75)	1.41	69.21(10.61,171.22)	88.20(13.94,234.56)	0.89(0.84,0.94)
Saint Kitts and Nevis	male	6.67(1.17,15.22)	14.96(2.61,33.95)	1.24	39.93(6.96,91.33)	44.81(7.88,100.77)	-0.21(-0.48,0.07)
Cook Islands	male	8.06(1.33,19.18)	20.53(3.71,46.04)	1.55	124.41(20.38,293.47)	164.92(29.80,370.37)	0.84(0.67,1.02)
Nauru	male	2.24(0.33,5.90)	3.65(0.58,9.57)	0.63	119.51(17.87,306.28)	191.54(32.09,480.24)	1.58(1.47,1.68)
Niue	male	1.14(0.19,2.67)	1.88(0.36,4.22)	0.64	123.51(20.91,286.90)	187.52(35.87,416.44)	1.47(1.31,1.63)
Palau	male	5.48(0.88,13.18)	19.59(3.44,44.17)	2.58	112.27(18.16,267.29)	171.47(30.49,382.58)	1.48(1.33,1.62)
Tokelau	male	0.36(0.06,0.91)	0.62(0.11,1.54)	0.71	57.65(9.39,144.45)	92.86(16.78,227.88)	1.79(1.70,1.89)
Tuvalu	male	2.50(0.40,6.83)	6.03(1.04,14.42)	1.41	83.00(13.50,227.36)	124.16(21.30,297.10)	1.42(1.35,1.48)
